# Switchable Valence States in Dinuclear Cobalt Complexes: The Role of Halogenated Catecholates and Counterions

**DOI:** 10.1021/acsomega.5c05045

**Published:** 2025-06-30

**Authors:** Tim W. Hieke, Sriram Sundaresan, Luca M. Carrella, Eva Rentschler

**Affiliations:** Department Chemie, 9182 Johannes Gutenberg-Universität Mainz, Duesbergweg 10-14, 55128 Mainz, Germany

## Abstract

We present the synthesis and comprehensive characterization of a series of eight dinuclear cobalt complexes, **C1–C8**, with the general formula [Co_2_(**L**)­(X_4_cat)_2_]^2+^(A)_1–__2_, wherein X = Br or Cl, A = SO_4_^2–^, ClO_4_^–^, PF^6–^, or B­(Ph)_4_^–^, and **L** denotes a redox inactive bis-tetradentate bridging ligand. Single-crystal X-ray diffraction at 120 K confirms a low-spin Co­(III) configuration in all compounds. SQUID magnetometry also shows that the complexes remain diamagnetic below room temperature. However, the complexes bearing sulfate anions, **C1** and **C5**, exhibit a distinct thermally induced valence tautomeric transition above room temperature, marked by an increase in magnetic moment. The temperature of this transition is strongly influenced by the electronic properties of the catecholate (cat) ligands, with electron-deficient tetrahalogenated catecholates stabilizing the low-spin state. In addition, counterions and solvent molecules are found to modulate intermolecular interactions in the solid state. Comparative cyclic voltammetry with previously reported di*tert*-butyl catecholate (dbucat) complex **C9** highlights the influence of ligand electronics on redox potentials, with electron-deficient catecholates shifting redox processes to higher potentials. These results highlight the tunability of cobalt valence tautomerism and redox behavior through strategic ligand and counterion selection.

## Graphical abstract

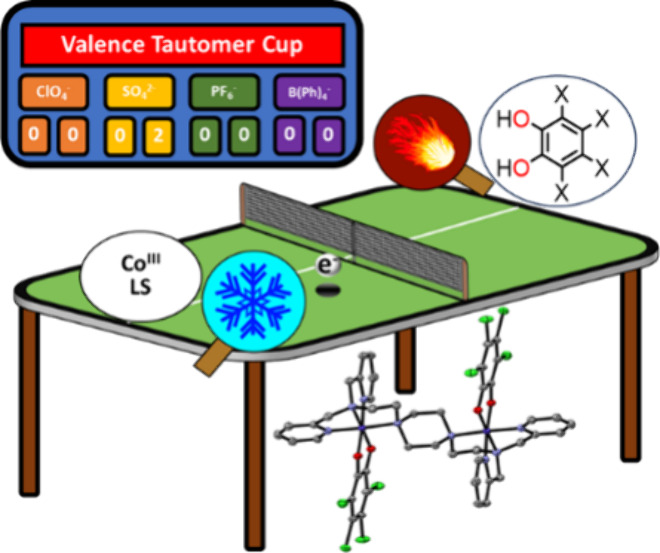

## Introduction

Bistable molecules are at the forefront of next-generation materials, offering tunable properties crucial for advanced applications. These include molecular-scale memory elements capable of switching between distinct states under external stimuli, as well as adaptive sensors that respond dynamically to environmental changes such as temperature shifts or chemical exposure. Such innovations hold immense potential for smart materials in fields ranging from environmental monitoring to biomedical diagnostics.^1–7^ Among bistable systems, metal complexes featuring open-shell ions are particularly intriguing, as changes in their electronic structure can induce significant modifications in their physical properties. Three key classes of bistable metal complexes exhibit this multistability: (i) spin-crossover (SCO) complexes (ii) Prussian-blue analogues and (iii) valence tautomeric (VT) complexes. While SCO compounds-especially those based on Fe­(II), Fe­(III), and Co­(II)-have been extensively studied for decades,^6–13^ research into valence tautomerism has gained momentum only in recent years.^14–18^ Understanding and controlling these bistable systems is essential for unlocking their full potential in molecular electronics, data storage, and responsive materials.

Prussian Blue analogues represent a versatile class of bistable materials. It Is evident that Fe/Co Prussian blue analogues are particularly noteworthy as exemplary systems for metal-to-metal charge transfer (MMCT). These analogues exhibit remarkable switchable magnetic and optical properties, thus making them valuable models for research in this field. These systems have been the focus of significant research over the past two decades, owing to their noteworthy physicochemical properties that are being investigated for their use in potential molecular electronic devices.^19–23^

Valence tautomerism was first identified in 1980 by Buchanan and Pierpont on mononuclear cobalt-dioxolene complexes.^24^ Unlike in spin crossover (SCO) systems, valence tautomeric (VT) behavior requires the presence of redox-active, noninnocent ligands, of which dioxolenes are a prominent example. For a complex to exhibit valence tautomerism, it needs to contain both an electron donor and an electron acceptor, typically the metal center and the redox-active ligand. The interaction results in simultaneous changes in the oxidation states of both components, fundamentally altering the electronic structure of the complex. The defining feature for valence tautomerism therefore is an intramolecular electron transfer between a metal ion and an organic ligand.^16,25–28^ This close interplay enables reversible electronic transitions that can be triggered by external stimuli such as temperature,^29–31^ pressure^18,32^ or light^33,34^ The ability to control these transformations makes valence tautomeric systems highly attractive for applications in molecular electronics, stimuli-responsive materials, and switchable functional devices.

Cobalt complexes are the most extensively studied VT systems due to the intrinsic link between their redox transitions and associated spin state changes.^35–38^ This feature provides a distinct magnetic contrast between states, making SQUID magnetometry a powerful tool for monitoring VT transitions. While valence tautomerism has been observed in other transition metals, including those of manganese,^39–42^ iron,^43,44^ and copper,^45–47^ these often lack an accompanying spin-state change, precluding magnetic detection.^17^ In such cases, alternative spectroscopic techniques, particularly UV–vis spectroscopy, are used to probe the characteristic optical changes associated with valence tautomerism.^41^

Compared to mononuclear systems, dinuclear cobalt complexes offer a wider range of electronic states.^35,48,49^ In most reported examples, redox-active ligands act as bridging units, facilitating electronic communication between metal centers. In contrast, complexes featuring innocent bridging ligands remain largely unexplored, with only a few examples described in the literature.^16,50–52^

Furthermore, the majority of reported studies focus on di*tert*-butyl-substituted catecholates, while tetrahalogenated analogues remain relatively unexplored. Although halogen substituents - being strongly electron withdrawing—can suppress dioxolene oxidation to semiquinone (sq) and destabilize the VT equilibrium, they also increase the air stability of the resulting complexes.^16^ Only a handful of mononuclear cobalt complexes bearing tetrachlorinated (Cl_4_-cat)^38,53–57^ or tetrabrominated catecholates (Br_4_-cat)^41,58,59^ have been reported, and cases of reversible switching behavior among them are extremely rare.

To the best of our knowledge, no dinuclear cobalt complexes with tetrahalogenated catechols have been reported to date. In this study, we present the synthesis and characterization of a family of eight cobalt complexes featuring tetrahalogenated catechol ligands. These complexes vary in the choice of catechol ligand and the uncoordinated counterions. The primary goal was to investigate the effect of electronic and structural modifications on valence tautomerism (VT) and redox properties. Additionally, we took advantage of this cationic complex family in the tunability of VT behavior through counterion selection and thus varying lattice solvent effects, which modulate intermolecular interactions, which are critical to VT transitions. To assess the impact of electron-withdrawing halogen substituents, we compare these new complexes to our previously reported di*tert*-butyl catecholate analogues. This comparative approach quantifies the influence of electron-withdrawing versus electron-donating groups on the redox properties and VT behavior of cobalt complexes.

## Results and Discussion

### Ligand Synthesis

The bridging ligand used for complexes **C1**–**C8** was synthesized according to our previously reported procedure,^60^ as shown in Scheme 1. However, the final purification step was modified to improve both the yield and purity of the product. Instead of the original washing step with acetone, a double recrystallization was carried out using 10 mL of acetone. The ligand was obtained as yellow crystals with a final yield of 85% and was fully characterized by a number of techniques including infrared spectroscopy (Figures S1–S3), ^1^H (Figures S12–S14), ^13^C NMR spectroscopy (Figure S15) and 2D-NMR techniques (Figures S16–S18). 2D NMR allowed us to assign ^13^C signals more precisely.

**Scheme 1:**
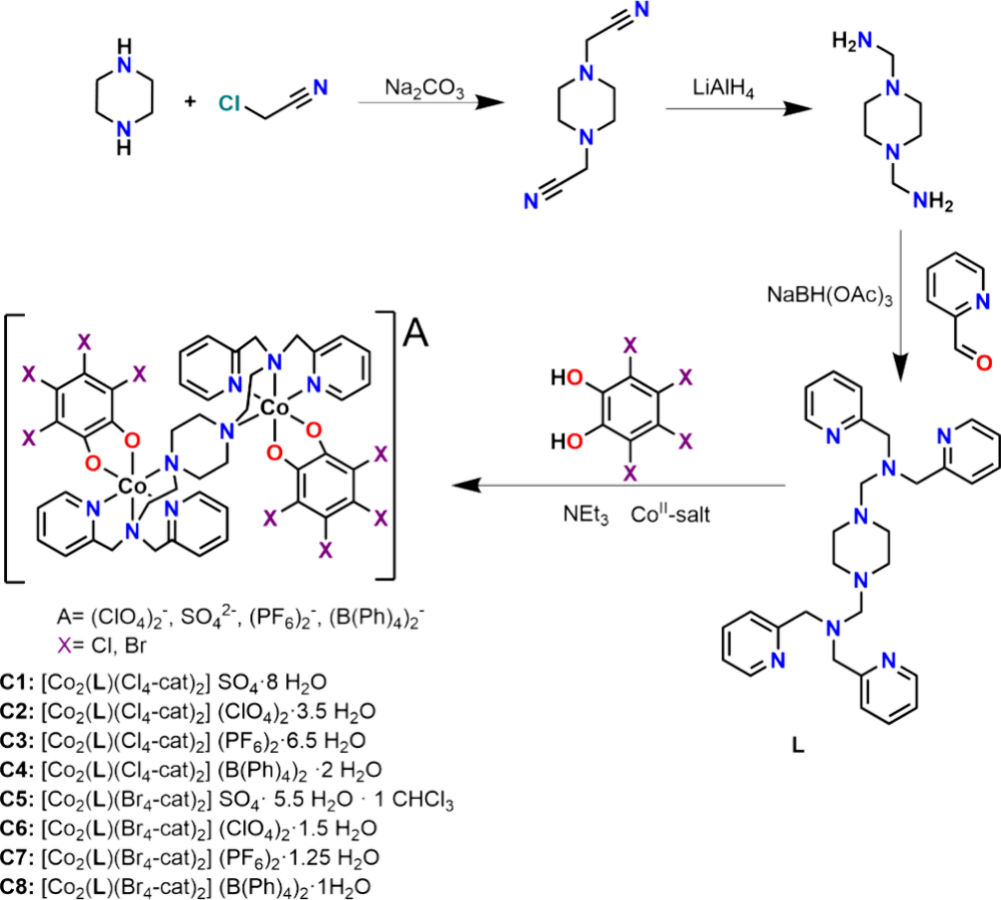
Reaction Scheme of the Multistep Ligand Synthesis of the Bis-Tetradentate Ligand *N*,*N*,*N*′,*N′*Tetra-2-picolyl-1,4-bis­(2-aminoethyl)­piperazine (**L**) and Complex Synthesis of **C1–C8**

### Complex Synthesis

The detailed synthetic procedures for the complexes **C1–C8** can be found in the experimental section. In general, the cobalt­(II)-salt (0.2 mmol, 2.0 equiv), **L** (0.1 mmol, 1.0 equiv) and the corresponding tetrahalogenated catechol (0.2 mmol, 2.0 equiv) were dissolved in 15 mL of the respective solvent. When using cobalt­(II) chloride, a mixture of acetonitrile and methanol (20 mL, 1:1) was used. A solution of triethylamine (0.4 mmol, 4.0 equiv) in 1 mL of the solvent used was added dropwise and an immediate color change to green was observed. After heating for an hour under reflux, the reaction mixture was cooled down to room temperature and filtered. Single crystals, suitable for X-ray diffraction analyses, were obtained by slow evaporation of the mother liquor over one to 15 days. When hexafluorophosphate or tetraphenylborate salts were used, the precipitates were recrystallized to obtain single crystals suitable for X-ray diffraction.

Single crystals were used for structural determination and further analytical studies. The bulk identity was verified using infrared spectroscopy, which identified characteristic bands of the anions (perchlorate, sulfate, tetraphenyl borate, and hexafluorophosphate) at their specific wavenumbers, along with the C–O vibrations of catechols at approximately 1425 cm^–1^ (Figures S4–S11). High-resolution mass spectrometry confirmed the presence of the dicationic fragment [M]^2+^ for all complexes (**C1**–**C8**), with the observed isotopic patterns precisely matching theoretical calculations, verifying the formation of dinuclear cobalt compounds (Figures S22–S37). Elemental analysis further confirmed the bulk purity of the samples and revealed that air exposure led to the replacement of lattice solvents with water molecules, resulting in the formation of hydrated complexes.

### Crystal Structures Description

The structures of **C1–C8** have been determined by single crystal X-ray diffraction at 120 K. Figure 1 shows the structures of the complexes [Co_2_(**L**)­(Cl_4_cat)_2_]­SO_4_, **C1–Cl–SO**_**4**_, and [Co_2_(**L**)­(Br_4_cat)_2_]­SO_4_, **C5–Br–SO**_**4**_, while the remaining structures can be found in the SI (Figures S38–S45).

**Figure 1:**
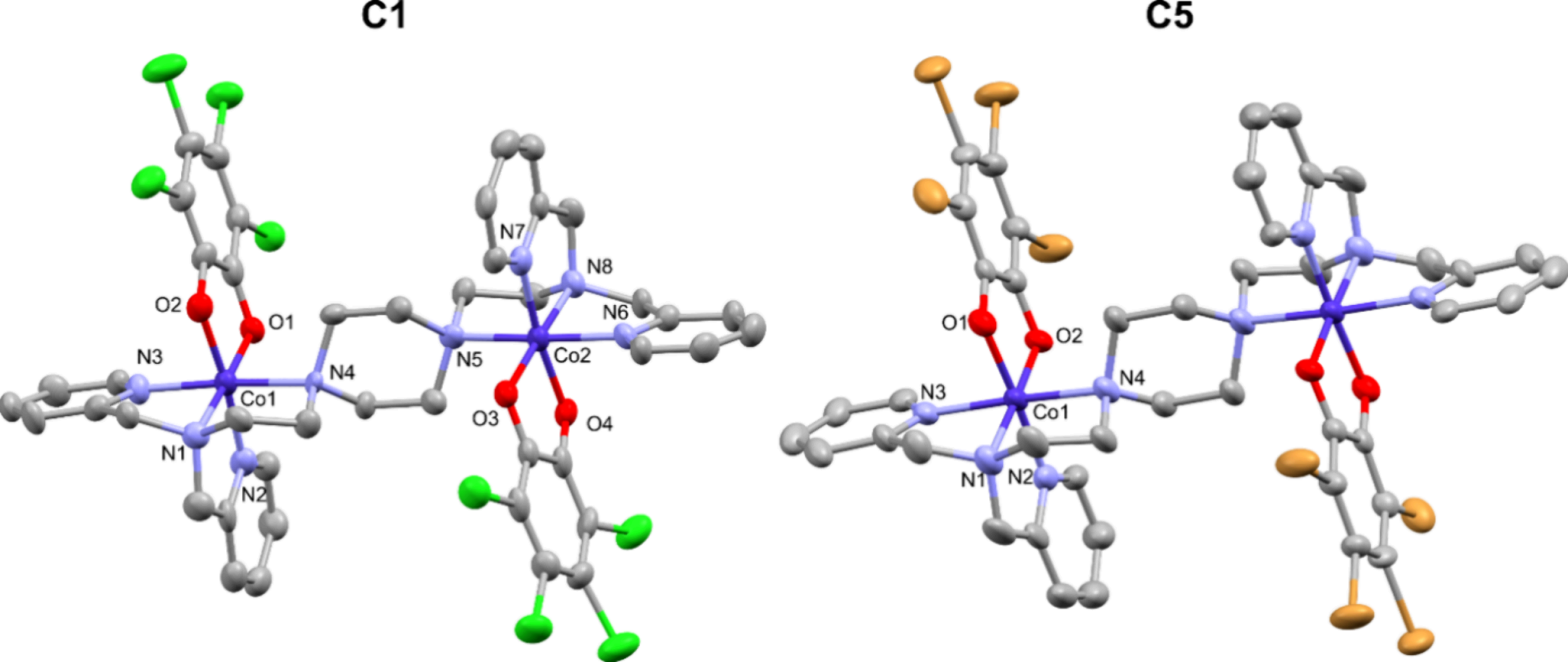
Complex structures of [Co_2_(**L**)­(Cl_4_cat)_2_]­SO_4_, **C1–Cl–SO**_**4**_, and [Co_2_(**L**)­(Br_4_cat)_2_]­SO_4_, **C5–Br–SO**_**4**_ at 120 K. Lattice solvent molecules, anions, and hydrogen atoms are omitted for clarity. The probability level for the displacement of the ellipsoid is 50%. Color code: carbon (gray), cobalt (dark blue), nitrogen (light blue), oxygen (red), chloride (green), bromide (brown).

Complexes **C1–Cl–SO**_**4**_, **C4–Cl–BPh**_**4**_ crystallized in the centrosymmetric P 2_1_/c space group, **C8–Br–BPh**_**4**_ in the monoclinic P 2_1_/n space group, while complexes **C2–Cl–ClO**_**4**_ **, C3–Cl–PF**_**6**_ **, C5–Br–SO**_**4**_, **C6–Br–ClO**_**4**_ and **C7–Br–PF**_**6**_ crystallized in the triclinic P1̅ space group with only one molecule in the unit cell. The molecular structure of the isostructural complexes shows a dinuclear cobalt complex in which the two metal centers are coordinated by a bis-tetradentate bridging ligand. The coordination sphere of each cobalt ion is completed by the bidentate, deprotonated *ortho*-dioxolene ligand. In detail, the N_4_O_2_ coordination environment of each cobalt center is composed as follows: Two nitrogen atoms originate from pyridine units, another from an aliphatic tertiary amine, while the fourth comes from the piperazine heterocycle. The two ortho oxygen atoms of the deprotonated catechol ligand complete the coordination. The octahedral coordination geometry around each cobalt ion is only slightly distorted, likely due to the rigidity of the bridging ligand. The metal donor bond lengths are 1.8708–1.912 Å for Co–O, and 1.902–1.957 Å for Co–N for the three nitrogen donor atoms of the capping side arms. The bond lengths of Co–N for the piperazine nitrogen donor are always significantly longer with metal donor distances of 2.0376–2.107 Å. Details can be found in Table 1. The expected metal donor bond distances are 1.926–1.955 Å for Co - N and 1.877 −1.880 Å for Co–O in similar low spin Co­(III) complexes as well as 2.125–2.243 Å for Co–N and 1.988 −2.171 Å for Co–O in similar high spin Co­(II) complexes.^61^ The software program *SHAPE 2.1* ^62^ was utilized to conduct continuous shape measurements. The cobalt centers of complexes **C1–8** exhibited little deviations from the ideal octahedral coordination geometry, suggesting a small distortion. The coordination environments exhibit *SHAPE* indices ranging from 0.23856 to 0.41186. The closer the index is to 0, the closer the coordination environment is to the ideal geometry. It is possible to discern the small distortions in the structures of the compounds, as can be seen by the nitrogen atom of the piperazine. This atom always exhibits longer bond distances in comparison to the other metal–nitrogen bonds. The complete *SHAPE* calculations are listed in Tables S20–S29. The “metrical oxidation states” (MOS) for each of the attached catecholates were calculated based on all C–O and C–C ring distances in the dioxolene ligand.^26^ The values which were obtained for the catecholates range from −1.69 to −1.91 which corresponds to catecholate ligands. The complete values for the MOS can be found in Tables S30. The metal donor distances clearly show that the cobalt ions are in the low spin *d* ^*6*^ state.^63,64^ This is in accordance also with the C–C bond lengths of the catechols which vary between 1.375 and 1.438 Å and indicate aromatic C–C bonds as expected for catecholates. Thus, at 120 K the cobalt ions for all complexes are trivalent and the noninnocent ligand is in its catecholate state. All bond lengths, together with the bond angles, are listed in Tables S5–S19.

**Table 1:** Metal-Donor Bond Lengths of Complexes **C1–C8** in Å at 120 K

Co-X	C1–Cl–SO_4_	C2–Cl–ClO_4_	C3–Cl–PF_6_	C4–Cl–BPh_4_	C5–Br–SO_4_	C6–Br–ClO_4_	C7–Br–PF_6_	C8–Br–BPh_4_
Co1–O1	1.881(6)	1.898(4)	1.8816(14)	1.8708(13)	1.912(5)	1.898(5)	1.8722(18)	1.8773(13)
**Co1–O2**	1.898(6)	1.871(4)	1.8966(15)	1.8909(15)	1.893(5)	1.882(4)	1.8918(18)	1.8745(13)
**Co1–N1**	1.933(8)	1.936(4)	1.9399(16)	1.9454(17)	1.957(6)	1.942(5)	1.937(2)	1.9460(15)
**Co1–N2**	1.924(7)	1.910(4)	1.9081(18)	1.9200(18)	1.925(7)	1.902(6)	1.915(2)	1.9170(16)
**Co1–N3**	1.922(8)	1.916(4)	1.9171(17)	1.9173(19)	1.937(6)	1.923(6)	1.914(2)	1.9251(15)
**Co1–N4**	2.043(7)	2.048(4)	2.0479(17)	2.0555(17)	2.056(6)	2.107(6)	2.047(2)	2.0376(15)
**Co2–O3**	1.889(6)	1.895(4)						
**Co2–O4**	1.895(6)	1.873(4)						
**Co2–N5**	2.045(8)	1.938(4)						
**Co2–N6**	1.919(8)	1.921(4)						
**Co2–N7**	1.926(8)	1.914(4)						
**Co2–N8**	1.928(7)	2.045(4)						

The intermolecular interactions for each of the eight complexes are investigated in detail. Complex **C5–Br–SO**_**4**_ shows π-π interactions between aromatic pyridines with a centroid-to-centroid distance of 3.820 Å, a parallel shift of 1.743 Å and an angle of 0° between both pyridine planes (Figure S53). The remaining complexes do not exhibit any π-π interactions between aromatic compounds. However, every complex is dicationic in nature and therefore crystallizes with its respective anion: **C1–Cl–SO**_**4**_, **C5–Br–SO**_**4**_: sulfate, **C2–Cl–ClO**_**4**_, **C6–Br–ClO**_**4**_ **:** perchlorate, **C3–Cl–PF**_**6**_, **C7–Br–PF**_**6**_: hexafluorophosphate, **C4–Cl–BPh**_**4**_, **C8–Br–BPh**_**4**_ **:** tetraphenylborate. Moreover, all single crystals of the complexes are grown from the mother liquor and contain lattice solvent molecules: **C1–Cl–SO**_**4**_·11 MeOH, **C2–Cl–ClO**_**4**_·3 H_2_O·1 MeCN, **C3–Cl–PF**_**6**_·2MeOH·2 MeCN, **C4–Cl–BPh**_**4**_·1 MeCN, **C5–Br–SO**_**4**_·4 MeOH·2 H_2_O, **C6–Br–ClO**_**4**_·2 MeCN, **C7–Br–PF**_**6**_·1 MeOH·1 MeCN, **C8–Br–BPh**_**4**_·4 MeCN. Complex **C1–Cl–SO**_**4**_, with 11 methanol molecules, has the largest number of solvent molecules per complex ion, and it is therefore not surprising that it also has the strongest hydrogen bond network (Figure S54), especially considering that the sulfate anion is doubly negatively charged. For the analogue Br_4_-cat complex, **C5–Br–SO**_**4**_, with 4 methanol molecules, this interaction is already less pronounced (Figure S56). In contrast, as expected, the complexes bearing MeCN solvent molecules (**C4–Cl–BPh**_**4**_, **C6–Br–ClO**_**4**_, **C8–Br–BPh**_**4**_) do not show any hydrogen bonding interactions. For the other complexes the hydrogen bonding network is found to be small to moderate. Worth noting in this context is the H···F interaction for the hexafluorophosphate anions being close to nonacidic CH groups of the complex cations in **C3–Cl–PF**_**6**_ and **C7–Br–PF**_**6**_ (Figures S57 and S58).

The flexibility of the crystal lattice, which allows volume expansion during tautomeric valence transitions, plays a crucial role in their behavior. Complexes with minimal lattice solvent have a rigid structure. In **C1–Cl–SO**_**4**_ and **C5–Br–SO**_**4**_, by contrast, several methanol molecules fill empty spaces in the packing and engage in extensive intermolecular interactions. This allows the formation of a rich hydrogen bonding network which makes the lattice more flexible and facilitates valence tautomeric transitions as will be discussed in more detail below. In detail, **C1–Cl–SO**_**4**_ shows a herringbone structure when viewed along the *a*-axis (Figure S59), but resembles a star-shaped motif when viewed in the a-b plane because of the alternating orientations of the complexes along the *c*-axis (Figure 2). The same cation packing motif is found for **C3–Cl–PF**_**6**_.

**Figure 2:**
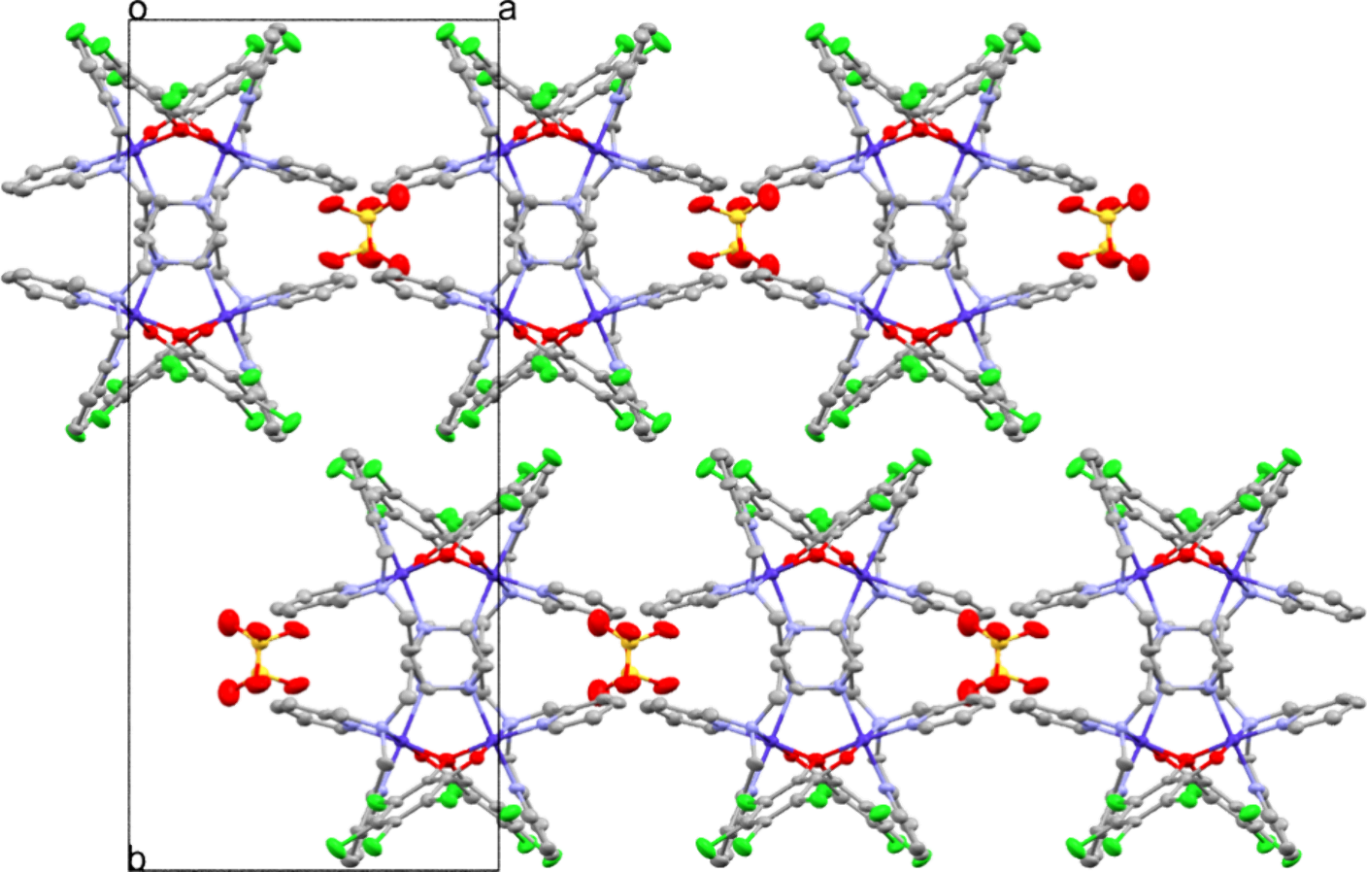
Crystal packing of **C1–Cl–SO**_**4**_ viewed along the *c* axis of the crystal. Lattice solvent molecules and hydrogen atoms are omitted for clarity.

### Electrochemistry

Cyclic voltammograms were obtained for solutions of compounds **C2–Cl–ClO**_**4**_·3.5 H_2_O, **C6–Br–ClO**_**4**_·1.5 H_2_O and our previously reported **C9** [Co_2_(**L**)­(dbucat)_2_]­(ClO_4_)_2_·1.5 H_2_O^60^ (Figure 3) due to solubility problems with the complexes **C1–Cl–SO**_**4**_ and **C5–Br–SO**_**4**_. It is evident from the results of the UV–vis and Evans method discussed in the later part of the manuscript that all complexes **C2–Cl–ClO**_**4**_, **C6–Br–ClO**_**4**_, and **C9-dbucat-ClO**_**4**_ are in the LS-Co­(III)-cat state at room temperature in acetonitrile solution. Electrochemical studies were carried out on 1 mM dry acetonitrile solutions with 0.1 M *n*-Bu_4_PF_6_ using a glassy carbon working electrode and a silver wire pseudoreference electrode. As an internal reference, all measurements were calibrated against the ferrocene/ferrocenium (Fc/Fc^+^) redox couple by adding ferrocene after the last measurement of a series. The half-wave potentials (*E*_1/2_) and peak-to-peak separations (Δ*E*_p_) are given in Table 2, together with the peak potentials (*E*_p_) for the irreversible processes.

**Figure 3:**
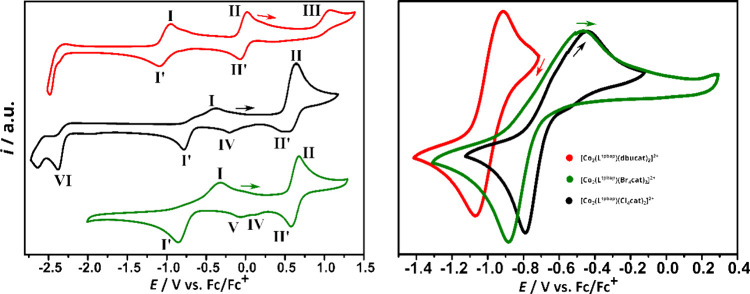
Cyclic voltammograms (left) and comparison of redox process (**I/I′**) (right) for compounds [Co_2_(**L**)­(Br_4_cat)_2_]­(ClO_4_)_2_ **·**1.5 H_2_O **C6** (green), [Co_2_(**L**)­(Cl_4_cat)_2_]­(ClO_4_)_2_ **·**3.5 H_2_O **C2** (black) and the previously reported [Co_2_(**L**)­(dbucat)_2_] (ClO_4_)_2_·1.5 H_2_O **C9** (red) (1.0 mM with 0.1 M *n*-Bu_4_PF_6_ in acetonitrile obtained with a scan rate of 100 mV s^–1^) with a glassy carbon working electrode, a silver wire reference electrode, referenced internally against the redox pair ferrocene/ferrocenium (Fc/Fc^+^).

**Table 2:** Cyclic Voltammetry Data for **C9-dbucat-ClO**_**4**_ ·1.5 H_2_O, **C2–Cl–ClO**_**4**_·3.5 H_2_O, and **C6–Br–ClO**_**4**_·1.5 H_2_O in Acetonitrile^a^

	cyclic voltammetry data *E*_m_ or *E*_p_ /V (Δ*E*_p_/mV) vs. Fc/Fc^+^.					
compound	I/I′	II/II′	III	IV	V	VI
C9-dbucat-ClO_4_ 1.5 H_2_O	–1.022(147)	–0.027(91)	1.070			
C2–Cl–ClO_4_·3.5 H_2_O	–0.578(408)	0.574(139)		–0.206		–2.378
C6–Br–ClO_4_·1.5 H_2_O	–0.585(537)	0.591(95)		0.160	–0.065	

a *E*_m_ is the experimentally determined average potential between the potentials of the corresponding anodic and cathodic events of a reversible or quasi-reversible electrochemical event, while *E*_p_ is the peak potential of an irreversible electrochemical event.

The previously reported **C9-dbucat-ClO**_**4**_·1.5 H_2_O and **C2–Cl–ClO**_**4**_·3.5 H_2_O were suitable for measuring over the entire acetonitrile solvent window, whereas the stability of complex **C6–Br–ClO**_**4**_·1.5 H_2_O only allowed us to measure in the range of −2 to 1.5 V vs Fc/Fc^+^. Each complex showed distinct redox processes denoted as (**I/I′**) and (**II/II′**), corresponding respectively to the redoxpair of Co­(II)/Co­(III) and to the redoxpair catecholate/semiquinonate, which were assigned according to the literature.^59,65,66^ During the initial cycle of the cyclic voltammetry experiments, the peak that corresponds to the oxidation of Co­(II) to Co­(III) was absent. Only after the first reduction process (**I**) did (**I′**) appear, confirming that this redox process is cobalt centered, since at room temperature the cobalt centers should be in the trivalent oxidation state. The scan rate investigation (Figures S83–S85) reveals that all redox processes are quasi-reversible, as the peak potentials are shifted away from each other with increasing scan rates. Furthermore, the differences in peak potentials are significantly greater than 59 mV, confirming the quasi-reversible nature. In the case of the previously reported complex **C9-dbucat-ClO**_**4**_·1.5 H_2_O the redox process (**III**) is attributed to the irreversible oxidation of the semiquinonate to the quinoate. The irreversibility of the redox process (**III**) can be attributed to the weak coordination of quinones, which consequently leads to dissociation.^65^ Within the measured potential range, complexes **C2–Cl–ClO**_**4**_·3.5 H_2_O and **C6–Br–ClO**_**4**_·1.5 H_2_O do not show a redox process (**III**). Furthermore, as expected, the potentials of the redox processes (**I/I′**) and (**II/II′**) occur at more anodic voltages as expected for the electron-withdrawing chloro and bromo substituents bearing catecholates (Figure 3).

The lack of electron density in the catecholates due to the negative inductive effects of the halogen substituents in the case of the tetrahalogenated catecholate complexes promotes the reduction process and hinders the oxidation process. As a result, process (**III**) becomes inaccessible for both complexes **C2–Cl–ClO**_**4**_·3.5 H_2_O and **C6–Br–ClO**_**4**_·1.5 H_2_O. However, both **C2–Cl–ClO**_**4**_·3.5 H_2_O and **C6–Br–ClO**_**4**_·1.5 H_2_O show the irreversible reduction processes (**IV**) and (**V**), which might be ligand-based. These processes occur only after the oxidation process (**II**) at the elevated potential and do not appear when the window is narrowed down to scan in the region where process (**I/I′**) occur, as can be seen in the scan-rate studies (Figures S83–S85). In addition, the redox process (**VI**) represents an irreversible reduction event. The reduction events at these negative potentials are not of any relevant interest to the current studies. However, at these negative potentials and, taking into account the presence of electron-withdrawing substituents, the reduction can either be due Co­(II) being reduced to Co­(I) species or, most likely, an irreversible ligand reduction.^67^ It has been demonstrated that the shapes of the redox process **(I/I′)** differ for the tetrahalogenated and the di-*tert*-butyl catecholate cyclic voltammograms. It is evident that the peak difference is intensified for the tetrahalogenated catecholates. A larger peak separation is generally associated with higher barriers to electron transfers due to lower kinetics, and therefore the need for more extreme potentials. It appears that tetrahalogenated catecholates stabilize the LS-Co­(III)-cat state more strongly, resulting in a slower electron transfer and, consequently, a broader peak separation. In contrast, the complex with di-*tert*-butyl-catecholate demonstrates a reduced peak separation, indicative of a faster and electrochemical reversible process.^68^

Another interesting value determined by cyclic voltammetry is the potential gap between redox processes **I/I′** and **II/II′**. It is evident that these processes are associated with the Co­(II)/Co­(III) and the cat/sq redox processes. This is precisely what occurs during the VT transition. It can thus be concluded that, in order to minimize the energy required to overcome the activation barrier of the switching behavior, the potential gap must be reduced to the smallest possible level. **C9-dbucat-ClO**_**4**_ exhibits the narrowest gap, with a difference of 996 mV, while **C2–Cl–ClO**_**4**_ demonstrates a wider gap of 1152 mV and **C6–Br–ClO**_**4**_ shows a gap of 1176 mV. It is evident that the electron-donating force on the catecholate diminishes the potential gap, thereby favoring the VT behavior.

### Magnetic Characterization

The temperature dependent magnetic susceptibility for complexes **C1–C8** was measured in polycrystalline samples in a SQUID magnetometer from 200 to 380 K, as shown as the χ_M_ *T* product in Figure 4. In all cases the complexes remain diamagnetic below 300 K, as expected for a Co^III^_2_(cat)_2_ species. This is further supported by the metal to ligand donor distances observed in the X-ray structure data at low temperatures. The complexes **C2–C4** and **C6–C8** retain their diamagnetic character up to 380 K and show no evidence of switching behavior. The individual magnetic susceptibility measurements for each complex are given in the Supporting Information (Figures S86–S93).

**Figure 4:**
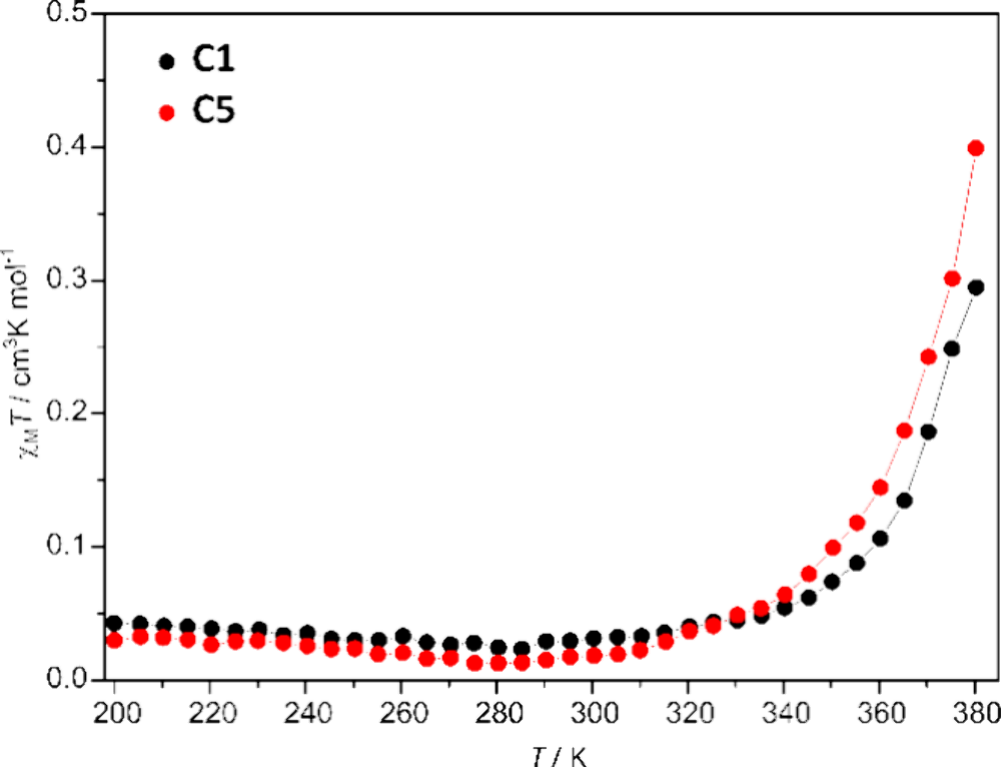
Temperature dependent magnetic susceptibility of compounds **C1–Cl–SO**_**4**_ and **C5–Br–SO**_**4**_ over the temperature window of 200–380 K.

In the case of complexes **C1–Cl–SO**_**4**_ and **C5–Br–SO**_**4**_, a gradual subtle increase in magnetic moment occurs above room temperature, indicating the transition from the low spin (LS) Co^III^(cat) entity to the high spin (HS) Co^II^(sq) configuration.^16,51,69^ The small increase in the magnetic moment to 0.294 and 0.399 cm^3^ K mol^–1^ at 380 K for **C1–Cl–SO**_**4**_·8 H_2_O and **C5–Br–SO**_**4**_·5.5 H_2_O·CHCl_3_ respectively is attributed to this transition, although the transition is incomplete. According to the spin-only values, a peak spin-only χ_M_ *T* value of 2.25 cm^3^ K mol^–1^ at elevated temperature is expected for an HS Co­(II) (with a spin value of 3/2) and a semiquinone radical (with a spin value of 1/2).^70^ Due to orbital angular momentum of the ^4^T_1_ state in Co­(II) even higher magnetic moments can be observed.^16,70–72^ It is worth noting that the complexes **C2–C4** and **C6–C7** also show a small increase in the magnetic moment above room temperature, but the increase is too small and the slope is too weak to allow a valence tautomerism transition to be established. For the complexes **C1–Cl–SO**_**4**_ and **C5–Br–SO**_**4**_ we observe higher onset temperature of the transition in comparison to [Co_2_(**L**)­(dbucat)_2_]­SO_4_,^60^ as expected for a change from electron-donating to electron-withdrawing tetrahalogen substituents in the catechols, and since chlorine is more electronegative than bromine, even more so for the latter (Figure 4). This delay clearly shows that the electron-deficient tetrahalogenated catechols are reluctant to participate in intramolecular electron transfer compared to the di-*tert*-butyl catechols. Compared to the di-*tert*-butyl catechols. Thus, while the transition begins at around 340 K for the complexes with the tetrahalogenated catechols, it does not begin for the di-*tert*-butyl catechol complexes. To understand why only the complexes **C1–Cl–SO**_**4**_ and **C5–Br–SO**_**4**_ exhibit a significant VT, while this is not the case for the others, we have to go back to the discussion of the crystal packing, which differs in the series of complexes with different counteranions. The valence tautomeric transition from the LS Co­(III) to the HS Co­(II) state is always accompanied by a change in the volume of the crystal lattice. The changes in metal–ligand bond lengths are significant because antibonding eg* orbitals are occupied upon transition from the LS to the HS state. The Co–N and Co–O bond lengths can increase by up to 0.15 Å.^56^ For this to happen, the crystal lattice needs to be flexible and less rigid. The complexes **C1–Cl–SO**_**4**_·8 H_2_O and **C5–Br–SO**_**4**_·5, 5 H_2_O·1 CHCl_3_ are the only ones with only a single counterion for the divalent sulfate ion, which makes intermolecular cation–anion interactions quite different from those of the complexes with two monovalent anions. The looser packing of the molecules in the crystal is reflected in the larger number of solvent molecules found in the unit cell. As mentioned above, the crystal packing for **C1–Cl–SO**_**4**_ and **C5–Br–SO**_**4**_ has up to eight water molecules as lattice solvents, making the lattice more flexible than for the compounds with the other anions.

The ligand field strength, which was altered by the various substituted catechols was clearly visible in the oxidation potential studies by cyclic voltammetry and can also be seen in the magnetic data. When the complexes with the same counterion, **C9-dbucat-ClO**_**4**_, **C2–Cl–ClO**_**4**_ and **C6–Br–ClO**_**4**_, are compared, a temperature shift of the valence tautomeric transition is observed for the three catechols with varying electron-withdrawing substituents due to the different resulting ligand field strengths.

### Solution Studies

To eliminate packing effects affecting VT behavior, electronic properties of the complexes in solution were investigated. Solution stability and structural integrity in solution were investigated by DOSY-NMR (Figures S19–S21). The observation of a single signal in the DOSY spectrum indicates that the cationic fragment of the complex diffuses as a unit and does not dissociate further. Electronic absorption spectra (Figure 5) were recorded for **C2–Cl–ClO**_**4**_, **C6–Br–ClO**_**4**_, and **C9-dbucat-ClO**_**4**_ at room temperature in acetonitrile. The spectra of **C2–Cl–ClO**_**4**_ and **C6–Br–ClO**_**4**_ are very similar and exhibit three absorption bands only differing in its intensities were the tetrabrominated catecholate complex shows higher intensities while in **C9-dbucat-ClO**_**4**_ the bands are shifted to lower energies due to the electron-rich di-*tert*-butyl catecholates. The first two absorption bands at 233 and 319 nm for **C2–Cl–ClO**_**4**_, 236 and 318 nm for **C6–Br–ClO**_**4**_ and 299 and 378 nm for **C9-dbucat-ClO**_**4**_ are ligand based π-π* transitions from the pyridine and catecholate fragments. Both complexes also exhibit a weak transition at 654 nm for **C2–Cl–ClO**_**4**_, 652 nm for **C6–Br–ClO**_**4**_ which is shifted for **C9-dbucat-ClO**_**4**_ to 781 nm which has been described in the literature as symmetry forbidden Co­(III)­cat LMCT.^65^ All three UV–vis spectra are characteristic for low spin Co­(III)-cat species.^59^

**Figure 5:**
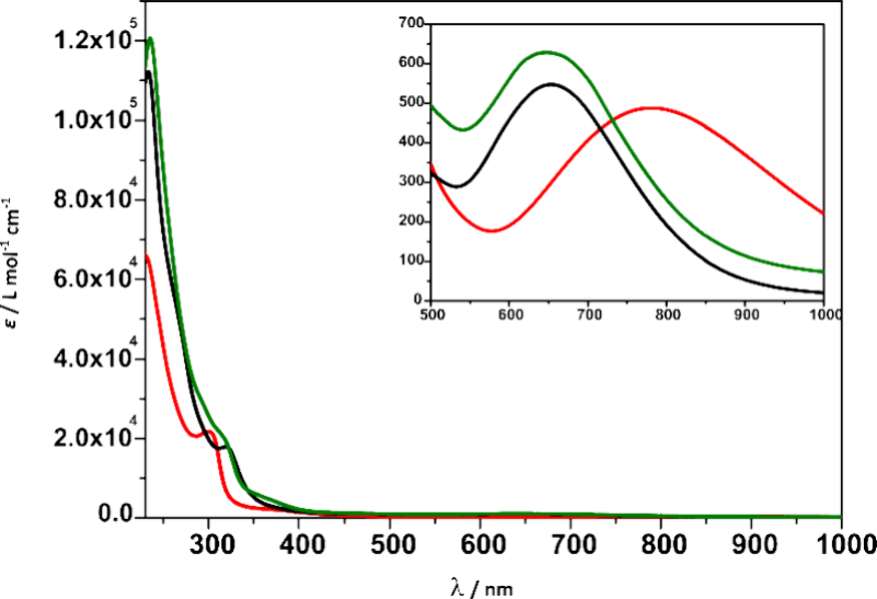
UV–vis absorption spectra for acetonitrile solutions of **C2–Cl–ClO**_**4**_ (black) 0.1 mM, **C6–Br–ClO**_**4**_ (green) 0.01 mM, and **C9-dbucat-ClO**_**4**_ (red) 0.01 mM with a zoomed-in section from 500 to 1000 nm at 1 mM concentration for all complexes.

The paramagnetic behavior of **C9-dbucat-ClO**_**4**_ was probed in solution using the variable temperature Evans ^1^H NMR method between 298 and 343 K in 5 K steps. Measurements were performed in deuterated acetonitrile at a concentration of 6 mM. The Evans method has a relative error of approximately 5–10%.^73–75^ As shown in Figure S100 and detailed in Table S31, the complex stays diamagnetic with no significant paramagnetic shifts until 318 K. Above this temperature, a prominent shift emerges, and at the maximum temperature of 343 K the χ_M_ *T* value reaches a maximum of 0.478 cm^3^ K mol^–1^, indicating a valence tautomeric transition in solution. In contrast, Evans method measurement of **C3–Cl–PF**_**6**_ and **C7–Br–PF**_**6**_ under the same conditions (6 mM in acetonitrile) did not show any evidence of a paramagnetic shift (Figures S101 and S102). These results suggest that the tetrahalogenated complexes do not undergo valence tautomeric transitions in acetonitrile solution. Unfortunately, due to solubility problems the complexes could not be measured in alternative solvents that might favor the less polar Co­(II)-semiquinonate state.^76^

## Conclusion and Outlook

We have synthesized a family of eight novel dinuclear cobalt complexes **C1–C8** featuring a nonredox active bridging ligand and tetrahalogenated redox-active dioxolene coligands. Single crystal X-ray diffraction at 120 K confirmed that all complexes adopt low spin Co­(III) states, consistent with the diamagnetic behavior observed in SQUID measurements up to room temperature. Above room temperature, complexes **C1–Cl–SO**_**4**_ and **C5–Br–SO**_**4**_, containing sulfate counterions, exhibit a temperature-dependent increase in χ_*M*_T, suggesting an incomplete valence tautomeric (VT) transition within the accessible temperature range. To the best of our knowledge, these are the first reported VT active dinuclear cobalt complexes with tetrahalogenated catechols. Electrochemical studies on **C2–Cl–ClO**_**4**_ **, C6–Br–ClO**_**4**_ and the previously reported **C9-dbucat-ClO**_**4**_ reveal significant shifts in the oxidation potentials highlighting the influence of catechol substitution on redox properties. Complementary UV–vis spectroscopy shows a bathochromic shift in LMCT absorption for the di-*tert*-butyl substituted catecholate, further supporting ligand field modulation. Investigations are currently underway to build on this preliminary evidence for valence tautomeric transitions in this system by fine-tuning the design of the ligand to achieve a complete transition in this system, centered around room temperature.

### Materials and Methods

The commercially available chemicals were used without any further purification methods and were purchased from Sigma, Alfa Aesar, Acros Organics, VWR and Fisher Chemicals. Anhydrous and degassed acetonitrile was purchased from Acros Organics and used for CV measurements. All reactions were carried out aerobic conditions with HPLC grade solvents unless otherwise stated. Dry THF was obtained from a solvent purification system (MBRAUN MG-SPS-800). Infrared spectra were recorded at room temperature in a range of 4000–450 cm^–1^ using a Bruker ALPHA II ATR-IR with the Software *OPUS* and plotted using *Origin V7.5.* Crystallographic characterization of suitable single crystals of the compounds were measured on a *STOE IPDS 2T* or on a *STOE STADIVARI* at the Johannes Gutenberg-University Mainz at 120 K. Solving and refinement of the crystal structures was done by *ShelXT* ^77^ and *ShelXL* ^78^ in combination with *Olex 2.* ^79^ The resolved are deposited at the CCDC database with reference numbers CCDC 2444030–37. HRes ESI mass spectra were recorded on *Agilent 6200 series TOF/6500 series G-TOF* (11.0.203.0) at Johannes Gutenberg-University Mainz in acetonitrile or methanol. For cyclic voltammetry measurements a PGSTAT potentiostat with a TSC 1600 was used with an already built-in platinum counter electrode as a vessel wall from *rhd* instruments. The working electrode was a glassy carbon electrode which was polished before every measurement using aluminum oxide polishing paste with grain sizes of 0.1 and 0.05 μm from *Buehler* for at least half an hour on a microfiber cloth in figures of 8’s. The pseudo reference electrode was a silver wire electrode. Every measurement was referenced against ferrocene/ferrocenium by adding ferrocene at end of the measurement. Tetrabutylammonium hexafluorophosphate was used in a concentration of 0.1 M as the conducting salt. The magnetic measurements were done with a *Quantum Design MPMS XL Squid magnetometer*. The samples were prepared in a gelatin capsule without eicosane and placed in a plastic straw and the magnetic data were obtained from a temperature range of 200–380 K under an applied magnetic field of 0.1 T in the heating mode. Measurements were performed over a length of 4 cm with 24 data points, every data point was measured three times and an average was made. Magnetic contributions of the holder were experimentally determined by a test run of an empty capsule and subtracted from the measured susceptibility data. Molar susceptibility was calculated using julX 1.4.1 made by Bill using the molecular weight of the sample for complexes **C1, C2, C5** and **C6**.^80^ While complexes **C3, C4, C7** and **C8** were measured with a *MPMS3 Squid magnetometer.* The samples were prepared in plastic capsules without eicosane placed in a brass holder and the magnetic data were obtained from a temperature range of 200–380 K under an applied magnetic field of 0.1 T in the heating mode. Measurements were performed over a length of 4 cm with 24 data points, every data point was measured three times and an average was made. Magnetic contributions of the holder and capsule were experimentally determined by a test run of an empty capsule at the exact same position with the exact same brass sample holder and subtracted from the measured susceptibility data with the software Squidlab.^81^ The elemental analysis (CHN) was performed at the Department of Chemistry at the Johannes Gutenberg-University Mainz on an Elementar vario EL Cube. NMR spectra as well as the Evans method NMR studies were conducted using an *Avance III HD 300* (*v*(^1^H) = 400.13 MHz *v*(^13^C) = 100.1 MHz); *Avance II 400* (*v*(^1^H)= 400.13 MHz *v*(^13^C) = 100.1 MHz); *Bruker DRX 400* (*v*(^1^H)= 400.13 MHz *v*(^13^C) = 100.1 MHz). All samples were dissolved in suitable deuterated solvents and the given chemical shift refers to the signal against trimethylsilane as an external reference. The obtained spectra were evaluated by *MestReNova x64* made by the company *Mestrelab Research.*

### Experimental Section

#### Caution

While no issues were encountered during the course of this work, nonetheless, it is important to note that perchlorate salts have the potential to be explosive, so careful handling is advised.

Syntheses were performed similar to **C1–Cl–SO**_**4**_, the complete synthetic procedures can be found in the SI.

##### [Co_2_(**L**)­(Cl_4_cat)_2_] SO_4_·11 MeOH [**C1–Cl–SO**_**4**_]

To a solution of CoSO_4_ **·**7 H_2_O (56 mg, 0.2 mmol, 2.0 equiv), **L** (54 mg, 0.1 mmol, 1.0 equiv) and tetrachlorocatechol (Cl_4_cat) (50 mg, 0.2 mmol, 2.0 equiv) in 15 mL methanol triethylamine (40 mg, 0.4 mmol, 4.0 equiv) was added dropwise. A suspension immediately formed, consisting of an orange precipitate in a green solution. The reaction mixture was refluxed for one hour. After the reaction mixture had cooled down to room temperature, it was filtered. After 1 day of slow evaporation green crystals suitable for X-ray diffraction were obtained. The product obtained was filtered, washed three times with 5 mL of ice-cold methanol and air-dried. The desired complex was obtained in low yields (green plates, 33 mg, 0.038 mmol, 37.5%). IR **υ̃** [cm^–1^]: 3610­(w), 3170­(broad) 3071­(w), 3011­(w), 2947­(w), 1664­(w), 1608­(w), 1433­(ss), 1376­(m), 1321­(w), 1291­(w), 1252­(m), 1220­(w), 1137­(m), 1082­(m), 1052­(m), 1021­(m), 1001­(w), 970­(m), 945­(w), 866­(w), 841­(w), 824­(w), 809­(m), 798(s), 772(s), 729­(m), 716­(m), 693­(m), 665­(m), 647­(w), 611­(m), 592­(m), 550­(m), 531­(m), 490­(m), 473­(m), 457­(m), 434­(m). Elemental Analysis: Found: C, 37.77%; H, 4.11%; N, 7.96%. Calc. for C_44_H_40_Cl_8_Co_2_N_8_O_8_S **·** 8 H_2_O: C, 38.12%; H, 4.07%; N, 8.08%. Mass spectrometry [*m*/*z*]: 572.964 [**C1**]^2+^ (calc. = 572.964).

##### [Co_2_(**L**)­(Cl_4_cat)_2_]­(ClO_4_)_2_ · 3 H_2_O ·1 MeCN [**C2–Cl–ClO**_**4**_]

(Green plates, 30 mg, 0.022 mmol, 22.3%). IR **υ̃** [cm^–1^]: 3548­(m), 3073­(m), 2962­(m), 1638­(m), 1609­(m), 1462­(m), 1434(s), 1376(s), 1327­(m), 1291­(m), 1251(s), 1224­(m), 1079­(ss), 1039(s), 1023(s), 1002­(m), 972(s), 954(s), 931­(m), 893­(m), 865(s), 839­(m), 827­(m), 810(s), 798(s), 766(s), 731­(m), 716­(m), 693­(m), 665­(m), 646­(m), 623(s), 595(s), 556­(m), 530­(m), 488(s), 472­(m), 455(s), 434(s). Elemental Analysis: Found: C, 37.33%; H, 3.15%; N, 8.27%. Calc. for C_44_H_40_Cl_10_Co_2_N_8_O_12_ · 3.5 H_2_O, C, 37.53%; H, 3.36%; N, 7.96% Mass spectrometry [*m*/*z*]: 572.964 [**C2**]^2+^ (calc. = 572.964), 1244.880 [**C2**+ClO_4_]^+^ (calc. = 1244.877).

##### [Co_2_(**L**) (Cl_4_cat)_2_]­(PF_6_)_2_ · 2 MeOH · 2 MeCN [**C3–Cl–PF**_**6**_]

(Green powder 68 mg, 0.047 mmol, 47.3%). IR **υ̃** [cm^–1^]: 2954­(w), 1639­(w), 1610­(w), 1435(s), 1377­(w), 1293­(w), 1251­(m), 1025­(w), 975­(m), 955­(w), 838­(ss), 811(s), 800­(m), 766­(m), 717­(m), 694­(w), 666­(w), 597­(m), 558(s), 456­(m), 433­(m). Elemental Analysis: Found: C, 34.19%; H, 3.72%; N, 7.34% Calc. for C_44_H_40_Cl_8_Co_2_F_12_N_8_O_4_P_2_ · 6.5 H_2_O C, 34.02%; H, 3.44%; N, 7.21% Mass spectrometry [*m*/*z*]: 572.964 [**C3**]^2+^ (calc. = 572.964).

##### [Co_2_(**L**)­(Cl_4_cat)_2_]­(B­(Ph)_4_)_2_ · 1 MeCN [**C4–Cl–BPh**_**4**_]

(Green powder 56 mg, 0.031 mmol, 31.4%). IR **υ̃** [cm^–1^]: 3052­(w), 1609­(m), 1579­(w), 1478­(m), 1435(s), 1376­(m), 1290­(m), 1251­(m), 1160­(m), 1058­(w), 1025­(m), 975­(m), 943­(w), 866­(m), 812­(m), 800­(m), 767­(m), 734(s), 705(s), 665­(w), 612­(m), 598­(m), 562­(w), 470­(w), 434­(m). Elemental Analysis: Found: C, 60.34%; H, 4.74%; N, 6.43% Calc. for C_92_H_82_B_2_Cl_8_Co_2_N_8_O_4_ · 2 H_2_O: C, 60.62%; H, 4.76%; N, 6.15% Mass spectrometry [*m*/*z*]: 572.964 [**C4**]^2+^ (calc. = 572.964).

##### [Co_2_(**L**)­(Br_4_cat)_2_]­SO_4_ · 2 H_2_O · 4 MeCN [**C5–Br–SO**_**4**_]

(Green needles 19 mg, 0.012 mmol, 12.0%). IR **υ̃** [cm^–1^]: 3606­(m), 3090 (broad), 3065­(m), 3007­(m), 2958(s), 1650­(m), 1607­(m), 1459­(m), 1425­(ss), 1348(s), 1320­(m), 1290­(m), 1264(s), 1235(s), 1207(s), 1138(s), 1082(s), 1051(s), 1037(s), 1020(s), 998(s), 968­(m), 956(s), 930(s), 865(s), 841­(m), 823(s), 767­(ss), 743(s), 728(s), 715(s), 681(s), 665(s), 647(s), 608(s), 580(s), 563(s), 534(s), 519(s), 497(s), 477(s), 460(s), 453(s), 429(s). Elemental Analysis: Found: C, 29.67%; H, 3.17%; N, 6.36%, Calc. for C_44_H_40_Br_8_Co_2_N_8_O_8_S · 5.5 H_2_O · 1 CHCl_3_: C, 29.76%; H, 2.89%; N, 6.17%. Mass spectrometry [*m*/*z*]: 750.761 [**C5**]^2+^ (calc. = 750.761)

##### [Co_2_(**L**)­(Br_4_cat)_2_]­(ClO_4_)_2_ ·2 MeCN [**C6–Br–ClO**_**4**_]

(Green needles 19 mg, 0.011 mmol, 11.2%). IR **υ̃** [cm^–1^]: 3617­(m), 3542­(m), 2940­(m), 1632­(m), 1608­(m), 1462­(m), 1428(s), 1378­(m), 1346­(m), 1326­(m), 1292­(m), 1261(s), 1233(s), 1211­(m), 1079­(ss), 1038(s), 1022(s), 1001(s), 979­(m), 954(s), 930(s), 893(s), 864(s), 839­(m), 826(s), 765­(ss), 745(s), 730(s), 715(s), 686­(m), 665­(m), 647­(m), 623(s), 589(s), 566(s), 533(s), 492(s), 476(s), 460(s), 451(s), 429(s). Elemental Analysis: Found: C, 30.22%; H, 2.49%; N, 6.47%, Calc. for C_44_H_40_Br_8_Cl_2_Co_2_N_8_O_12_ · 1.5 H_2_O: C, 30.59%; H, 2.51%; N, 6.49%. Mass spectrometry [*m*/*z*]: 750.762 [**C6**]^2+^ (calc. = 750.761), 1600.470 [**C6**+ClO_4_] ^+^ (calc. = 1600.470).

##### [Co_2_(**L**)­(Br_4_cat)_2_]­(PF_6_)_2_ ·1 MeOH ·1 MeCN [**C7–Br–PF**_**6**_]

(Green plates, 56 mg, 0.031 mmol, 31.3%). IR **υ̃** [cm^–1^]: 3658­(w), 3065 (w), 2958 (w), 1637­(w), 1609­(m), 1571­(w), 1462­(m), 1427(s), 1346­(m), 1293­(m), 1260­(m), 1231­(m), 1163­(w), 1098­(w), 1084­(w), 1063­(w), 1052­(w), 1038­(w), 1024­(m), 1003­(w), 933­(m), 823(s), 764(s), 745(s), 716­(m), 665­(m), 626­(m), 587­(m), 556(s), 534­(m), 479­(m), 452­(m), 429­(m). Elemental Analysis: Found: C, 29.50%; H, 2.75%; N, 6.35%, Calc. for C_44_H_40_Br_8_Co_2_F_12_N_8_O_4_P_2_ · 1.25 H_2_O: C, 29.13%; H, 2.36%; N, 6.18%. Mass spectrometry [*m*/*z*]: 750.762 [**C7**]^2+^ (calc. = 750.761).

##### [Co_2_(**L**)­(Br_4_cat)_2_] (B­(Ph)_4_)_2_ ·4 MeCN [**C8–Br–BPh**_**4**_]

(Green powder, 190 mg, 0.089 mmol, 88.7%). IR **υ̃** [cm^–1^]: 3052­(w), 2998­(w), 2982­(w), 1608­(w), 1578­(w), 1479­(m), 1428­(ss), 1345­(w), 1290­(w), 1261­(m), 1232­(m), 1160­(w), 1058­(w), 1027­(w), 999­(w), 935­(m), 865­(w), 842­(w), 765(s), 733(s), 705­(ss), 665­(w), 611(s), 591­(m), 565­(w), 536­(w), 465­(w), 451­(w), 429­(w). Elemental Analysis: Found: C, 50.98%; H, 3.97%; N, 5.43%, Calc. for C_92_H_82_Br_8_Co_2_N_8_O_4_ · 1 H_2_O: C, 51.15%; H, 3.92%; N, 5.19%. Mass spectrometry [*m*/*z*]: 750.761 [**C8**]^2+^ (calc. = 750.761).

##### [Co_2_(**L**)­(dbucat)_2_] (ClO_4_)_2_ ·1.5 H_2_O [**C9-dbucat-ClO**_**4**_]

Was prepared as previously reported.^60^

## Supplementary Material



## Notes

IR spectra of the ligand (incl. the precursor) and complexes; ^1^H, ^13^C, COSY, HMBC, and HSQC spectra of the ligand; DOSY spectra of the complexes; synthetic details of the complexes; HRes-ESI mass spectra of the complexes; crystallographic data containing bond length tables and bond angles; SHAPE measurements; metric oxidation state assignments of the catecholates; crystal structures; packing pictures; cyclovoltammetric studies of the complexes; magnetic susceptibility measurements; UV–vis spectroscopy; and Evans NMR data of the complexes (PDF)
