# Supplementary material for: Switchable Valence States in Dinuclear Cobalt Complexes: The Role of Halogenated Catecholates and Counterions
Source: ACS Omega. 2025 Jun 30;10(27):29888–98. doi: 10.1021/acsomega.5c05045 (PMC12268467; doi:10.1021/acsomega.5c05045)
Supplement: Supplementary file 1 [file ao5c05045_si_001.pdf]

Electronic Supporting Information

Switchable Valence States in Dinuclear Cobalt Complexes: The Role of Halogenated Catecholates and Counterions

Tim W. Hieke, Sriram Sundaresan, Luca M. Carrella and Eva Rentschler*

Department Chemie, Johannes Gutenberg-Universität Mainz, Duesbergweg 10-14, 55128, Mainz, Germany

Table of Contents

1. IR-Spectra 1
2. ^{1}H-NMR 7
3. ^{13}C-NMR 9
4. COSY-NMR 10
5. HMBC-NMR 11
6. HSQC-NMR 12
7. DOSY-^{1}H-NMR-Spectra 13
8. Complex Synthesis 15
9. Mass Spectrometry 19
10. Crystallographic Data 27
11. Bond Length Tables 31
12. Metal-Donor-Bond Angles 35
13. SHAPE Measurements 38
14. Metric Oxidation State (MOS) 41
15. Crystal Structures 41
15.1 Complexes 41
15.2 Asymmetric unit cells 46
15.3 π-π interactions 50
15.4 Hydrogen Bonding Interactions 50
15.5 H-F-Bonding Interactions 52
15.6 Crystallographic planes 53
15.6.1 A-B-plane 53
15.6.2 B-C-Plane 58
15.6.3 A-C-Plane 62
16. Cyclovoltammetric Scan Rate Studies 66
17. Magnetic Susceptibility Measurements 68
18. UV-VIS-Spectroscopy 71
19. Evans Method NMR 74

1. IR-Spectra

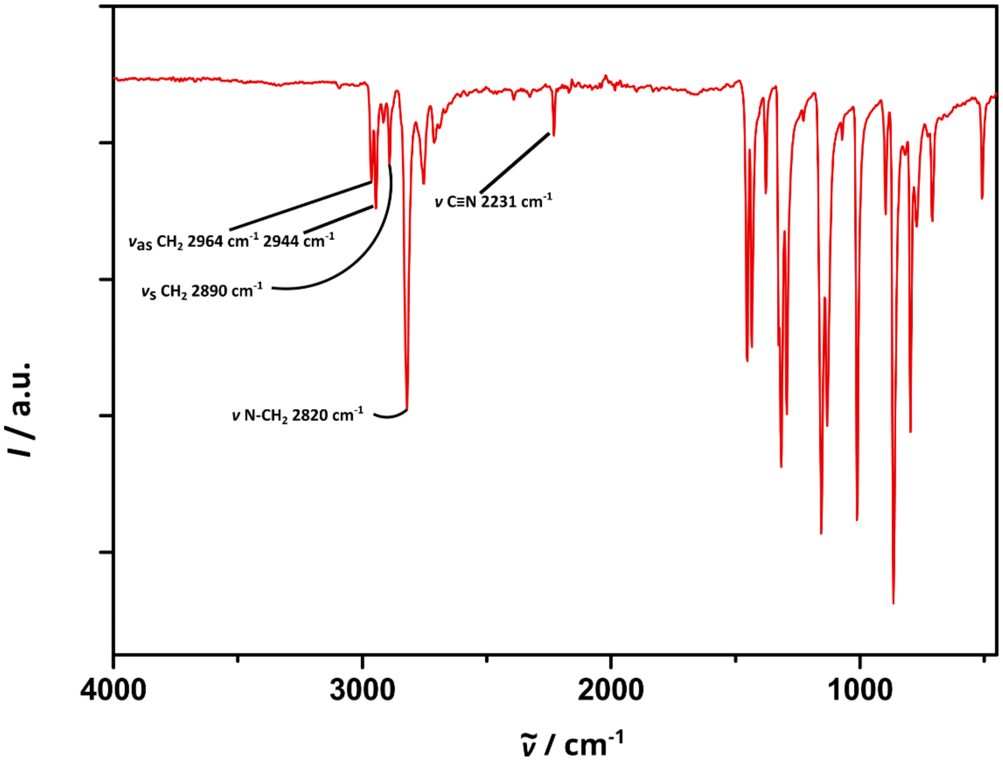

Figure S1: ATR-IR-spectrum of *N*, *N*'-Bis(cyanomethyl)piperazine at room temperature.

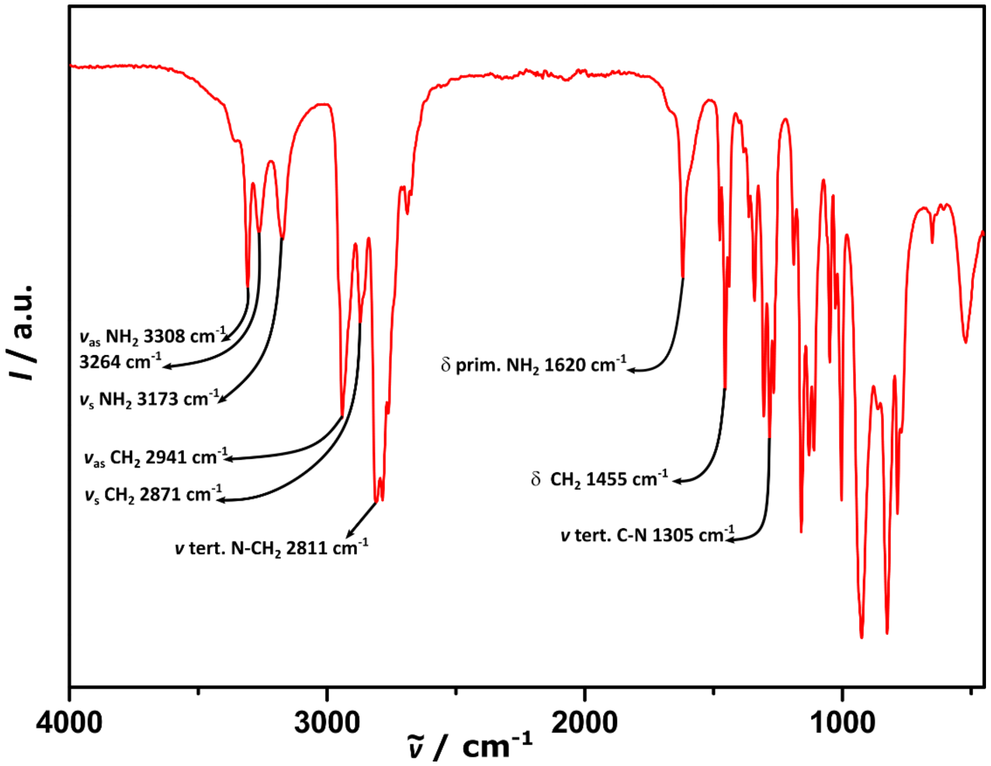

Figure S2: ATR-IR-spectrum of *N*, *N*'-bis(aminoethyl)piperazine at room temperature.

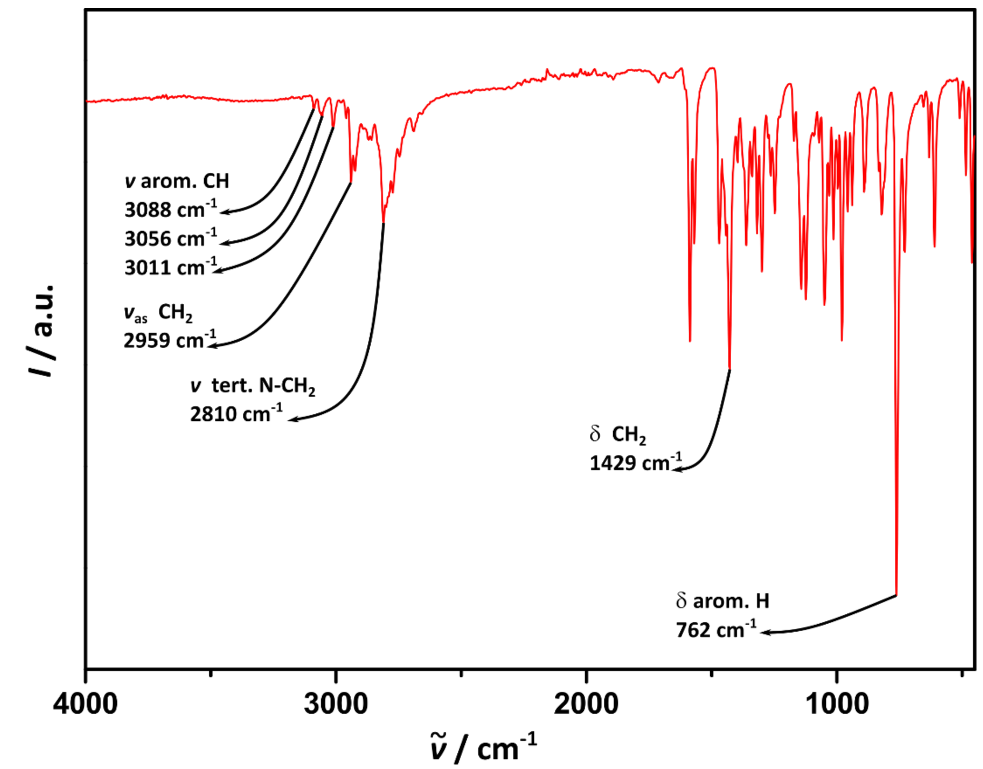

Figure S3: ATR-IR of *N*, *N*, *N*', *N*'-Tetra-2-picolyl-1,4-bis(2-aminoethyl)piperazine (**L**) at room temperature.

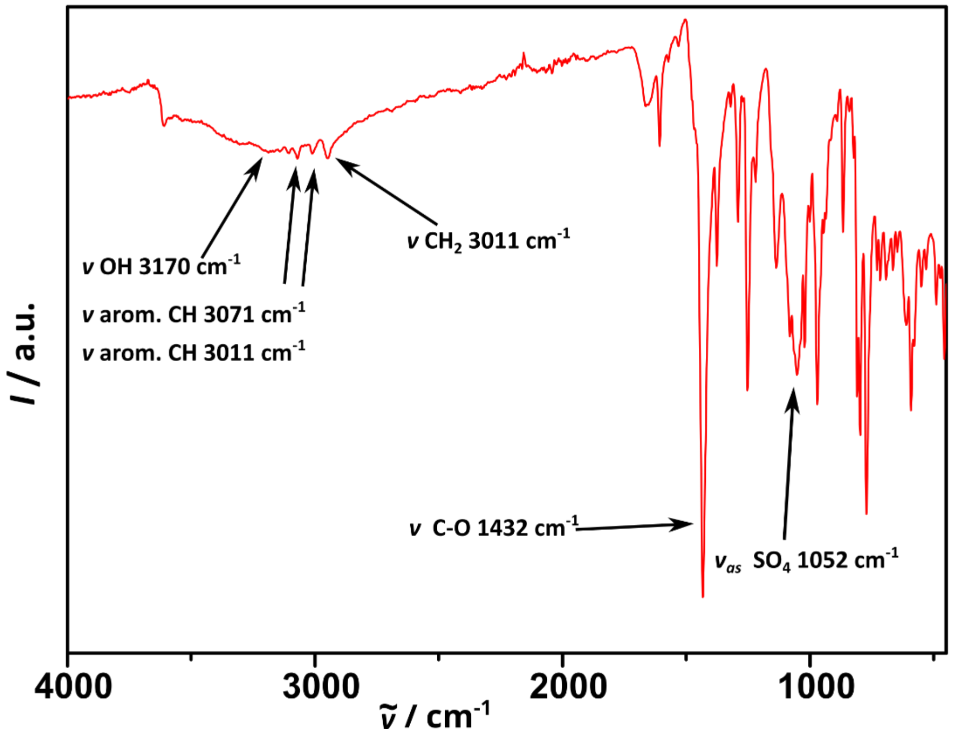

Figure S4: ATR-IR of $[Co_2$(**L**)$(Cl_4\text{-cat})_2]SO_4 \cdot 8\ H_2O$ (**C1**$\cdot$ 8 H_2O) at room temperature.

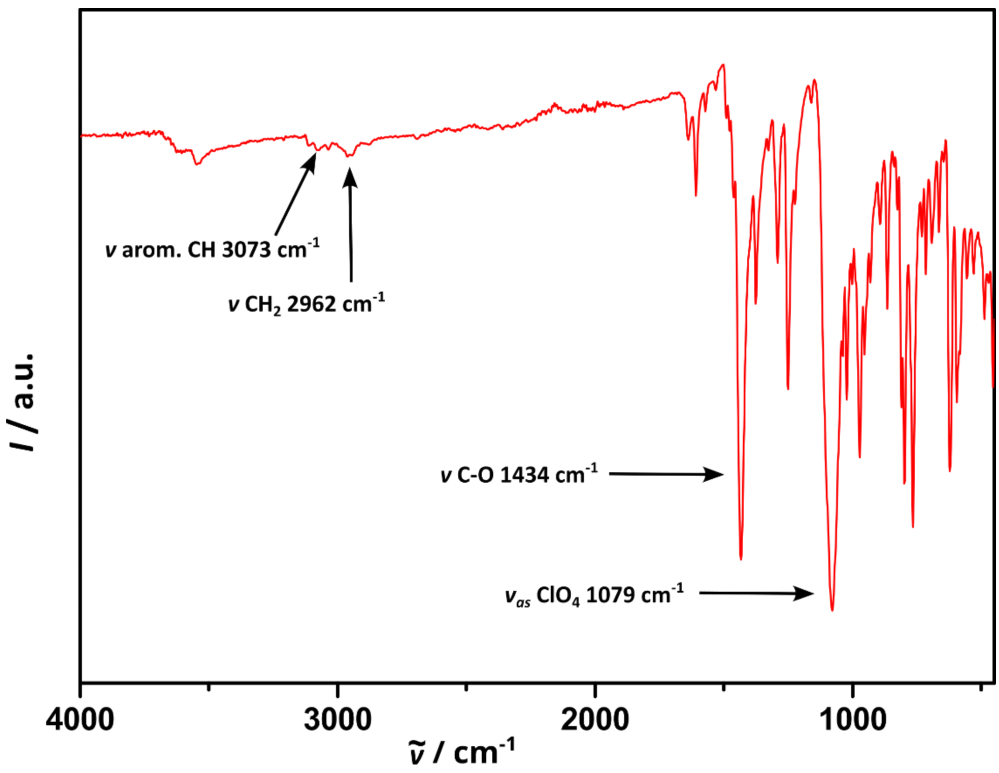

Figure S5: ATR-IR of $[Co_2(\mathbf{L})(Cl_4\text{-cat})_2]$ $(ClO_4)_2 \cdot 3.5$ H_2O (**C2**$\cdot$ 3.5 H_2O) at room temperature.

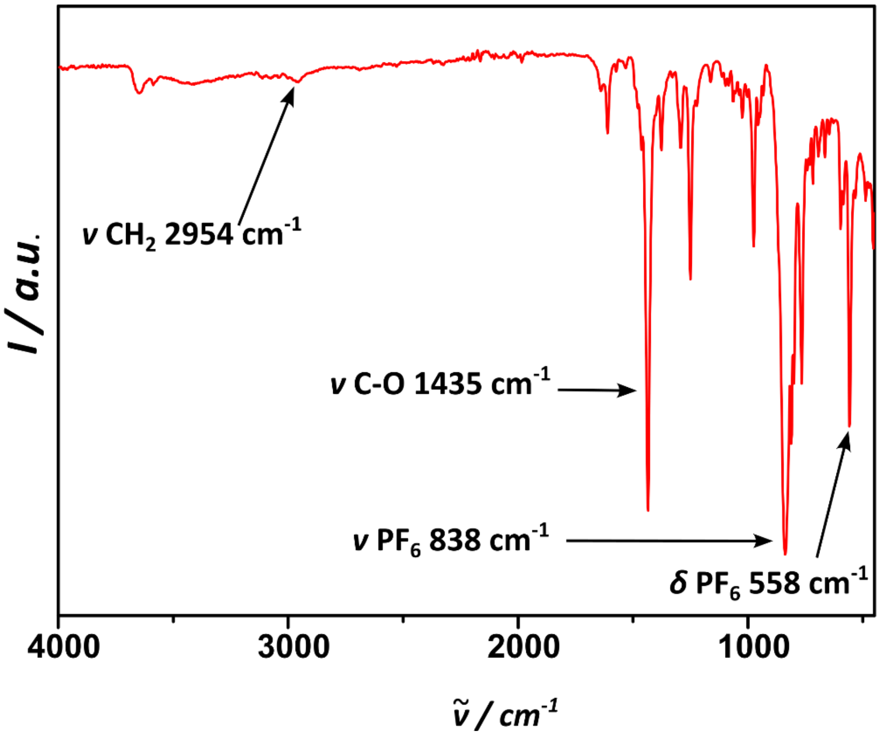

Figure S6: ATR-IR of $[Co_2(\mathbf{L})(Cl_4\text{-cat})_2]$ $(PF_6)_2 \cdot 6.5$ H_2O (**C3**$\cdot$ 6.5 H_2O) at room temperature.

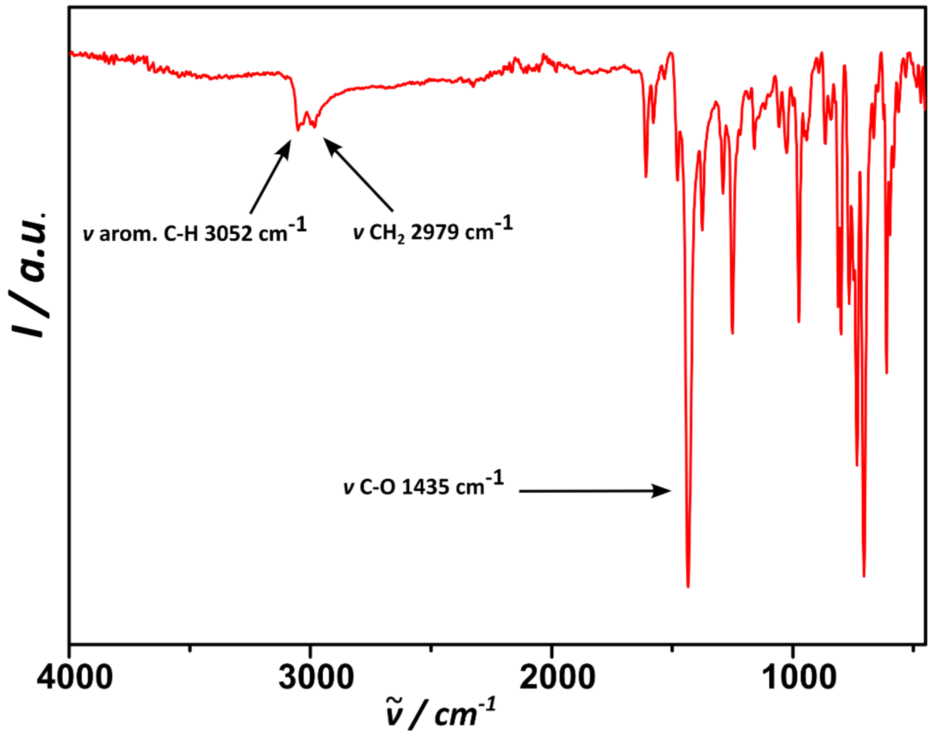

Figure S7: ATR-IR of [Co_2(**L**)(Cl_4-cat)$_2$] ($B(Ph)_4$)$_2$ · 2 H_2O (**C4**· 2 H_2O) at room temperature.

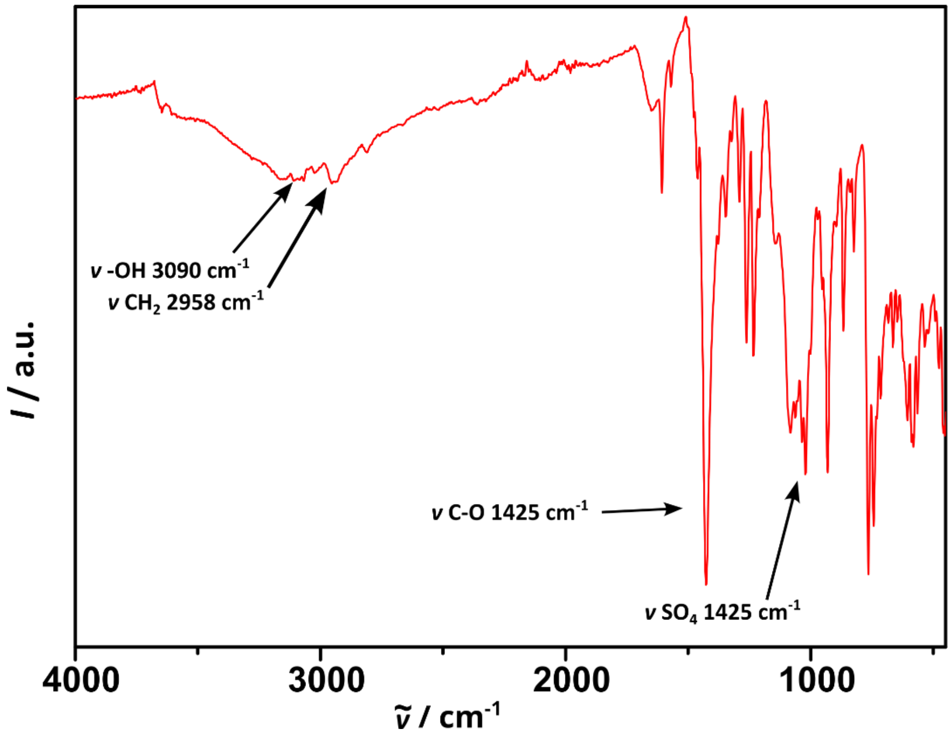

Figure S8: ATR-IR of [Co_2(**L**)(Br_4-cat)$_2$]SO_4· 5.5 H_2O · 1 $CHCl_3$ (**C5**· 5.5 H_2O · 1 $CHCl_3$) at room temperature.

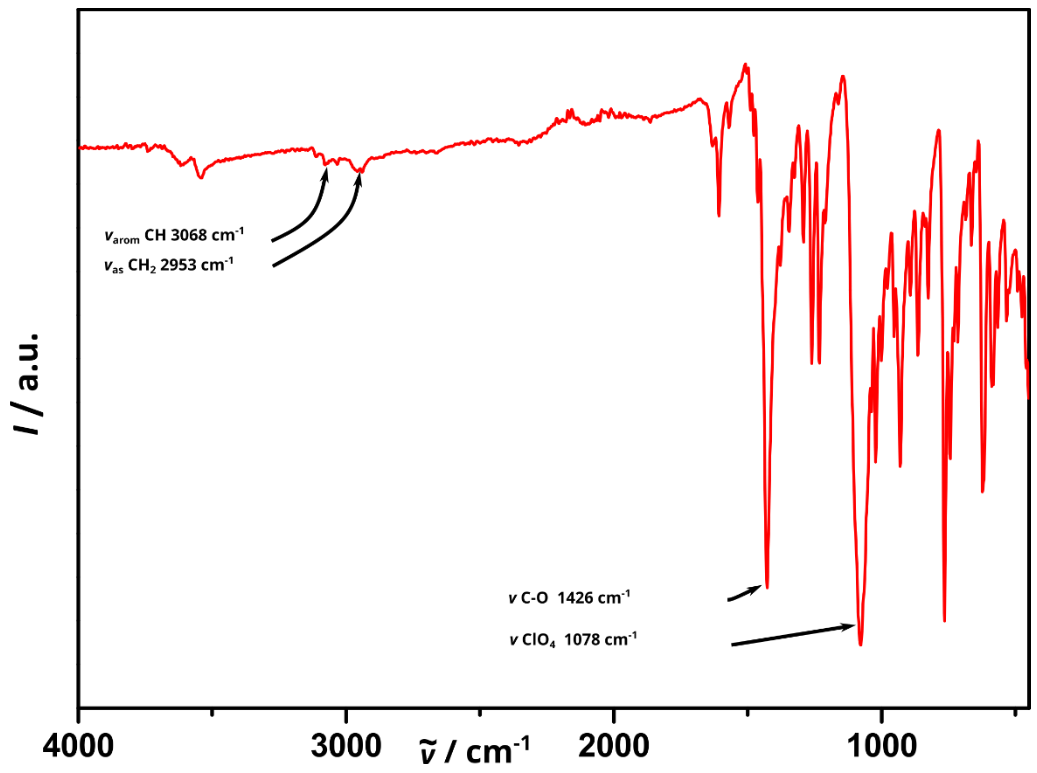

Figure S9: ATR-IR of $[Co_2(\mathbf{L})(Br_4\text{-}cat)_2]\ (ClO_4)_2 \cdot 1.5\ H_2O$ (**C6**· 1.5 H_2O) at room temperature.

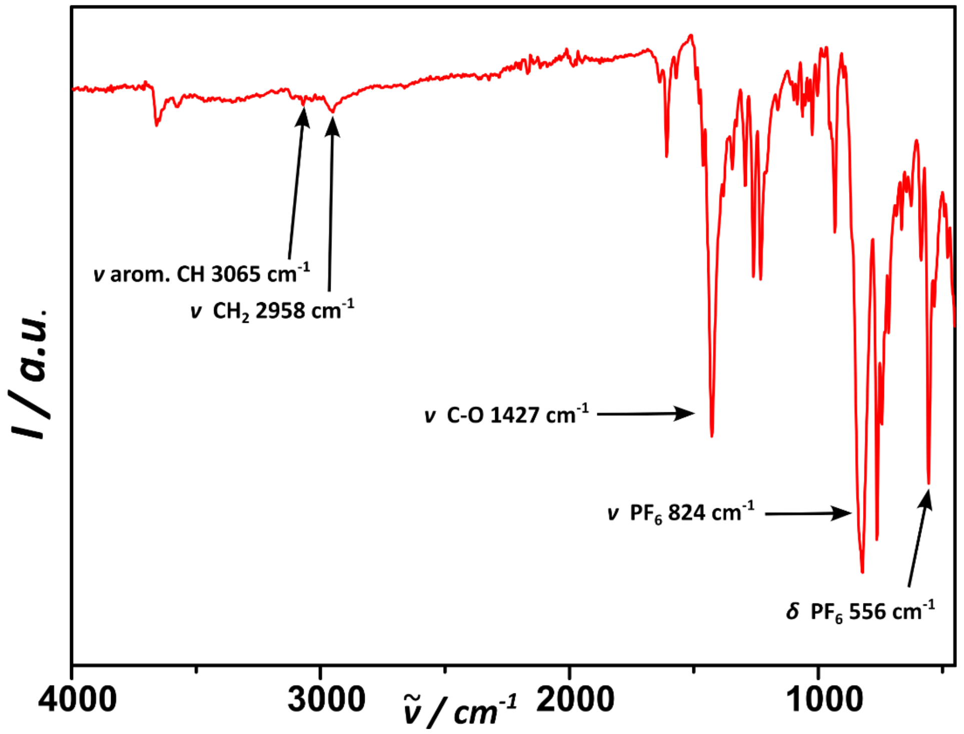

Figure S10: ATR-IR of $[Co_2(\mathbf{L})(Br_4\text{-}cat)_2]\ (PF_6)_2 \cdot 1.5\ H_2O$ (**C7**· 1.25 H_2O) at room temperature.

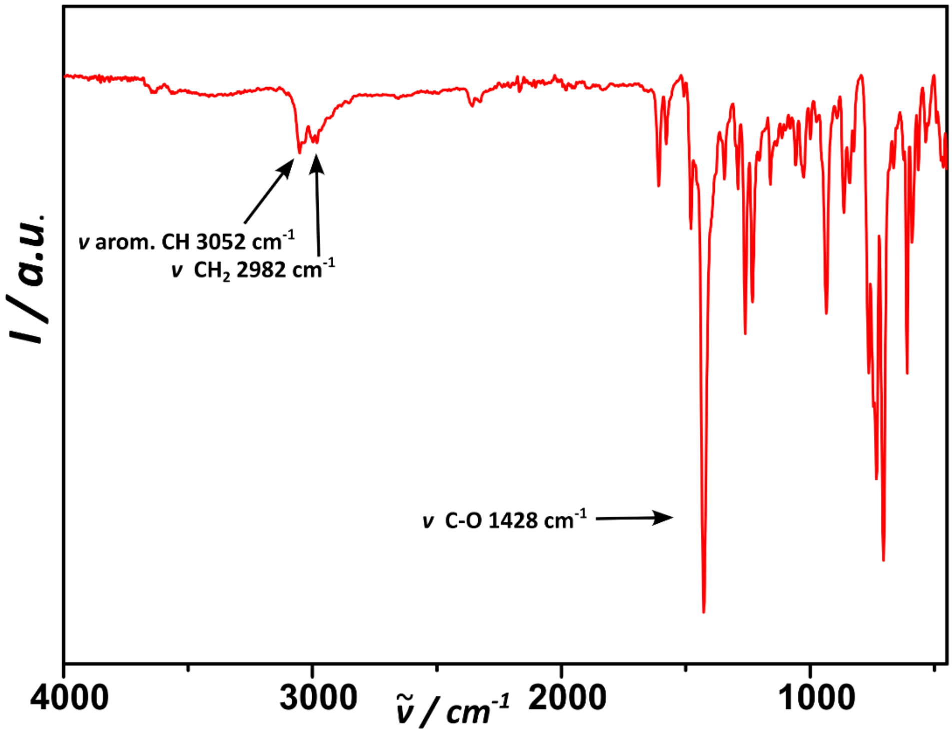

Figure S11: ATR-IR of $[Co_2(\mathbf{L})(Br_4\text{-cat})_2](B(Ph)_4)_2 \cdot 1\ H_2O$ (**C8**$\cdot$ $1H_2O$) at room temperature.

2. ^{1}H-NMR

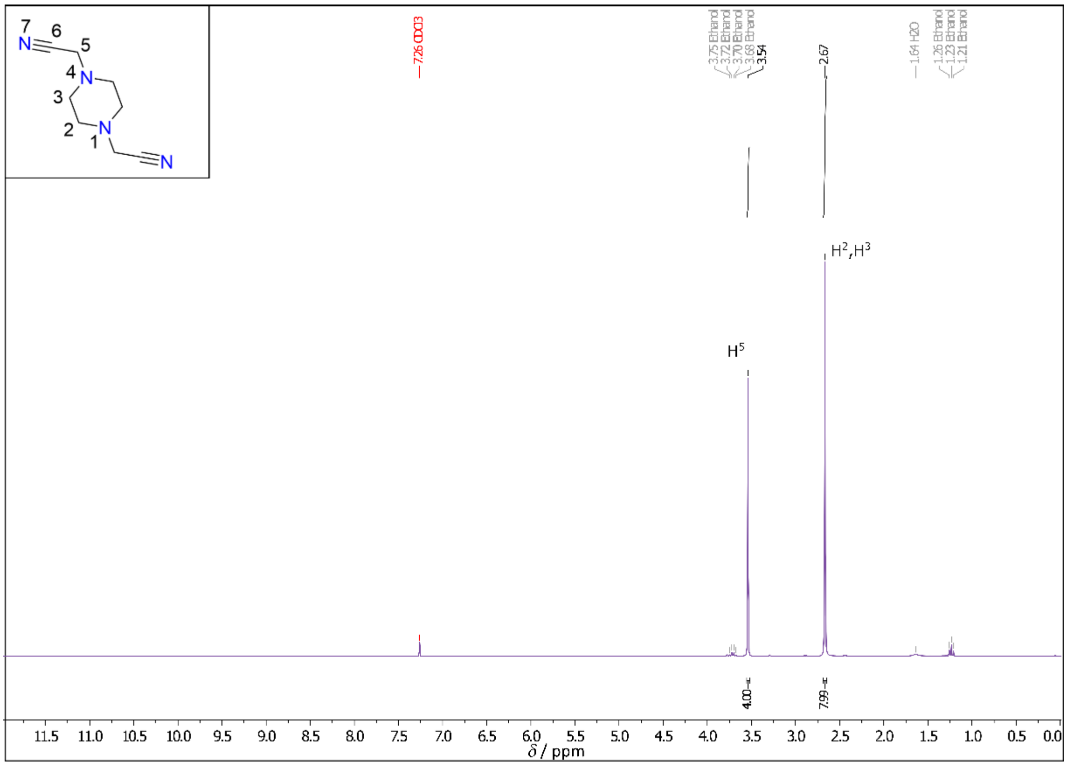

Figure S12: ^{1}H-NMR-Spectrum of *N*, *N*'-bis(cyanomethyl)piperazine in $CDCl_3$ [400 MHz].

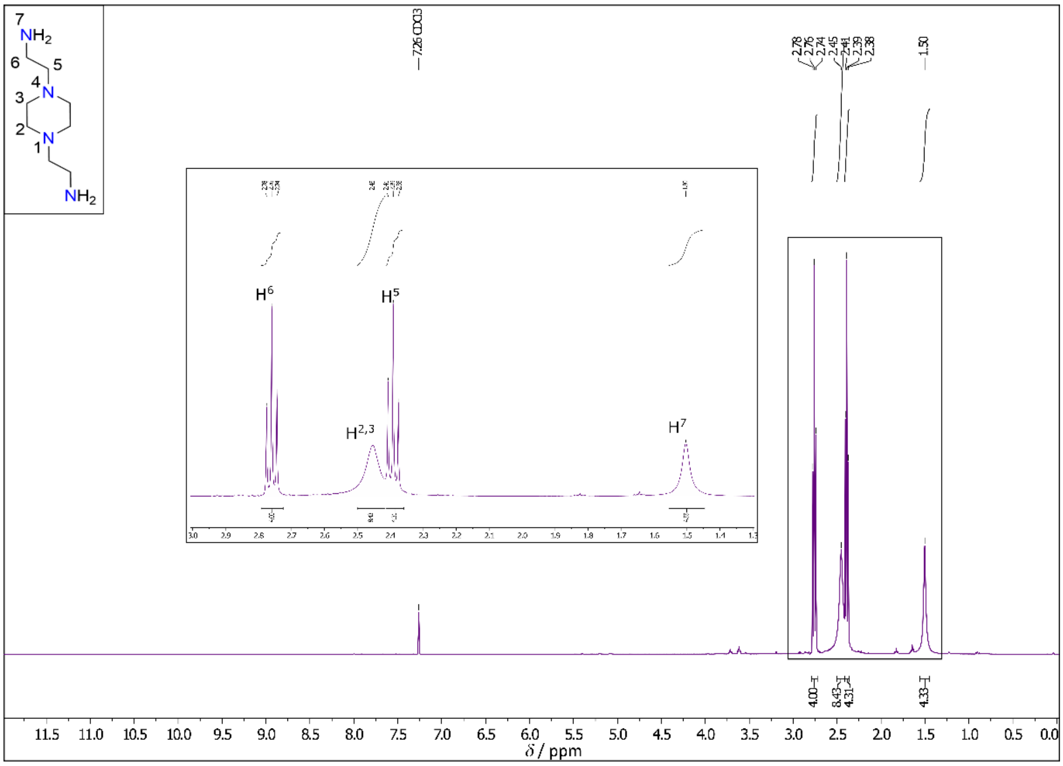

Figure S13: ^{1}H-NMR-Spectrum of *N*, *N*'-bis(aminoethyl)piperazine in $CDCl_3$ [400 MHz].

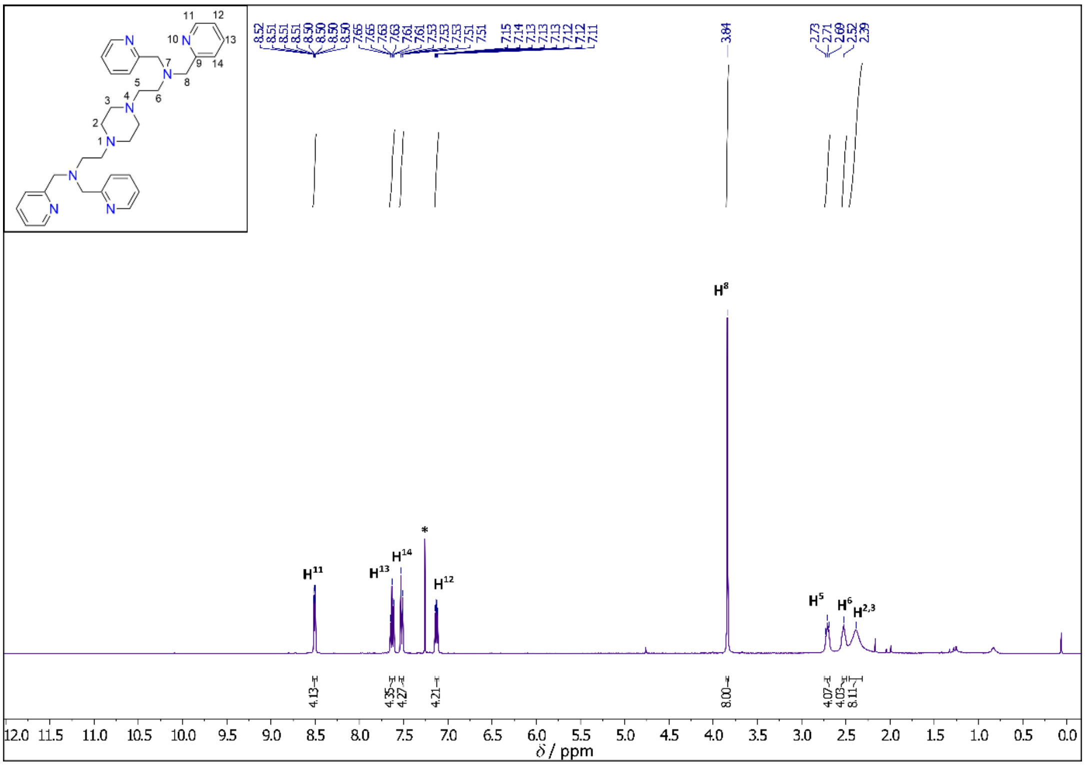

Figure S14: ^{1}H-NMR of *N*, *N*, *N*', *N*'-Tetra-2-picolyl-1,4-bis(2-aminoethyl)piperazine (**L**) in $CDCl_3$ [400 MHz].

3. ^{13}C-NMR

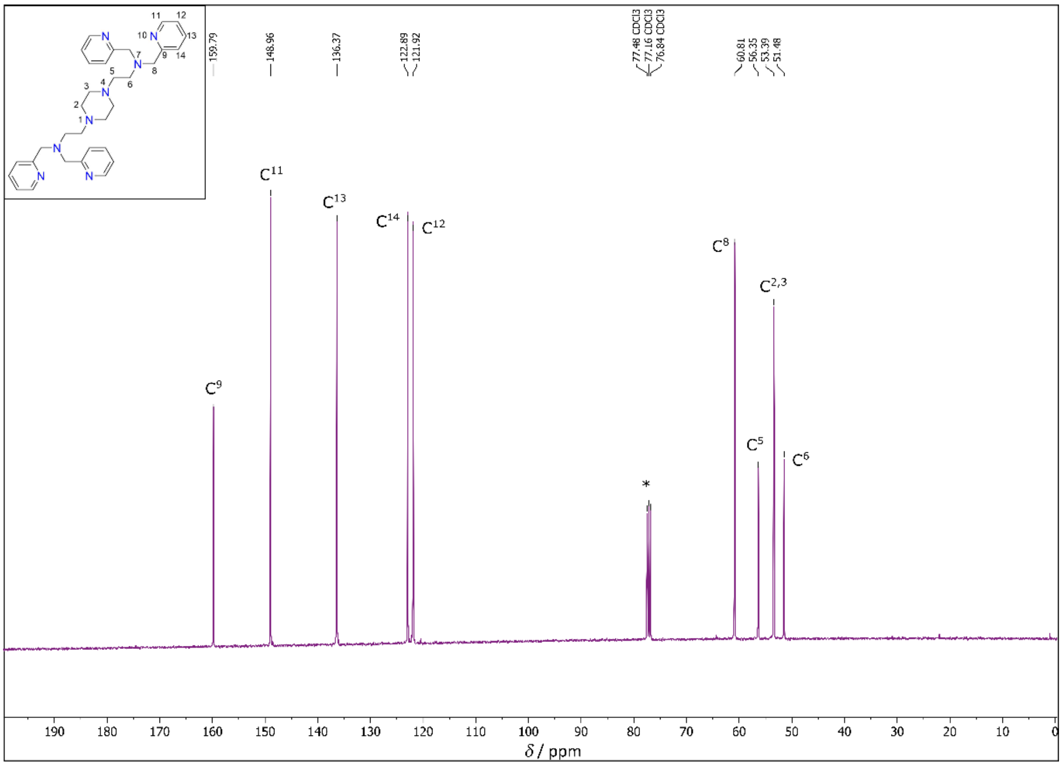

Figure S15: ^{13}C-NMR of *N*, *N*, *N*', *N*'-Tetra-2-picolyl-1,4-bis(2-aminoethyl)piperazine (**L**) in $CDCl_3$ [101 MHz].

4. COSY-NMR

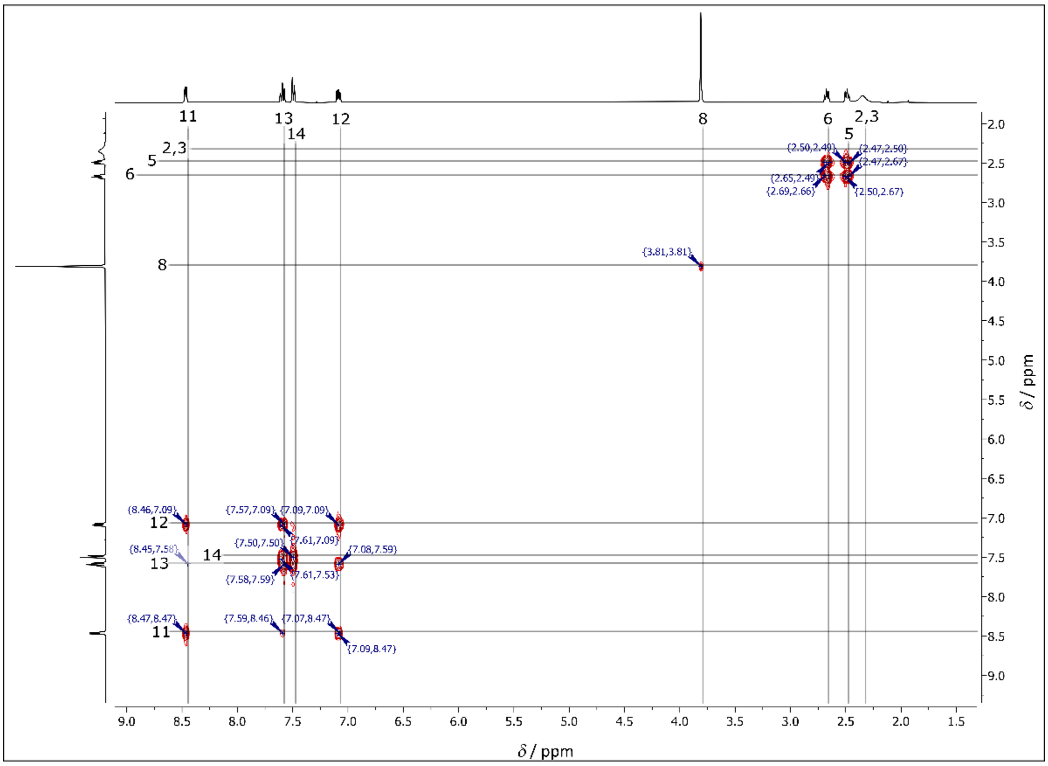

Figure S16: COSY spectrum of *N*, *N*, *N*', *N*'-Tetra-2-picolyl-1,4-bis(2-aminoethyl)piperazine (**L**) in $CDCl_3$.

5. HMBC-NMR

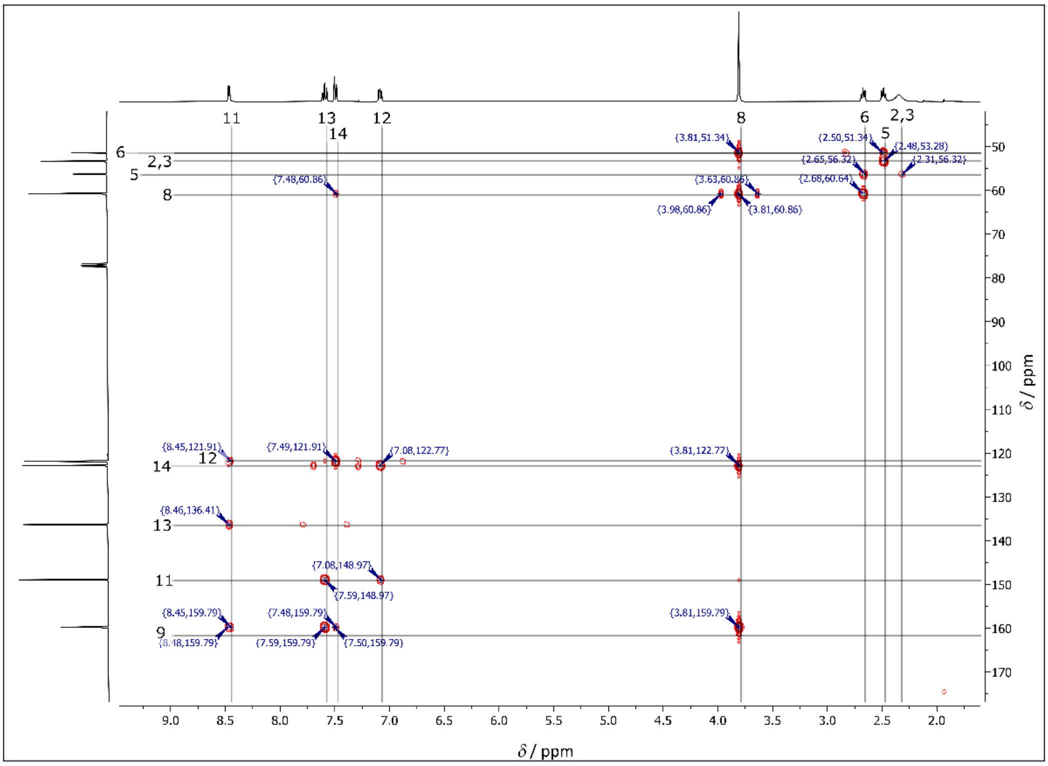

Figure S17: HMBC spectrum of *N*, *N*, *N*', *N*'-Tetra-2-picolyl-1,4-bis(2-aminoethyl)piperazine (**L**) in $CDCl_3$.

6. HSQC-NMR

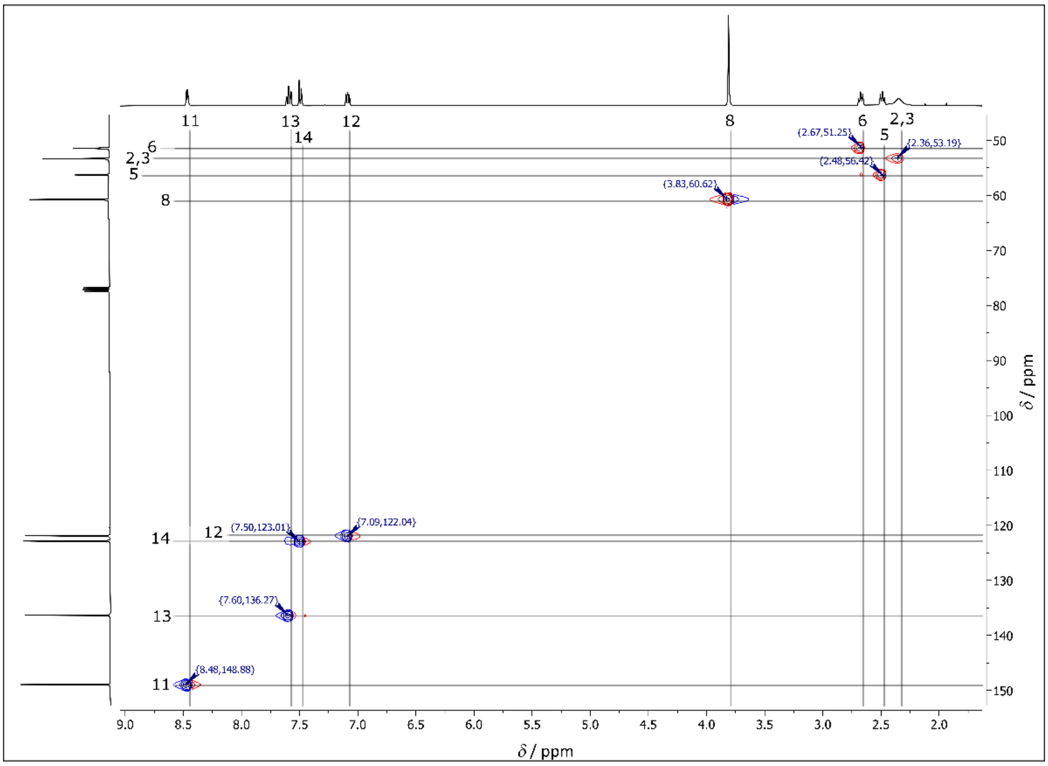

Figure S18: HSQC spectrum of *N*, *N*, *N*', *N*'-Tetra-2-picolyl-1,4-bis(2-aminoethyl)piperazine (**L**) in $CDCl_3$.

7. DOSY-^{1}H-NMR-Spectra

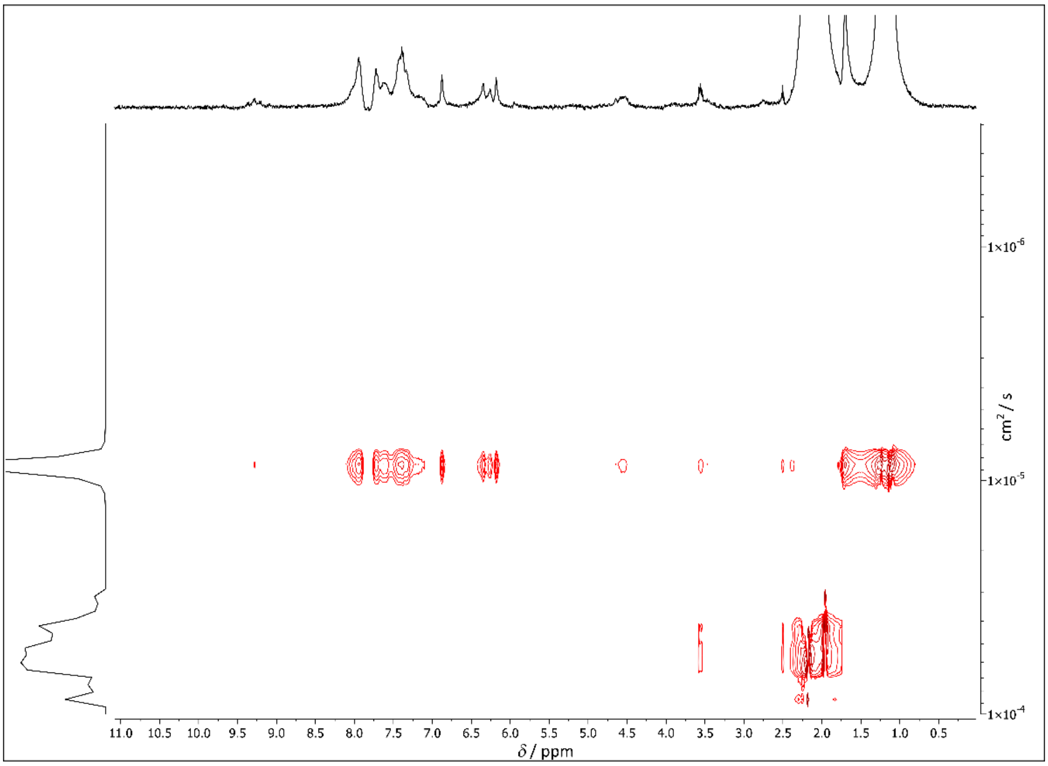

Figure S19: ^{1}H-DOSY-NMR of previously reported $[Co_2(\mathbf{L})(dbucat)_2](ClO_4)_2 \cdot 1.5\ H_2O$[1] in acetonitrile [400 MHz] (D = 7.90 10^{-6} cm^2s^{-1})

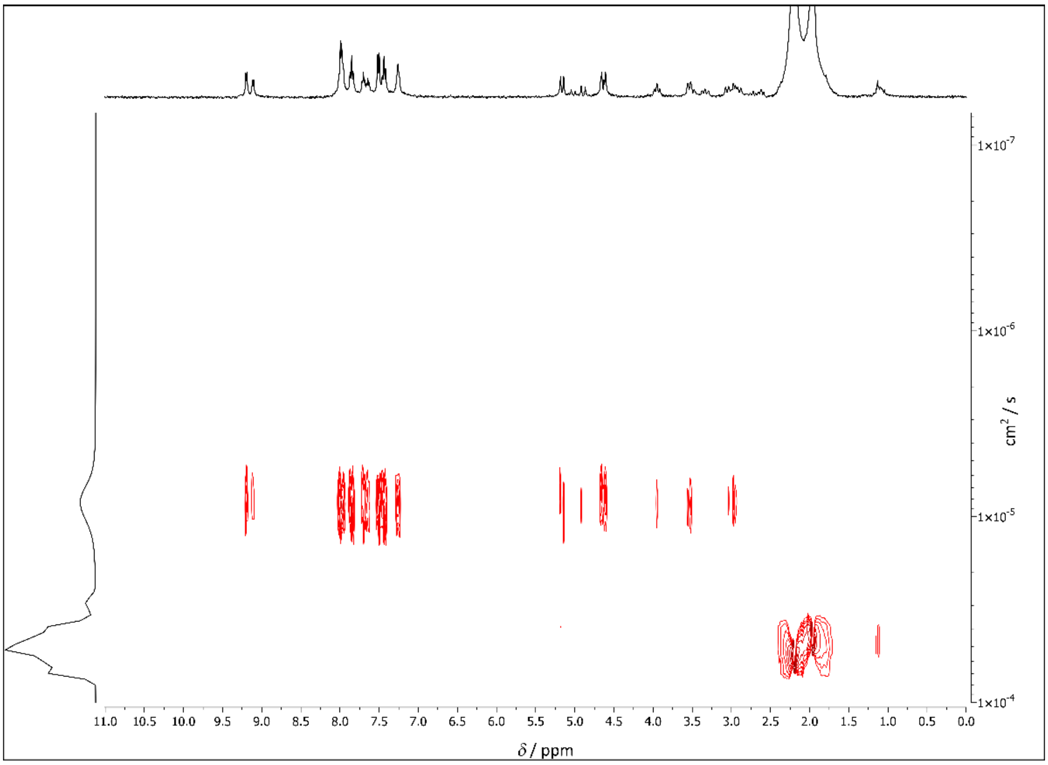

Figure S20: ^{1}H-DOSY-NMR of **C2** $\cdot$ 3.5 H_2O in acetonitrile [400 MHz] (D = 8.49 10^{-6} cm^2s^{-1}).

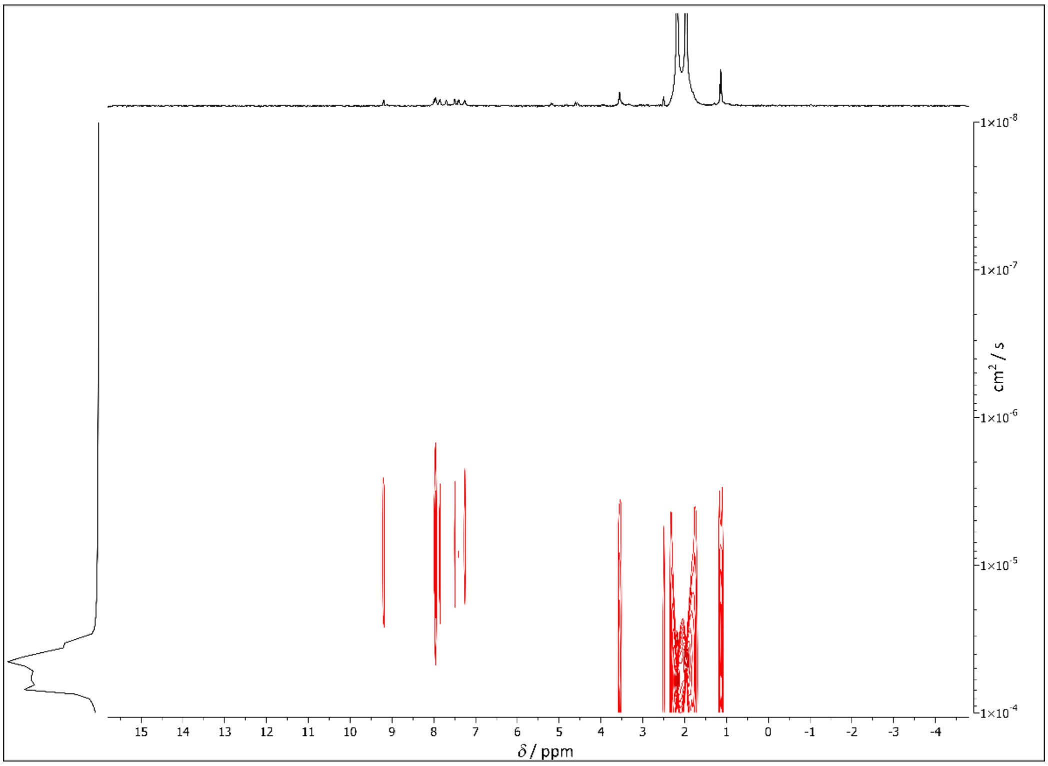

Figure S21: ^{1}H-DOSY-NMR of **C6** · 1.5 H_2O in acetonitrile [400 MHz] (D = 8.49 10^{-6} cm^2s^{-1}).

8. Complex Synthesis

Caution: While no issues were encountered during the course of this work, nonetheless, it is important to note that perchlorate salts have the potential to be explosive, so careful handling is advised.

$[Co_2$(**L**)$(Cl_4cat)_2]$ SO_4· 11 MeOH [**C1**]

To a solution of $CoSO_4 \cdot 7\ H_2O$ (56 mg, 0.2 mmol, 2.0 eq.), **L** (54 mg, 0.1 mmol, 1.0 eq.) and tetrachlorocatechol (Cl_4cat) (50 mg, 0.2 mmol, 2.0 eq.) in 15 mL methanol triethylamine (40 mg, 0.4 mmol, 4.0 eq.) was added dropwise. A suspension immediately formed, consisting of an orange precipitate in a green solution. The reaction mixture was refluxed for one hour. After the reaction mixture had cooled down to room temperature, it was filtered. After one day of slow evaporation green crystals suitable for X-ray diffraction were obtained. The product obtained was filtered, washed three times with 5 mL of ice-cold methanol and air dried. The desired complex was obtained in low yields (green plates, 33 mg, 0.038 mmol, 37.5 %). IR $\tilde{\upsilon}$ [cm^{-1}]: 3610(w), 3170(broad) 3071(w), 3011(w), 2947(w), 1664(w), 1608(w), 1433(ss), 1376(m), 1321(w), 1291(w), 1252(m), 1220(w), 1137(m), 1082(m), 1052(m), 1021(m), 1001(w), 970(m), 945(w), 866(w), 841(w), 824(w), 809(m), 798(s), 772(s), 729(m), 716(m), 693(m), 665(m), 647(w), 611(m), 592(m), 550(m), 531(m), 490(m), 473(m), 457(m), 434(m). Elemental Analysis: Found: C, 37.77%; H, 4.11%; N, 7.96%. Calc. for $C_{44}H_{40}Cl_8Co_2N_8O_8S \cdot 8\ H_2O$: C, 38.12%; H, 4.07%; N, 8.08%. Mass spectrometry [m/z]: 572.964 [**C1**]$^{2+}$ (calc. = 572.964).

$[Co_2$(**L**)$(Cl_4cat)_2](ClO_4)_2 \cdot 3\ H_2O \cdot 1$ MeCN [**C2**]

To a solution of $Co(ClO_4)_2 \cdot 6\ H_2O$ (73 mg, 0.2 mmol, 2.0 eq.), **L** (54 mg, 0.1 mmol, 1.0 eq.) and tetrachlorocatechol (Cl_4cat) (50 mg, 0.2 mmol, 2.0 eq.) in 15 mL acetonitrile triethylamine (40 mg, 0.4 mmol, 4.0 eq.) was added dropwise. A suspension immediately formed, consisting of an orange precipitate in a green solution. The reaction mixture was refluxed for one hour. After the reaction mixture had cooled down to room temperature, it was filtered. After three days of slow evaporation green crystals suitable for X-ray diffraction were obtained. The product obtained was filtered, washed three times with 5 mL of ice-cold acetonitrile and air dried. The desired complex was obtained in low yields (green plates, 30 mg, 0.022 mmol, 22.3 %). IR $\tilde{\upsilon}$ [cm^{-1}]: 3548(m), 3073(m), 2962(m), 1638(m), 1609(m), 1462(m), 1434(s), 1376(s), 1327(m), 1291(m), 1251(s), 1224(m), 1079(ss), 1039(s), 1023(s), 1002(m), 972(s), 954(s), 931(m), 893(m), 865(s), 839(m), 827(m), 810(s), 798(s), 766(s), 731(m), 716(m), 693(m), 665(m), 646(m), 623(s), 595(s), 556(m), 530(m), 488(s), 472(m), 455(s), 434(s). Elemental Analysis: Found: C, 37.33%; H, 3.15%; N, 8.27%. Calc. for $C_{44}H_{40}Cl_{10}Co_2N_8O_{12} \cdot 3.5\ H_2O$, C, 37.53%; H, 3.36%; N, 7.96% Mass spectrometry [m/z]: 572.964 [**C2**]$^{2+}$ (calc. = 572.964), 1244.880 [**C2**+ClO_4]$^+$ (calc. = 1244.877).

$[Co_2$(**L**) $(Cl_4cat)_2](PF_6)_2 \cdot 2$ MeOH · 2 MeCN [**C3**]

To a solution of $CoCl_2 \cdot 6\ H_2O$ (48 mg, 0.2 mmol, 2.0 eq.), **L** (54 mg, 0.1 mmol, 1.0 eq.) and Cl_4cat (50 mg, 0.2 mmol, 2.0 eq.) in 15 mL methanol triethylamine diluted in 1 mL of methanol (40 mg, 0.4 mmol, 4.0 eq.) was added dropwise. A colour change from blue to green was observed. The reaction mixture was refluxed for one hour. After the reaction mixture had cooled down to room temperature, it was filtered. KPF_6 (37 mg, 0.2 mmol, 2.0 eq.) was added

to the filtrate during stirring. It was stirred for 15 minutes. A greenish very fine suspension formed. The suspension was centrifuged at 3500 rpm for 20 minutes and filtrated. The green powder was air dried overnight. The product was obtained as a green powder in moderate yields. Suitable single crystals for single crystal XRD measurements were obtained after repeating the procedure in a 1:1 mixture of 20 mL MeOH:MeCN and slow evaporation of the mother liquor. (green powder 68 mg, 0.047 mmol, 47.3%). IR $\tilde{\upsilon}$ [cm^{-1}]: 2954(w), 1639(w), 1610(w), 1435(s), 1377(w), 1293(w), 1251(m), 1025(w), 975(m), 955(w), 838(ss), 811(s), 800(m), 766(m), 717(m), 694(w), 666(w), 597(m), 558(s), 456(m), 433(m). Elemental Analysis: Found: C, 34.19%; H, 3.72%; N, 7.34% Calc. for $C_{44}H_{40}Cl_8Co_2F_{12}N_8O_4P_2 \cdot 6.5\ H_2O$ C, 34.02%; H, 3.44%; N, 7.21% Mass spectrometry [m/z]: 572.964 [**C3**]$^{2+}$ (calc. = 572.964).

$[Co_2$**(L)**$(Cl_4cat)_2](B(Ph)_4)_2 \cdot 1$ MeCN [**C4**]

To a solution of $CoCl_2 \cdot 6\ H_2O$ (48 mg, 0.2 mmol, 2.0 eq.), **L** (54 mg, 0.1 mmol, 1.0 eq.) and Cl_4cat (50 mg, 0.2 mmol, 2.0 eq.) in 15 mL methanol triethylamine diluted in 1 mL of methanol (40 mg, 0.4 mmol, 4.0 eq.) was added dropwise. A colour change from blue to green was observed. The reaction mixture was refluxed for one hour. After the reaction mixture had cooled down to room temperature, it was filtered. The solution was filtrated. $NaB(Ph)_4$ (69 mg, 0.2 mmol, 2.0 eq.) was added to the filtrate during stirring. It was stirred for 15 minutes. A greenish very fine suspension formed. The suspension was centrifuged at 3500 rpm for 20 minutes and filtrated. The green powder was air dried overnight. The product was obtained as a green powder in low to moderate yields. The product was obtained as a green powder in moderate yields. Suitable single crystals for single crystal XRD measurements were obtained after repeating the procedure in a 1:1 mixture of 20 mL MeOH:MeCN and slow evaporation of the mother liquor. (green powder 56 mg, 0.031 mmol, 31.4%). IR $\tilde{\upsilon}$ [cm^{-1}]: 3052(w), 1609(m), 1579(w), 1478(m), 1435(s), 1376(m), 1290(m), 1251(m), 1160(m), 1058(w), 1025(m), 975(m), 943(w), 866(m), 812(m), 800(m), 767(m), 734(s), 705(s), 665(w), 612(m), 598(m), 562(w), 470(w), 434(m). Elemental Analysis: Found: C, 60.34%; H, 4.74%; N, 6.43% Calc. for $C_{92}H_{82}B_2Cl_8Co_2N_8O_4 \cdot 2\ H_2O$: C, 60.62%; H, 4.76%; N, 6.15% Mass spectrometry [m/z]: 572.964 [**C4**]$^{2+}$ (calc. = 572.964).

$[Co_2($**L**$)(Br_4cat)_2]SO_4 \cdot 2\ H_2O \cdot 4$ MeCN [**C5**]

To a solution of $CoSO_4 \cdot 7\ H_2O$ (56 mg, 0.2 mmol, 2.0 eq.), (**L**) (54 mg, 0.1 mmol, 1.0 eq.) and Br_4cat (85 mg, 0.2 mmol, 2.0 eq.) in 20 mL of methanol/chloroform (1:1) triethylamine diluted in 1 mL of methanol (40 mg, 0.4 mmol, 4.0 eq.) was added dropwise. A suspension immediately formed, consisting of an orange precipitate in a green solution. The reaction mixture was refluxed for one hour. After the reaction mixture had cooled down to room temperature, it was filtered. After ten days of slow evaporation green crystals suitable for Xray diffraction were obtained. The product obtained was filtered, washed three times with 5 mL of ice-cold methanol and air dried. The desired complex was obtained in low yields. (green needles 19 mg, 0.012 mmol, 12.0%). IR $\tilde{\upsilon}$ [cm^{-1}]: 3606(m), 3090 (broad), 3065(m), 3007(m), 2958(s), 1650(m), 1607(m), 1459(m), 1425(ss), 1348(s), 1320(m), 1290(m), 1264(s), 1235(s), 1207(s), 1138(s), 1082(s), 1051(s), 1037(s), 1020(s), 998(s), 968(m), 956(s), 930(s), 865(s), 841(m), 823(s), 767(ss), 743(s), 728(s), 715(s), 681(s), 665(s), 647(s), 608(s), 580(s), 563(s), 534(s), 519(s), 497(s), 477(s), 460(s), 453(s), 429(s). Elemental Analysis: Found: C, 29.67%; H,

3.17%; N, 6.36%, Calc. for $C_{44}H_{40}Br_8Co_2N_8O_8S \cdot 5.5\ H_2O \cdot 1\ CHCl_3$: C, 29.76%; H, 2.89%; N, 6.17%. Mass spectrometry [m/z]: 750.761 [**C5**]$^{2+}$ (calc. = 750.761)

$[Co_2$(**L**)$(Br_4cat)_2](ClO_4)_2 \cdot 2$ MeCN [**C6**]

To a solution of $Co(ClO_4)_2 \cdot 6\ H_2O$ (73 mg, 0.2 mmol, 2.0 eq.), **L** (54 mg, 0.1 mmol, 1.0 eq.) and Br_4cat (85 mg, 0.2 mmol, 2.0 eq.) in 15 mL acetonitrile triethylamine (40 mg, 0.4 mmol, 4.0 eq.) diluted by 1 mL of acetonitrile was added dropwise. A suspension immediately formed, consisting of an orange precipitate in a green solution. The reaction mixture was refluxed for one hour. After the reaction mixture had cooled down to room temperature, it was filtered. After three days of slow evaporation green crystals suitable for X-ray diffraction were obtained. The product obtained was filtered, washed three times with 5 mL of ice-cold acetonitrile and air dried. The desired complex was obtained in low yields (green needles 19 mg, 0.011 mmol, 11.2%). IR $\tilde{\upsilon}$ [cm^{-1}]: 3617(m), 3542(m), 2940(m), 1632(m), 1608(m), 1462(m), 1428(s), 1378(m), 1346(m), 1326(m), 1292(m), 1261(s), 1233(s), 1211(m), 1079(ss), 1038(s), 1022(s), 1001(s), 979(m), 954(s), 930(s), 893(s), 864(s), 839(m), 826(s), 765(ss), 745(s), 730(s), 715(s), 686(m), 665(m), 647(m), 623(s), 589(s), 566(s), 533(s), 492(s), 476(s), 460(s), 451(s), 429(s). Elemental Analysis: Found: C, 30.22%; H, 2.49%; N, 6.47%, Calc. for $C_{44}H_{40}Br_8Cl_2Co_2N_8O_{12} \cdot 1.5\ H_2O$: C, 30.59%; H, 2.51%; N, 6.49%. Mass spectrometry [m/z]: 750.762 [**C6**]$^{2+}$ (calc. = 750.761), 1600.470 [**C6**+ClO_4]$^{+}$ (calc. = 1600.470).

$[Co_2$(**L**)$(Br_4cat)_2](PF_6)_2 \cdot 1$ MeOH $\cdot 1$ MeCN [**C7**]

To a solution of $CoCl_2 \cdot 6\ H_2O$ (48 mg, 0.2 mmol, 2.0 eq.), **L** (54 mg, 0.1 mmol, 1.0 eq.) and Br_4cat (85 mg, 0.2 mmol, 2.0 eq.) in 20 mL of methanol : acetonitrile (1:1) triethylamine (40 mg, 0.4 mmol, 4.0 eq.) diluted by 1 mL of acetonitrile was added dropwise. The reaction mixture was refluxed for one hour and afterwards filtrated. KPF_6 (37 mg, 0.2 mmol, 2.0 eq.) was added to the filtrate during stirring. It was stirred for 15 minutes. Single crystals suitable for single crystal XRD measurements were obtained after slow evaporation for two days. The crystals were filtrated and air dried. The product was obtained in moderate yield as green crystals. (green plates, 56 mg, 0.031 mmol, 31.3 %). IR $\tilde{\upsilon}$ [cm^{-1}]: 3658(w), 3065 (w), 2958 (w), 1637(w), 1609(m), 1571(w), 1462(m), 1427(s), 1346(m), 1293(m), 1260(m), 1231(m), 1163(w), 1098(w), 1084(w), 1063(w), 1052(w), 1038(w), 1024(m), 1003(w), 933(m), 823(s), 764(s), 745(s), 716(m), 665(m), 626(m), 587(m), 556(s), 534(m), 479(m), 452(m), 429(m). Elemental Analysis: Found: C, 29.50%; H, 2.75%; N, 6.35%, Calc. for $C_{44}H_{40}Br_8Co_2F_{12}N_8O_4P_2 \cdot 1.25\ H_2O$: C, 29.13%; H, 2.36%; N, 6.18%. Mass spectrometry [m/z]: 750.762 [**C7**]$^{2+}$ (calc. = 750.761).

$[Co_2$(**L**)$(Br_4cat)_2]$ $(B(Ph)_4)_2 \cdot 4$ MeCN [**C8**]

To a solution of $CoCl_2 \cdot 6\ H_2O$ (48 mg, 0.2 mmol, 2.0 eq.), **L** (54 mg, 0.1 mmol, 1.0 eq.) and Br_4cat (85 mg, 0.2 mmol, 2.0 eq.) in 20 mL of methanol : acetonitrile (1:1) triethylamine (40 mg, 0.4 mmol, 4.0 eq.) diluted by 1 mL of acetonitrile was added dropwise. The reaction mixture was refluxed for one hour and afterwards filtrated. The solution was filtrated. $NaB(Ph)_4$ (69 mg, 0.2 mmol, 2.0 eq.) was added to the filtrate during stirring. It was stirred for 15 minutes. A greenish very fine suspension formed. The suspension was centrifuged at 3500 rpm for 20 minutes and filtrated. The green powder was air dried overnight. The product was obtained as

a green powder in high yields. Suitable single crystals for single crystal XRD measurements were obtained from slow evaporation of the filtrated mother liquor after 1 day. (green powder, 190 mg, 0.089 mmol, 88.7 %). IR $\tilde{\upsilon}$ [cm^{-1}]: 3052(w), 2998(w), 2982(w), 1608(w), 1578(w), 1479(m), 1428(ss), 1345(w), 1290(w), 1261(m), 1232(m), 1160(w), 1058(w), 1027(w), 999(w), 935(m), 865(w), 842(w), 765(s), 733(s), 705(ss), 665(w), 611(s), 591(m), 565(w), 536(w), 465(w), 451(w), 429(w). Elemental Analysis: Found: C, 50.98%; H, 3.97%; N, 5.43%, Calc. for $C_{92}H_{82}Br_8Co_2N_8O_4 \cdot 1\ H_2O$: C, 51.15%; H, 3.92%; N, 5.19%. Mass spectrometry [m/z]: 750.761 [**C8**]$^{2+}$ (calc. = 750.761).

[Co_2(**L**)(dbucat)$_2$] $(ClO_4)_2 \cdot 1.5\ H_2O$ [**C9**]

Was prepared as previously reported. (1)

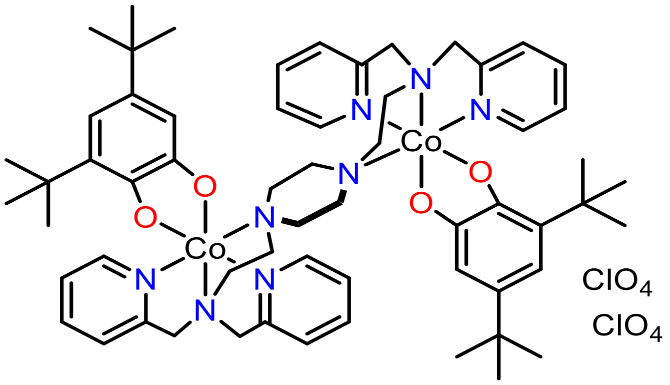

Scheme S1: Structural Scheme of **C9.**

9. Mass Spectrometry

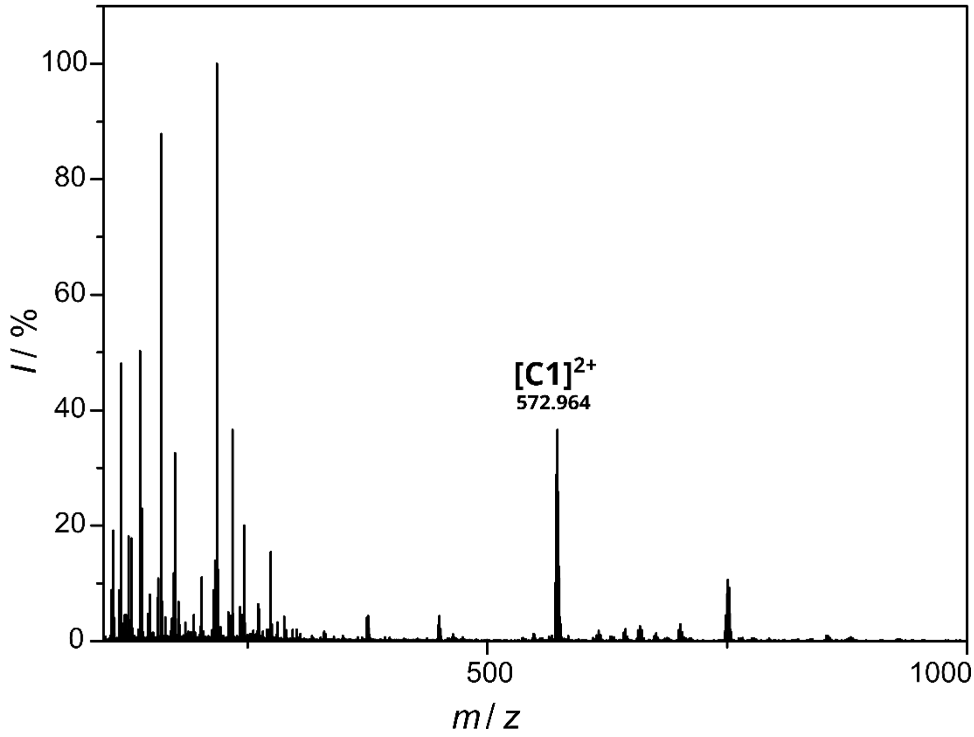

Figure S22: ESI$^+$-MS of **C1** ·8 H_2O showing the dicationic [**C1**]$^{2+}$ fragment without anions at 572.964 *m*/z.

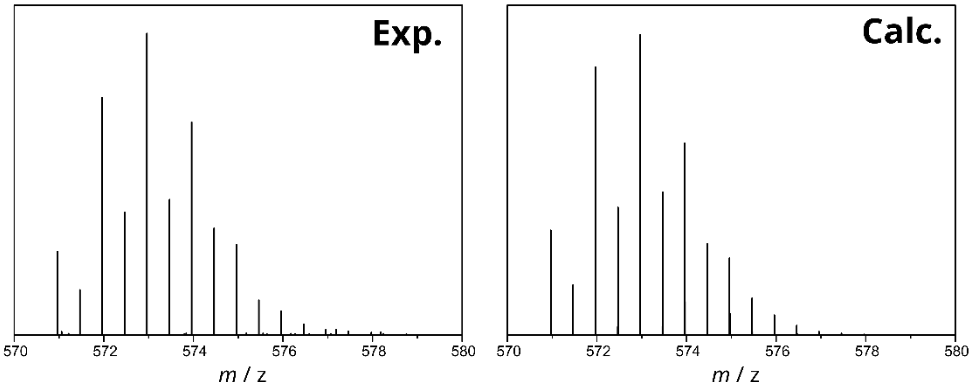

Figure S23: Comparison of the experimentally measured isotopic pattern of [**C1**]$^{2+}$ at 572.964 *m*/z with the theoretically calculated isotopic pattern of [**C1**]$^{2+}$.

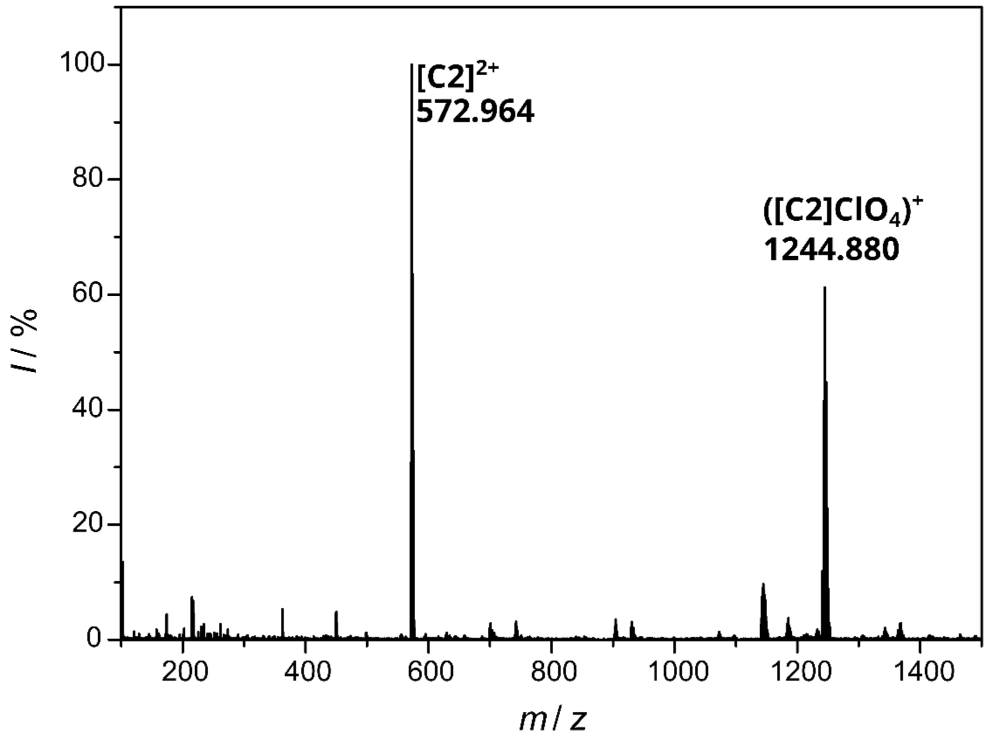

Figure S24: ESI$^+$-MS of **C2** ·3.5 H_2O showing the dicationic [**C2**]$^{2+}$ fragment without anions at 572.964 *m*/z as well as the monocationic ([**C2**]ClO_4)$^+$ signal at 1244.880 m/z.

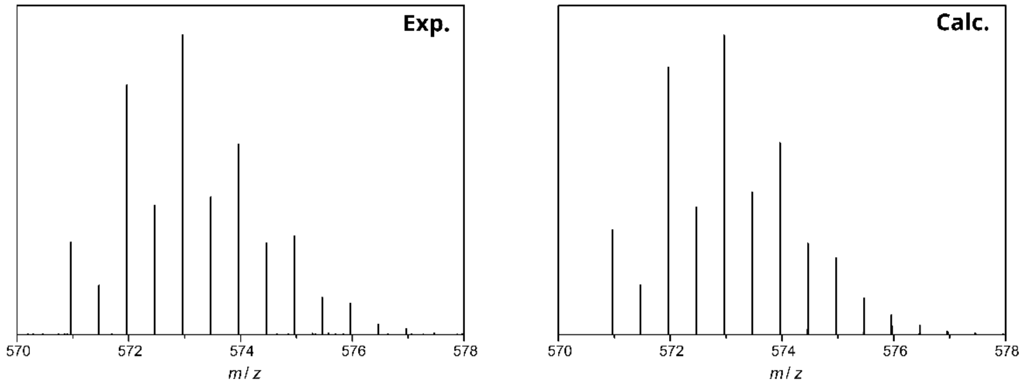

Figure S25: Comparison of the experimentally measured isotopic pattern of [**C2**]$^{2+}$ at 572.964 *m*/z with the theoretically calculated isotopic pattern of [**C2**]$^{2+}$.

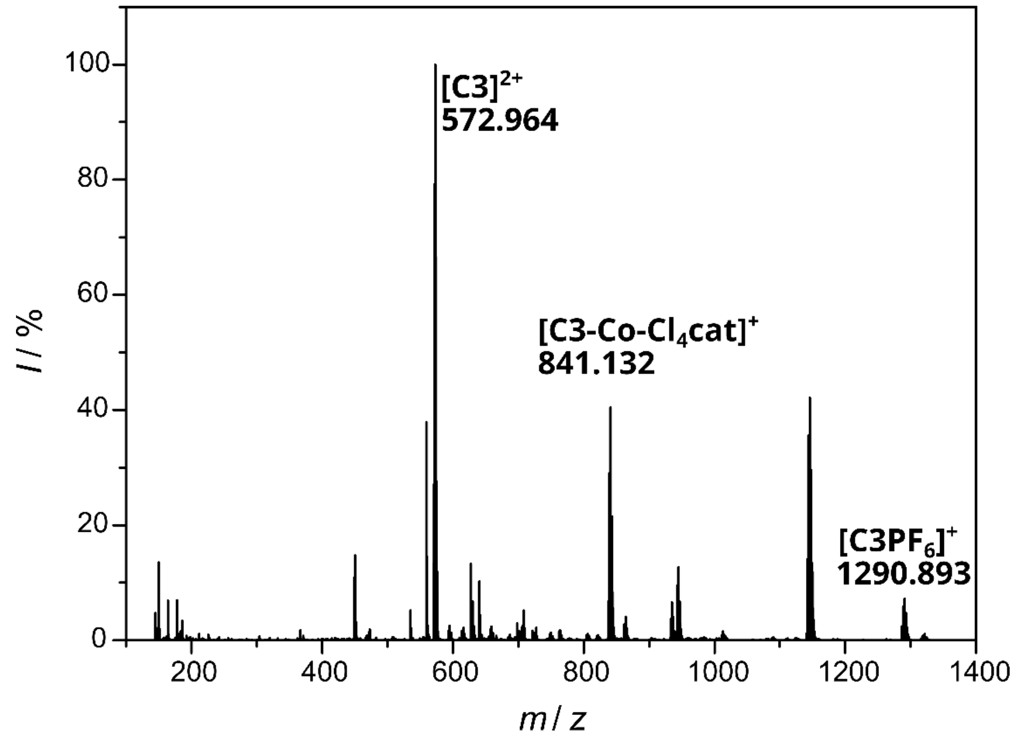

Figure S26: ESI$^+$-MS of **C3** ·6.5 H_2O showing the dicationic [**C3**]$^{2+}$ fragment without anions at 572.964 *m*/z as well as the monocationic ([**C3**]-Co-Cl_4cat)$^+$ signal at 841.132 m/z and the monocationic ([**C3**]PF_6)$^+$ at 1290.893 m/z.

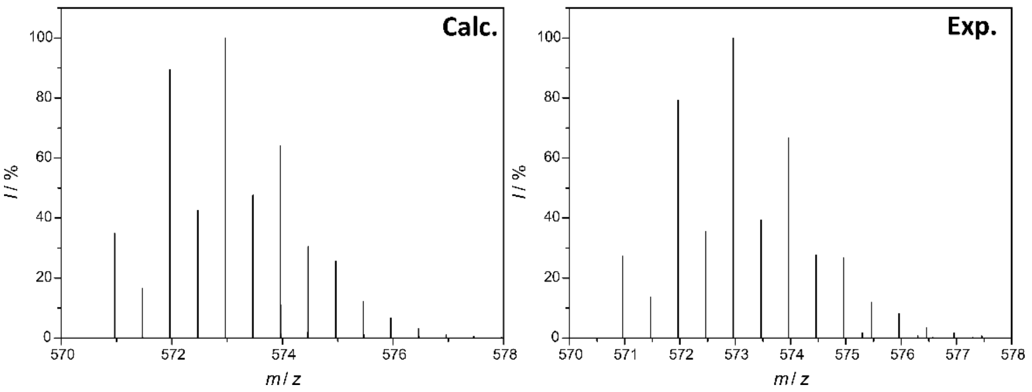

Figure S27: Comparison of the experimentally measured isotopic pattern of [**C3**]$^{2+}$ at 572.964 *m*/z with the theoretically calculated isotopic pattern of [**C3**]$^{2+}$.

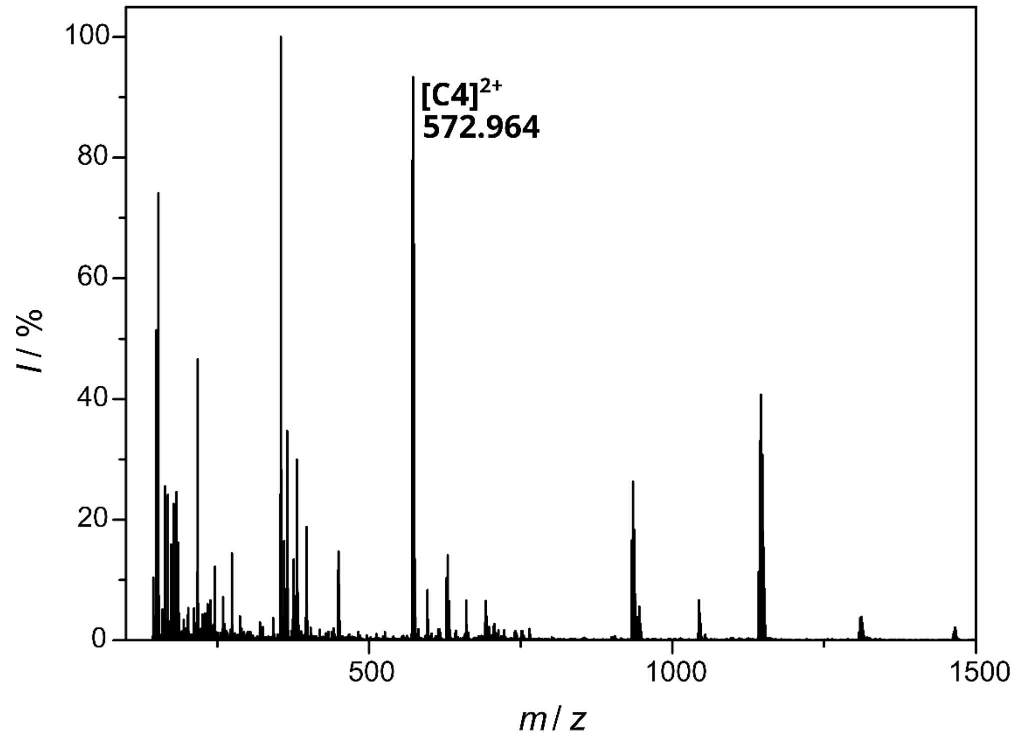

Figure S28: ESI$^+$-MS of **C4** ·2 H_2O showing the dicationic [**C4**]$^{2+}$ fragment without anions at 572.964 *m*/z.

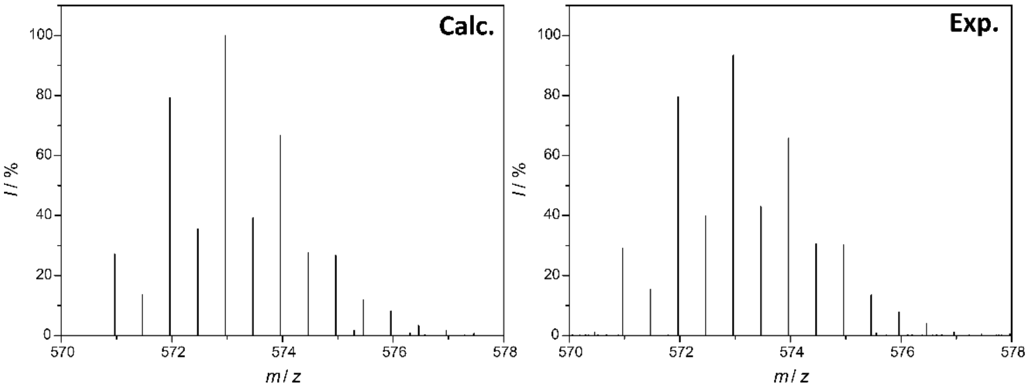

Figure S29: Comparison of the experimentally measured isotopic pattern of [**C4**]$^{2+}$ at 572.964 *m*/z with the theoretically calculated isotopic pattern of [**C4**]$^{2+}$.

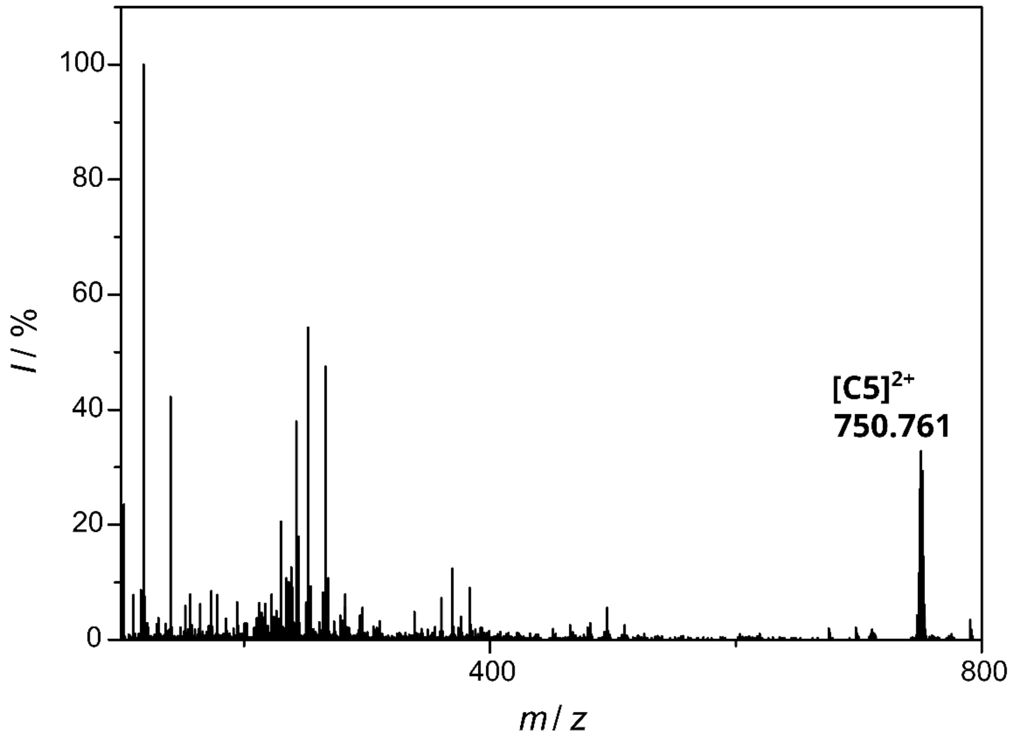

Figure S30: ESI^{+}-MS of **C5** · 5.5 H_2O · $CHCl_3$ showing the dicationic [**C5**]$^{2+}$ fragment without anions at 750.761 *m*/z.

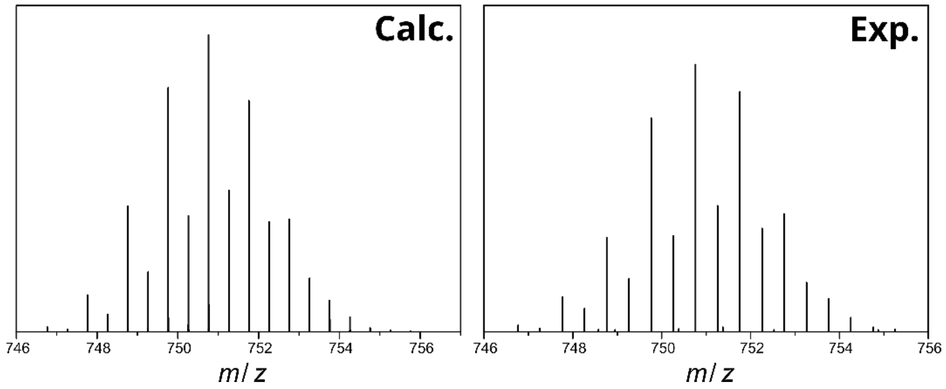

Figure S31: Comparison of the experimentally measured isotopic pattern of [**C5**]$^{2+}$ at 750.761 *m*/z with the theoretically calculated isotopic pattern of [**C5**]$^{2+}$.

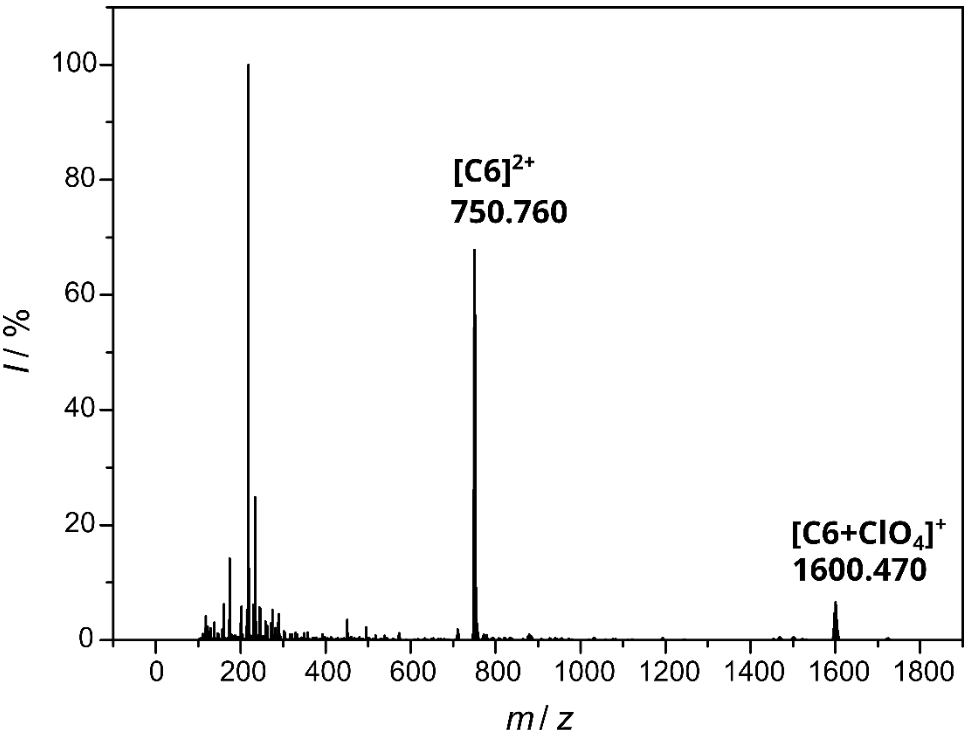

Figure S32: ESI^{+}-MS of **C6** ·1.5 H_2O showing the dicationic [**C6**]$^{2+}$ fragment without anions at 750.761 *m*/z as well as the monocationic ([**C6**]ClO_4)$^{+}$ signal at 1600.470 m/z.

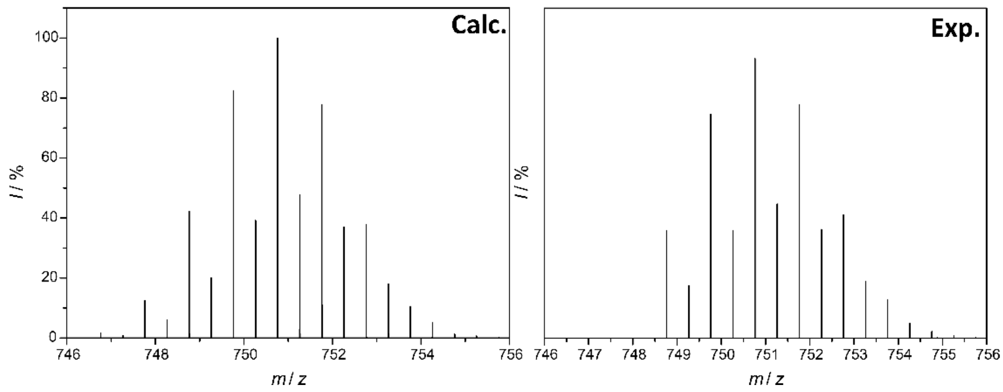

Figure S33: Comparison of the experimentally measured isotopic pattern of [**C6**]$^{2+}$ at 750.760 *m*/z with the theoretically calculated isotopic pattern of [**C6**]$^{2+}$.

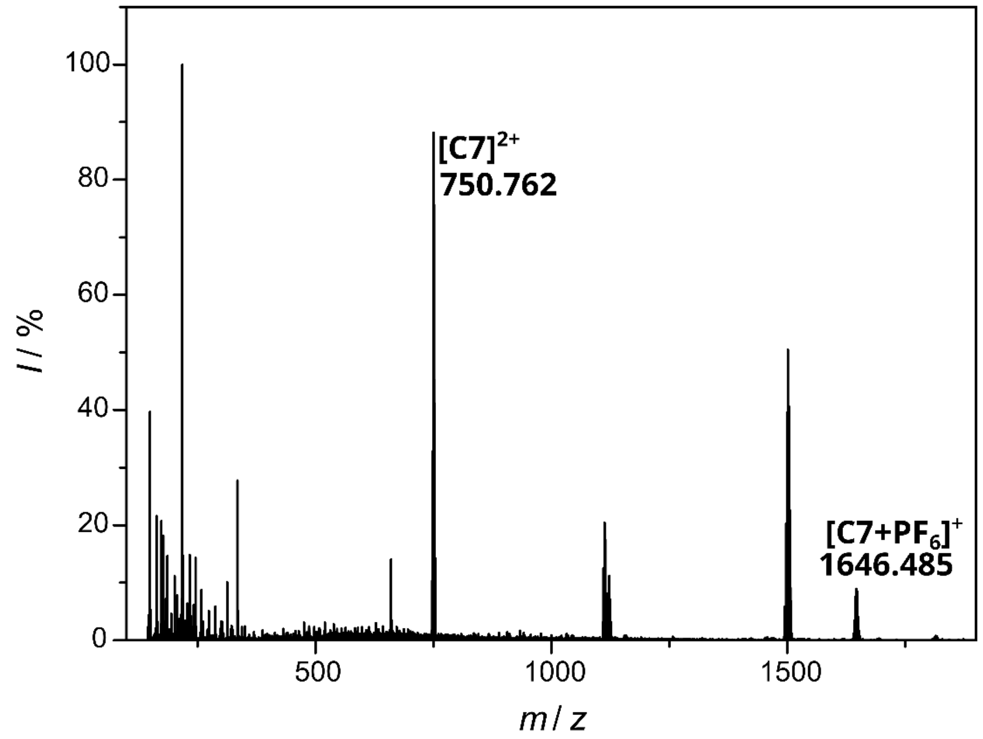

Figure S34: ESI$^+$-MS of **C7** ·1.25 H_2O showing the dicationic [**C7**]$^{2+}$ fragment without anions at 750.762 *m*/z as well as the monocationic ([**C7**]PF_6)$^+$ signal at 1646.485 m/z.

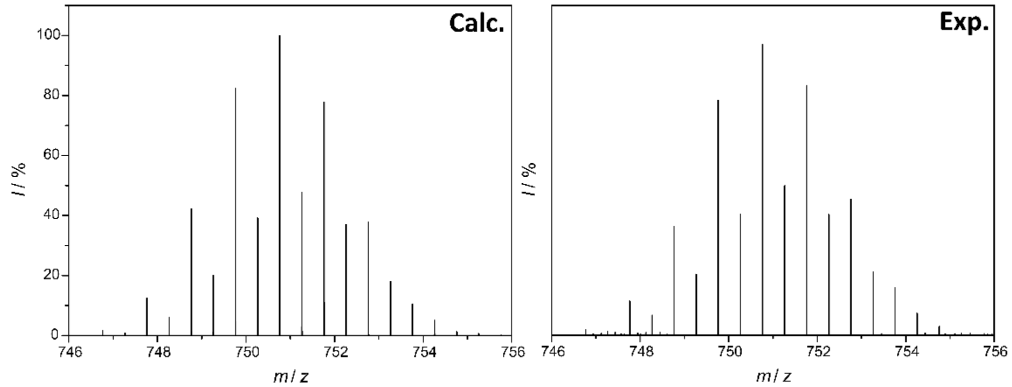

Figure S35: Comparison of the experimentally measured isotopic pattern of [**C7**]$^{2+}$ at 750.762 *m*/z with the theoretically calculated isotopic pattern of [**C7**]$^{2+}$.

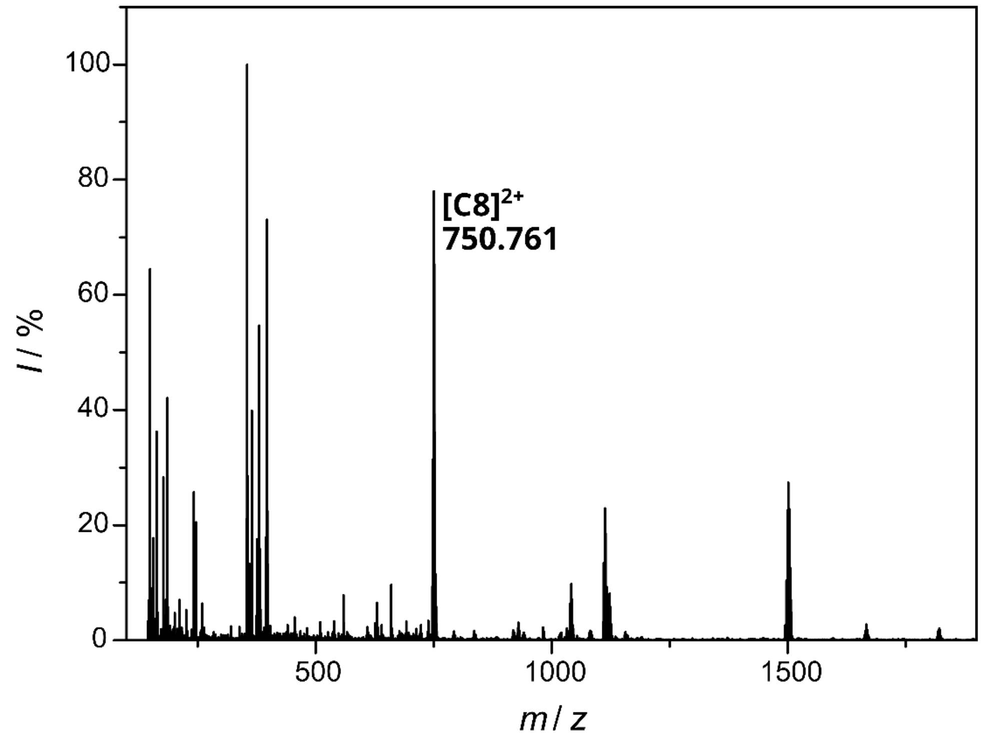

Figure S36: ESI+-MS of **C8** ·1 H_2O showing the dicationic [**C8**]$^{2+}$ fragment without anions at 750.761 *m*/z.

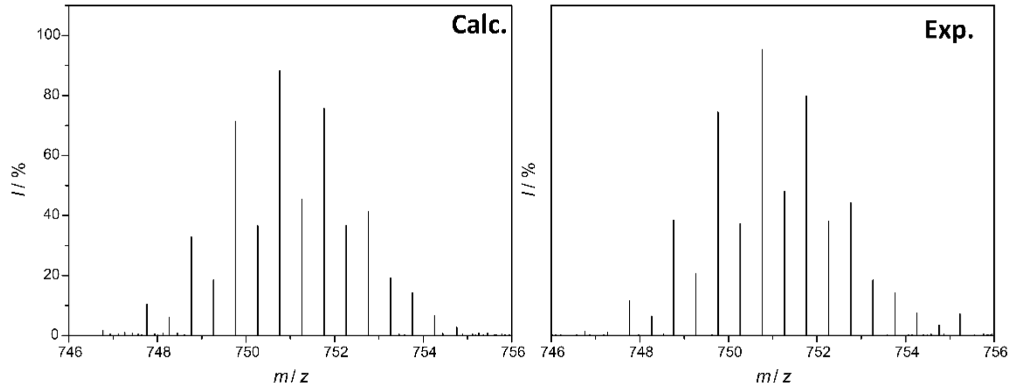

Figure S37: Comparison of the experimentally measured isotopic pattern of [**C8**]$^{2+}$ at 750.761 *m*/z with the theoretically calculated isotopic pattern of [**C8**]$^{2+}$.

10. Crystallographic Data

Table S1: Crystallographic Data of complexes **C1** and **C2.**

	**C1**	**C2**
**Empirical formula**	$C_{55}H_{84}Cl_8Co_2N_8O_{19}S$	$C_{92}H_{98}Cl_{20}Co_4N_{18}O_{30}$
**Formula weight**	1594.82	2880.60
**Temperature/K**	120.0	120
**Crystal system**	monoclinic	triclinic
**Space group**	$P2_1/c$	P-1
**a/Å**	12.8328(5)	9.9351(3)
**b/Å**	29.4246(14)	11.4887(4)
**c/Å**	19.0165(7)	26.4686(8)
**α/°**	90	95.276(3)
**β/°**	93.999(3)	94.497(3)
**γ/°**	90	97.847(3)
**Volume/Å^3**	7163.1(5)	2967.50(17)
**Z**	4	1
**ρ_{calc}g/cm^3**	1.479	1.612
**μ/mm^{-1}**	0.862	1.080
**F(000)**	3312.0	1464.0
**Crystal size/mm^3**	0.242 × 0.117 × 0.033	0.31 × 0.14 × 0.01
**Radiation**	MoKα (λ = 0.71073)	MoKα (λ = 0.71073)
**2Θ range for data collection/°**	4.196 to 49.998	4.064 to 59.996
**Index ranges**	-14 ≤ h ≤ 15, -32 ≤ k ≤ 34, -22 ≤ l ≤ 22	-13 ≤ h ≤ 13, -16 ≤ k ≤ 16, -37 ≤ l ≤ 37
**Reflections collected**	37682	72270
**Independent reflections**	12512 [R_{int} = 0.0922, R_{sigma} = 0.0794]	17255 [R_{int} = 0.0517, R_{sigma} = 0.0390]
**Data/restraints/parameters**	12512/0/858	17255/66/789
**Goodness-of-fit on F^2**	1.096	1.104
**Final R indexes [I>=2σ (I)]**	R_1 = 0.1368, wR_2 = 0.3310	R_1 = 0.1048, wR_2 = 0.2844
**Final R indexes [all data]**	R_1 = 0.1844, wR_2 = 0.3798	R_1 = 0.1243, wR_2 = 0.2995
**Largest diff. peak/hole / e Å^{-3}**	3.30/-0.99	4.39/-0.96

Table S2: Crystallographic Data of complexes **C3** and **C4.**

	**C3**	**C4**
**Empirical formula**	$C_{50}H_{54}Cl_8Co_2F_{12}N_{10}O_6P_2$	$C_{94}H_{83}B_2Cl_8Co_2N_9O_4$
**Formula weight**	1582.43	1825.77
**Temperature/K**	120.00	120.00
**Crystal system**	triclinic	monoclinic
**Space group**	P-1	$P2_1/c$
**a/Å**	9.8551(3)	19.6985(3)
**b/Å**	11.7547(3)	9.9079(3)
**c/Å**	13.5213(4)	21.3537(4)
**α/°**	96.438(2)	90
**β/°**	94.033(2)	98.1760(10)
**γ/°**	98.114(2)	90
**Volume/Å^3**	1534.98(8)	4125.26(16)
**Z**	1	2
**ρ_{calc}g/cm^3**	1.712	1.470
**μ/mm^{-1}**	1.035	0.722
**F(000)**	800.0	1884.0
**Crystal size/mm^3**	0.47 × 0.217 × 0.06	0.24 × 0.177 × 0.06
**Radiation**	MoKα (λ = 0.71073)	MoKα (λ = 0.71073)
**2Θ range for data collection/°**	4.364 to 65.222	4.54 to 65.326
**Index ranges**	-14 ≤ h ≤ 13, -17 ≤ k ≤ 17, -19 ≤ l ≤ 20	-28 ≤ h ≤ 29, -13 ≤ k ≤ 12, -27 ≤ l ≤ 32
**Reflections collected**	29709	39358
**Independent reflections**	9623 [R_{int} = 0.0504, R_{sigma} = 0.0350]	12393 [R_{int} = 0.0310, R_{sigma} = 0.0285]
**Data/restraints/parameters**	9623/118/446	12393/195/581
**Goodness-of-fit on F^2**	1.086	1.043
**Final R indexes [I>=2σ (I)]**	R_1 = 0.0655, wR_2 = 0.1812	R_1 = 0.0498, wR_2 = 0.1289
**Final R indexes [all data]**	R_1 = 0.0686, wR_2 = 0.1863	R_1 = 0.0637, wR_2 = 0.1400
**Largest diff. peak/hole / e Å^{-3}**	2.92/-1.94	0.93/-0.57

Table S3: Crystallographic Data of complexes **C5**and **C6.**

	**C5**	**C6**
**Empirical formula**	$C_{48}H_{56}Br_8Co_2N_8O_{14}S$	$C_{48}H_{46}Br_8Cl_2Co_2N_{10}O_{12}$
**Formula weight**	1758.20	1782.99
**Temperature/K**	120	120.0
**Crystal system**	triclinic	triclinic
**Space group**	P-1	P-1
**a/Å**	10.2674(8)	10.1586(6)
**b/Å**	10.4742(9)	11.8117(7)
**c/Å**	14.4507(13)	12.5379(8)
**α/°**	105.210(7)	94.804(5)
**β/°**	89.460(7)	96.809(5)
**γ/°**	100.170(7)	97.067(5)
**Volume/Å^3**	1475.0(2)	1475.07(16)
**Z**	1	1
**ρ_{calc}g/cm^3**	1.979	2.007
**μ/mm^{-1}**	6.085	6.138
**F(000)**	862.0	868.0
**Crystal size/mm^3**	0.08 × 0.041 × 0.014	0.1 × 0.043 × 0.01
**Radiation**	MoKα (λ = 0.71073)	MoKα (λ = 0.71073)
**2Θ range for data collection/°**	4.034 to 67.734	4.074 to 69.586
**Index ranges**	-15 ≤ h ≤ 16, -15 ≤ k ≤ 16, -22 ≤ l ≤ 19	-15 ≤ h ≤ 14, -18 ≤ k ≤ 18, -19 ≤ l ≤ 18
**Reflections collected**	22389	23952
**Independent reflections**	9903 [R_{int} = 0.0594, R_{sigma} = 0.0937]	10972 [R_{int} = 0.0518, R_{sigma} = 0.0910]
**Data/restraints/parameters**	9903/99/399	10972/24/371
**Goodness-of-fit on F^2**	1.026	1.026
**Final R indexes [I>=2σ (I)]**	R_1 = 0.0858, wR_2 = 0.1985	R_1 = 0.0768, wR_2 = 0.1814
**Final R indexes [all data]**	R_1 = 0.1684, wR_2 = 0.2405	R_1 = 0.1578, wR_2 = 0.2210
**Largest diff. peak/hole / e Å^{-3}**	1.64/-1.15	2.37/-1.21

Table S4: Crystallographic Data of complexes **C7**and **C8.**

	**C7**	**C8**
**Empirical formula**	$C_{50}H_{54}Br_8Co_2F_{12}N_{10}O_6P_2$	$C_{100}H_{92}B_2Br_8Co_2N_{12}O_4$
**Formula weight**	1938.11	2304.61
**Temperature/K**	120.00	120.00
**Crystal system**	triclinic	monoclinic
**Space group**	P-1	$P2_1/n$
**a/Å**	10.1114(3)	12.9431(4)
**b/Å**	11.8311(4)	13.5336(4)
**c/Å**	13.5809(4)	27.5860(10)
**α/°**	97.436(2)	90
**β/°**	93.884(2)	96.206(3)
**γ/°**	97.718(2)	90
**Volume/Å^3**	1590.41(9)	4803.8(3)
**Z**	1	2
**ρ_{calc}g/cm^3**	2.024	1.593
**μ/mm^{-1}**	5.688	3.731
**F(000)**	944.0	2304.0
**Crystal size/mm^3**	0.1 × 0.07 × 0.02	0.27 × 0.162 × 0.045
**Radiation**	MoKα (λ = 0.71073)	MoKα (λ = 0.71073)
**2Θ range for data collection/°**	4.08 to 65.416	4.228 to 65.472
**Index ranges**	-14 ≤ h ≤ 13, -15 ≤ k ≤ 17, -20 ≤ l ≤ 20	-17 ≤ h ≤ 18, -18 ≤ k ≤ 18, -37 ≤ l ≤ 36
**Reflections collected**	32008	49572
**Independent reflections**	9996 [R_{int} = 0.0379, R_{sigma} = 0.0308]	14818 [R_{int} = 0.0231, R_{sigma} = 0.0242]
**Data/restraints/parameters**	9996/93/398	14818/0/579
**Goodness-of-fit on F^2**	1.063	1.057
**Final R indexes [I>=2σ (I)]**	R_1 = 0.0417, wR_2 = 0.1117	R_1 = 0.0359, wR_2 = 0.0846
**Final R indexes [all data]**	R_1 = 0.0498, wR_2 = 0.1173	R_1 = 0.0457, wR_2 = 0.0895
**Largest diff. peak/hole / e Å^{-3}**	1.05/-1.37	1.18/-1.19

11. Bond Length Tables

Table S5: Metal-Donor bond lengths of **C1**.

**C1**	**Bond length / Å**		**Bond length / Å**
**Co1 O1**	1.881(6)	**Co2 O3**	1.889(6)
**Co1 O2**	1.898(6)	**Co2 O4**	1.895(6)
**Co1 N1**	1.933(8)	**Co2 N5**	2.045(8)
**Co1 N2**	1.924(7)	**Co2 N6**	1.919(8)
**Co1 N3**	1.922(8)	**Co2 N7**	1.926(8)
**Co1 N4**	2.043(7)	**Co2 N8**	1.928(7)

Table S6: Metal-Donor bond lengths of **C2**.

**C2**	**Bond length / Å**		**Bond length / Å**
**Co1 O1**	1.898(4)	**Co2 O3**	1.895(4)
**Co1 O2**	1.871(4)	**Co2 O4**	1.873(4)
**Co1 N1**	1.936(4)	**Co2 N5**	1.938(4)
**Co1 N2**	1.910(4)	**Co2 N6**	1.921(4)
**Co1 N3**	1.916(4)	**Co2 N7**	1.914(4)
**Co1 N4**	2.048(4)	**Co2 N8**	2.045(4)

Table S7: Metal-Donor bond lengths of **C3** and **C4**.

**C3**	**Bond length / Å**	**C4**	**Bond length / Å**
**Co1 O1**	1.8816(14)	**Co1 O1**	1.8708(13)
**Co1 O2**	1.8966(15)	**Co1 O2**	1.8909(15)
**Co1 N1**	1.9399(16)	**Co1 N1**	1.9454(17)
**Co1 N2**	1.9081(18)	**Co1 N2**	1.9200(18)
**Co1 N3**	1.9171(17)	**Co1 N3**	1.9173(19)
**Co1 N4**	2.0479(17)	**Co1 N4**	2.0555(17)

Table S8: Metal-Donor bond lengths of **C5** and **C6**.

C5	Bond length / Å	C6	Bond length / Å
**Co1 O1**	1.912(5)	**Co1 O1**	1.898(5)
**Co1 O2**	1.893(5)	**Co1 O2**	1.882(4)
**Co1 N1**	1.957(6)	**Co1 N1**	1.942(5)
**Co1 N2**	1.925(7)	**Co1 N2**	1.902(6)
**Co1 N3**	1.937(6)	**Co1 N3**	1.923(6)
**Co1 N4**	2.056(6)	**Co1 N4**	2.107(6)

Table S9: Metal-Donor bond lengths of **C7** and **C8**.

C7	Bond length / Å	C8	Bond length / Å
**Co1 O1**	1.8722(18)	**Co1 O1**	1.8773(13)
**Co1 O2**	1.8918(18)	**Co1 O2**	1.8745(13)
**Co1 N1**	1.937(2)	**Co1 N1**	1.9460(15)
**Co1 N2**	1.915(2)	**Co1 N2**	1.9170(16)
**Co1 N3**	1.914(2)	**Co1 N3**	1.9251(15)
**Co1 N4**	2.047(2)	**Co1 N4**	2.0376(15)

Table S10: C-C and C-O Bond lengths of the catechols of **C1**.

C1	Bond length / Å		Bond length / Å
**C33 C34**	1.383(13)	**C39 C40**	1.389(12)
**C34 C35**	1.399(13)	**C40 C41**	1.407(14)
**C35 C36**	1.387(14)	**C41 C42**	1.375(14)
**C36 C37**	1.418(13)	**C42 C43**	1.410(14)
**C37 C38**	1.398(12)	**C43 C44**	1.418(13)
**C38 C33**	1.438(13)	**C44 C39**	1.410(13)
**O1 C33**	1.327(11)	**O3 C39**	1.337(11)
**O2 C38**	1.328(10)	**O4 C44**	1.319(11)

Table S11: C-C and C-O Bond lengths of the catechols of **C2**.

**C2**	**Bond length / Å**		**Bond length / Å**
**C17 C18**	1.418(7)	**C39 C44**	1.422(8)
**C17 C22**	1.398(8)	**C40 C41**	1.395(8)
**C18 C19**	1.405(7)	**C41 C42**	1.380(9)
**C19 C20**	1.381(9)	**C42 C43**	1.411(7)
**C20 C21**	1.403(7)	**C43 C44**	1.393(7)
**C21 C22**	1.393(7)	**C40 C39**	1.397(7)
**O1 C17**	1.336(6)	**O3 C39**	1.332(6)
**O2 C22**	1.342(6)	**O4 C44**	1.332(6)

Table S12: C-C and C-O Bond lengths of the catechols of **C3** and **C4**.

**C3**	**Bond length / Å**	**C4**	**Bond length / Å**
**C17 C18**	1.392(3)	**C17 C18**	1.386(3)
**C18 C19**	1.397(3)	**C17 C22**	1.415(3)
**C19 C20**	1.393(3)	**C18 C19**	1.395(3)
**C20 C21**	1.400(3)	**C19 C20**	1.396(3)
**C21 C22**	1.396(3)	**C20 C21**	1.402(3)
**C22 C17**	1.419(3)	**C21 C22**	1.390(3)
**O1 C17**	1.331(2)	**O1 C17**	1.335(2)
**O2 C22**	1.332(2)	**O2 C22**	1.336(2)

Table S13: C-C and C-O Bond lengths of the catechols of **C5** and **C6**.

**C5**	**Bond length / Å**	**C6**	**Bond length / Å**
**C17 C18**	1.395(10)	**C17 C18**	1.409(9)
**C17 C22**	1.411(10)	**C17 C22**	1.404(9)
**C18 C19**	1.401(10)	**C18 C19**	1.420(9)
**C19 C20**	1.384(11)	**C19 C20**	1.381(10)
**C20 C21**	1.397(10)	**C20 C21**	1.419(9)
**C21 C22**	1.399(10)	**C21 C22**	1.395(9)
**O1 C17**	1.336(8)	**O1 C17**	1.328(7)

**O2 C22**	1.327(9)	**O2 C22**	1.341(7)

Table S14: C-C and C-O Bond lengths of the catechols of **C7** and **C8**.

**C7**	**Bond length / Å**	**C8**	**Bond length / Å**
**C17 C18**	1.385(4)	**C17 C18**	1.389(3)
**C18 C19**	1.399(3)	**C17 C22**	1.412(2)
**C19 C20**	1.387(4)	**C18 C19**	1.398(3)
**C20 C21**	1.406(4)	**C19 C20**	1.390(3)
**C21 C22**	1.387(4)	**C20 C21**	1.395(3)
**C22 C17**	1.417(3)	**C21 C22**	1.387(3)
**O1 C17**	1.338(3)	**O1 C17**	1.335(2)
**O2 C22**	1.332(3)	**O2 C22**	1.338(2)

12. Metal-Donor-Bond Angles

Table S15: Metal-Donor bond angles of **C1**.

**C1**	**Bond Angle /°**	**C1**	**Bond Angle /°**
**O1 Co1 O2**	88.3(3)	**O3 Co2 O4**	87.6(3)
**O1 Co1 N4**	94.2(3)	**O3 Co2 N5**	92.3(3)
**O1 Co1 N2**	94.2(4)	**O3 Co2 N6**	95.0(4)
**O1 Co1 N3**	94.2(4)	**O3 Co2 N7**	94.9(4)
**O1 Co1 N1**	178.1(4)	**O3 Co2 N8**	178.3(4)
**O2 Co1 N4**	92.4(3)	**O4 Co2 N5**	92.3(3)
**O2 Co1 N2**	174.2(4)	**O4 Co2 N6**	85.4(4)
**O2 Co1 N3**	86.9(4)	**O4 Co2 N7**	173.4(4)
**O2 Co1 N1**	90.4(3)	**O4 Co2 N8**	90.7(4)
**N2 Co1 N4**	92.6(4)	**N6 Co2 N5**	172.3(4)
**N2 Co1 N1**	86.9(4)	**N7 Co2 N5**	93.7(4)
**N3 Co1 N4**	171.6(4)	**N7 Co2 N6**	88.2(4)
**N3 Co1 N2**	87.7(4)	**N7 Co2 N8**	86.8(4)
**N3 Co1 N1**	84.3(4)	**N8 Co2 N5**	87.9(4)

Table S16: Metal-Donor bond angles of **C2**.

**C2**	**Bond Angle /°**	**C2**	**Bond Angle /°**
**O1 Co 1N1**	91.53(18)	**O3 Co2 N5**	92.32(17)
**O1 Co1 N2**	175.81(18)	**O3 Co2 N6**	175.87(17)
**O1 Co1 N3**	85.75(18)	**O3 Co2 N7**	85.56(17)
**O1 Co1 N4**	92.50(17)	**O3 Co2 N8**	92.32(16)
**O2 Co1 O1**	87.92(16)	**O4 Co2 O3**	87.86(16)
**O2 Co1 N1**	179.03(18)	**O4 Co2 N5**	179.38(18)
**O2 Co1 N2**	93.96(18)	**O4 Co2 N6**	93.26(17)
**O2 Co1 N3**	95.17(18)	**O4 Co2 N7**	95.64(17)
**O2 Co1 N4**	91.74(17)	**O4 Co2 N8**	91.89(16)
**N1 Co1 N4**	89.08(18)	**N5 Co2 N8**	88.70(17)
**N2 Co1 N1**	86.5(2)	**N6 Co2 N5**	86.52(19)
**N2 Co1 N3**	90.34(19)	**N6 Co2 N8**	91.63(17)
**N2 Co1 N4**	91.19(18)	**N7 Co2 N5**	83.78(19)
**N3 Co1 N1**	83.99(19)	**N7 Co2 N6**	90.36(18)

Table S17: Metal-Donor bond angles of **C3** and **C4**.

C3	Bond Angle /°	C4	Bond Angle /°
**O1 Co1 O2**	87.88(6)	**O1 Co1 O2**	88.59(6)
**O1 Co1 N4**	92.41(6)	**O1 Co1 N1**	178.22(7)
**O2 Co1 N1**	91.94(7)	**O1 Co1 N2**	92.90(7)
**O2 Co1 N2**	175.58(6)	**O1 Co1 N3**	93.87(7)
**O2 Co1 N3**	85.46(7)	**O1 Co1 N4**	93.34(6)
**O2 Co1 N4**	92.76(7)	**O2 Co1 N1**	92.27(7)
**N1 Co1 N4**	88.16(7)	**O2 Co1 N2**	176.70(7)
**N2 Co1 N1**	86.78(7)	**O2 Co1 N3**	86.99(8)
**N2 Co1 N3**	90.20(7)	**O2 Co1 N4**	90.08(6)
**N2 Co1 N4**	91.43(7)	**N1 Co1 N4**	88.22(7)
**N3 Co1 N1**	84.00(7)	**N2 Co1 N1**	86.17(8)
**N3 Co1 N4**	171.89(7)	**N2 Co1 N4**	92.77(7)
**O1 Co1 O2**	87.88(6)	**N3 Co1 N1**	84.62(8)
**O1 Co1 N4**	92.41(6)	**N3 Co1 N2**	89.97(8)

Table S18: Metal-Donor bond angles of **C5** and **C6**.

C5	Bond Angle /°	C6	Bond Angle /°
**O1 Co1 N1**	92.9(3)	**O1 Co1 N1**	92.4(2)
**O1 Co1 N2**	177.5(2)	**O1 Co1 N2**	176.2(2)
**O1 Co1 N3**	87.0(2)	**O1 Co1 N3**	87.0(2)
**O1 Co1 N4**	90.9(3)	**O1 Co1 N4**	93.5(2)
**O2 Co1 O1**	87.2(2)	**O2 Co1 O1**	87.23(19)
**O2 Co1 N1**	177.5(2)	**O2 Co1 N1**	175.9(2)
**O2 Co1 N2**	93.8(3)	**O2 Co1 N2**	93.7(2)
**O2 Co1 N3**	94.0(2)	**O2 Co1 N3**	93.0(2)
**O2 Co1 N4**	94.2(2)	**O2 Co1 N4**	96.7(2)
**N1 Co1 N4**	88.3(3)	**N1 Co1 N4**	87.5(2)
**N2 Co1 N1**	86.0(3)	**N2 Co1 N1**	86.4(2)
**N2 Co1 N3**	90.6(3)	**N2 Co1 N3**	89.3(2)
**N2 Co1 N4**	91.4(3)	**N2 Co1 N4**	90.0(2)
**N3 Co1 N1**	83.5(3)	**N3 Co1 N1**	82.9(2)

Table S19: Metal-Donor bond angles of **C7** and **C8**.

C7	Bond Angle /°	C8	Bond Angle /°
**O1 Co1 O2**	87.90(8)	**O1 Co1 N1**	92.94(6)
**O1 Co1 N1**	179.28(9)	**O1 Co1 N2**	175.97(6)
**O1 Co1 N2**	93.15(9)	**O1 Co1 N3**	86.11(6)
**O1 Co1 N3**	95.26(9)	**O1 Co1 N4**	91.38(6)
**O1 Co1 N4**	91.96(8)	**O2 Co1 O1**	88.37(6)
**O2 Co1 N1**	92.40(9)	**O2 Co1 N1**	177.99(6)
**O2 Co1 N2**	175.77(9)	**O2 Co1 N2**	91.76(6)
**O2 Co1 N3**	85.51(9)	**O2 Co1 N3**	95.92(6)
**O2 Co1 N4**	91.95(8)	**O2 Co1 N4**	93.91(6)
**N1 Co1 N4**	88.68(9)	**N1 Co1 N4**	87.58(6)
**N2 Co1 N1**	86.50(9)	**N2 Co1 N1**	86.83(7)
**N2 Co1 N4**	92.11(9)	**N2 Co1 N3**	89.87(7)
**N3 Co1 N1**	84.11(9)	**N2 Co1 N4**	92.63(6)
**N3 Co1 N2**	90.31(9)	**N3 Co1 N1**	82.9(2)
**N3 Co1 N4**	172.25(9)	**N3 Co1 N4**	169.78(6)

13. SHAPE Measurements

Table S20: Continuous shape measurements of **C1** on the Co1 center.

C1 Co1	OC-6	Ideal Structure	CShM =	0.25530
Co	M	10.9739	24.9100	24.2591
N	L1	10.2254	23.1767	23.8554
O	L2	10.5576	25.5064	22.4706
O	L3	9.2435	25.5163	24.8640
N	L5	12.7042	24.3037	23.6542
N	L6	11.7224	26.6433	24.6628
N	L4	11.3902	24.3137	26.0476

Table S21: Continuous shape measurements of **C1** on the Co2 center.

C1 Co2	OC-6	Ideal Structure	CShM =	0.28522
Co	M	7.9189	19.1752	21.9174
N	L1	9.6350	18.5775	21.2644
O	L2	8.4335	18.6896	23.7140
O	L3	8.6392	20.9459	22.1896
N	L5	7.1987	17.4045	21.6451
N	L6	6.2029	19.7728	22.5703
N	L4	7.4044	19.6607	20.1207

Table S22: Continuous shape measurements of **C2** on the Co1 center.

C2 Co1	OC-6	Ideal Structure	CShM =	0.25070
Co	M	5.2538	6.5832	23.6624
O	L1	6.9863	5.7391	23.6963
O	L2	5.9991	8.0740	22.6942
N	L4	4.5086	5.0924	24.6306
N	L6	3.5213	7.4273	23.6285
N	L3	4.8560	5.6999	21.9961
N	L5	5.6516	7.4665	25.3287

Table S23: Continuous shape measurements of **C2** on the Co2 center.

C2 Co2	OC-6	Ideal Structure	CShM =	0.27405
Co	M	6.4582	9.0403	42.0710
N	L1	5.9835	8.3043	40.3532
O	L2	4.7379	9.9049	42.1759
O	L3	5.7280	7.4819	42.9405
N	L5	7.1884	10.5987	41.2015
N	L4	8.1785	8.1758	41.9661
N	L6	6.9329	9.7763	43.7888

Table S24: Continuous shape measurements of **C3** on the Co1 center.

**C3 Co1**	**OC-6**	**Ideal Structure**	**CShM =**	**0.27444**
Co	M	-0.2251	6.8457	9.3970
O	L1	-0.9377	5.2924	10.2919
O	L2	-1.9675	7.6723	9.4441
N	L6	0.4876	8.3991	8.5021
N	L4	1.5174	6.0192	9.3499
N	L3	0.1963	7.6366	11.1054
N	L5	-0.6465	6.0548	7.6886

Table S25: Continuous shape measurements of **C4** on the Co1 center.

**C4 Co1**	**OC-6**	**Ideal Structure**	**CShM =**	**0.23856**
Co	M	1.7125	4.0381	11.4350
O	L1	0.8383	3.9349	13.1537
O	L2	1.1940	2.2158	11.0618
N	L6	2.5867	4.1414	9.7162
N	L4	2.2310	5.8605	11.8082
N	L3	3.3544	3.4077	12.2322
N	L5	0.0706	4.6686	10.6378

Table S26: Continuous shape measurements of **C5** on the Co1 center.

**C5 Co1**	**OC-6**	**Ideal Structure**	**CShM =**	**0.26684**
Co	M	5.5890	1.9207	4.0316
N	L1	5.0486	2.4116	5.8329
O	L2	3.8498	1.1064	3.7317
O	L3	4.9101	3.6159	3.3659
N	L5	6.2679	0.2255	4.6973
N	L4	7.3282	2.7350	4.3315
N	L6	6.1295	1.4298	2.2303

Table S27: Continuous shape measurements of **C6** on the Co1 center.

**C6 Co1**	**OC-6**	**Ideal Structure**	**CShM =**	**0.41186**
Co	M	4.6076	7.3116	8.3205
O	L1	2.8618	8.1529	8.2609
O	L2	3.8226	5.7407	9.1419
N	L4	5.3926	8.8826	7.4991
N	L6	6.3534	6.4704	8.3800
N	L3	4.9157	8.0754	10.0757
N	L5	4.2994	6.5479	6.5653

Table S28: Continuous shape measurements of **C7** on the Co1 center.

**C7 Co1**	**OC-6**	**Ideal Structure**	**CShM =**	**0.27241**
Co	M	2.6404	3.1036	4.0074
O	L1	3.3790	4.6532	3.1320
O	L2	4.3648	2.2460	3.9443
N	L6	1.9017	1.5540	4.8828
N	L4	0.9159	3.9612	4.0705
N	L3	2.2000	2.3444	2.2919
N	L5	3.0807	3.8628	5.7229

Table S29: Continuous shape measurements of **C8** on the Co1 center.

**C8 Co1**	**OC-6**	**Ideal Structure**	**CShM =**	**0.33435**
Co	M	7.4372	-2.1044	14.9051
O	L1	6.2779	-2.3997	16.4146
O	L2	8.0340	-0.4486	15.6873
N	L4	6.8404	-3.7602	14.1229
N	L6	8.5965	-1.8091	13.3956
N	L3	8.8548	-3.0429	15.8102
N	L5	6.0196	-1.1658	14.0000

14. Metric Oxidation State (MOS)

Table S30: The MOS of the catecholate ligands of **C1-C8**.

Compound	C-O avg.	C1-C2	C2-C3 avg.	C3-C4 avg.	C4-C5	Calc. MOS
**C1 Co1**	1.326	1.438	1.391	1.409	1.387	-1.690
**C1 Co2**	1.3285	1.409	1.4035	1.408	1.375	-1.839
**C2 Co1**	1.339	1.397	1.4055	1.404	1.382	-1.907
**C2 Co2**	1.332	1.422	1.395	1.4035	1.380	-1.805
**C3**	1.3315	1.419	1.394	1.3985	1.393	-1.749
**C4**	1.3355	1.415	1.388	1.39985	1.396	-1.802
**C5**	1.331	1.411	1.397	1.399	1.384	-1.809
**C6**	1.3345	1.404	1.402	1.419	1.382	-1.895
**C7**	1.335	1.417	1.386	1.403	1.387	-1.844
**C8**	1.337	1.412	1.388	1.3965	1.391	-1.838

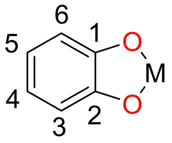

15. Crystal Structures

15.1 Complexes

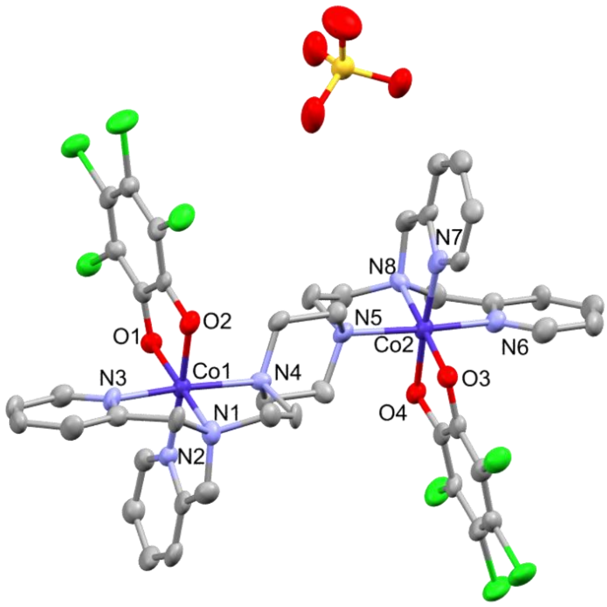

Figure S38: Complex structure of **C1** at 120 K. Lattice solvent molecules and hydrogen atoms are omitted for better visibility. The probability level for the displacement of the ellipsoid is 50%. Colour code: carbon (grey), cobalt (dark blue), nitrogen (light blue), oxygen (red), chloride (green), sulphur (yellow).

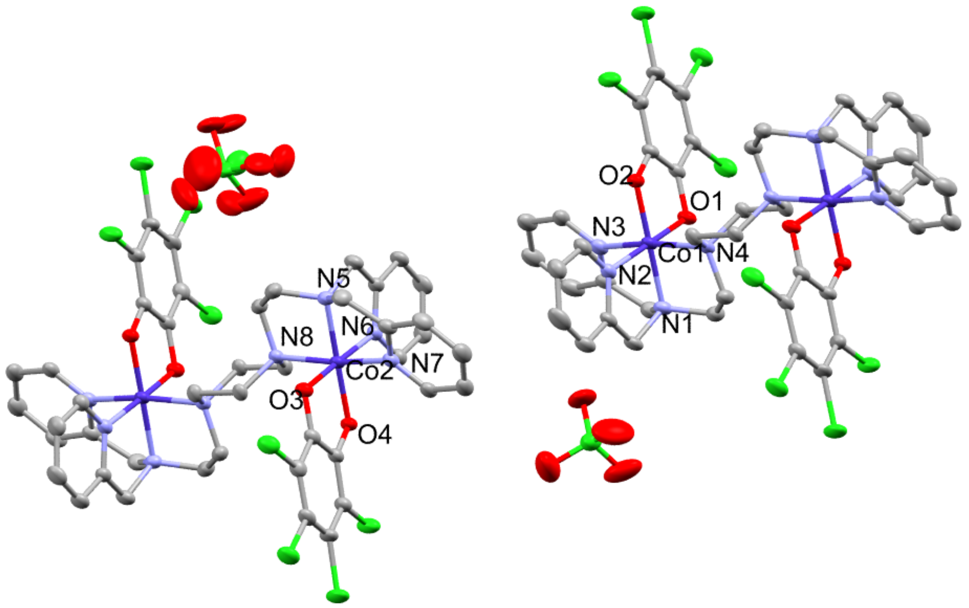

Figure S39: Complex structure of **C2** at 120 K. Lattice solvent molecules and hydrogen atoms are omitted for better visibility. The probability level for the displacement of the ellipsoid is 50%. Colour code: carbon (grey), cobalt (dark blue), nitrogen (light blue), oxygen (red), chloride (green).

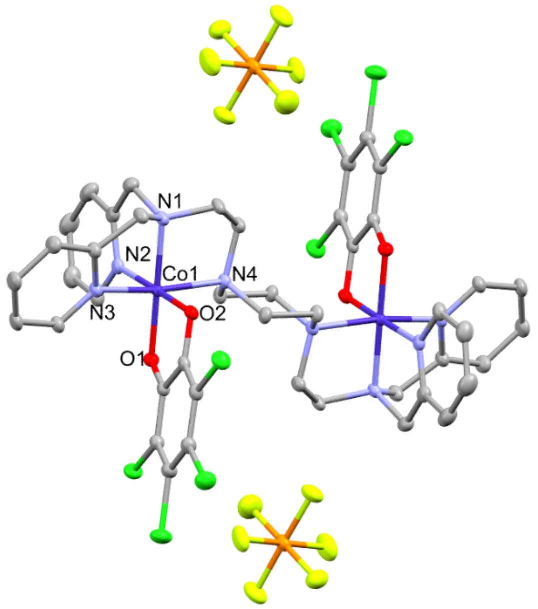

Figure S40: Complex structure of **C3** at 120 K. Lattice solvent molecules and hydrogen atoms are omitted for better visibility. The probability level for the displacement of the ellipsoid is 50%. Colour code: carbon (grey), cobalt (dark blue), nitrogen (light blue), oxygen (red), chloride (green), phosphorous (orange), fluorine (light green).

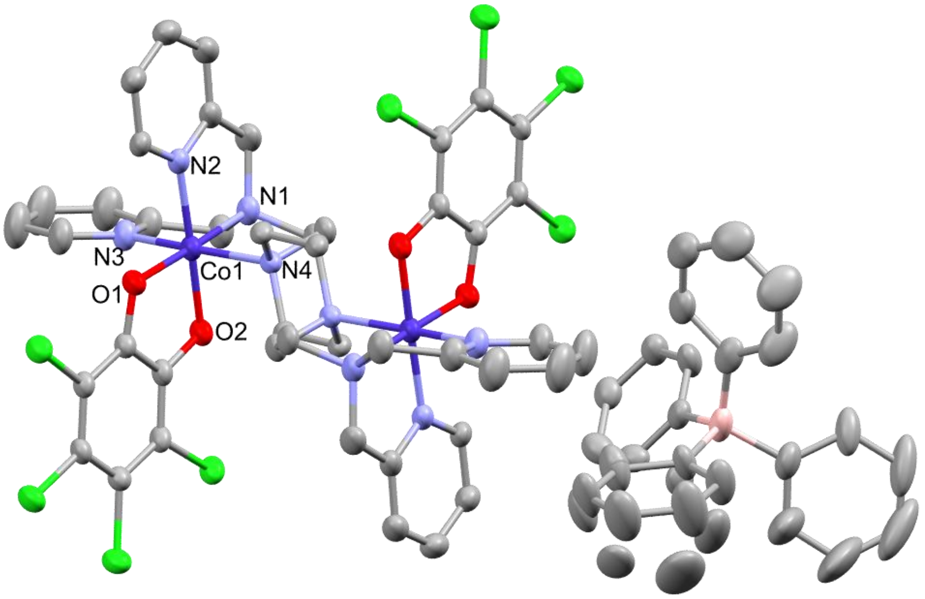

Figure S41: Complex structure of **C4** at 120 K. Lattice solvent molecules and hydrogen atoms are omitted for better visibility. The probability level for the displacement of the ellipsoid is 50%. Colour code: carbon (grey), cobalt (dark blue), nitrogen (light blue), oxygen (red), chloride (green), boron (rosa).

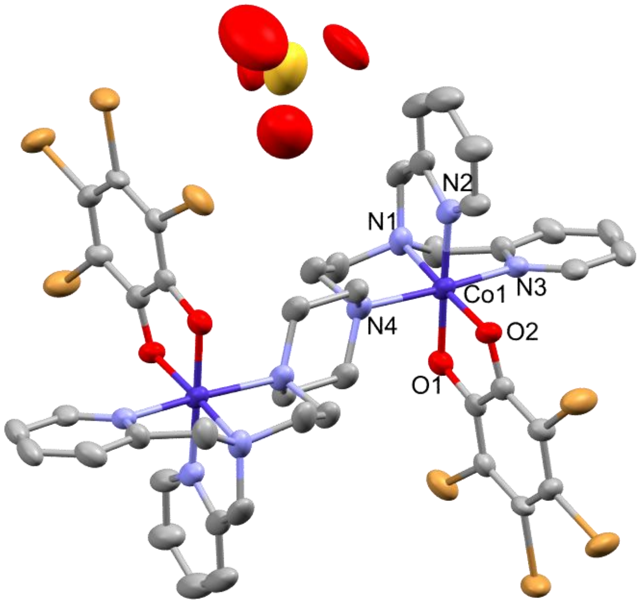

Figure S42: Complex structure of **C5** at 120 K. Lattice solvent molecules and hydrogen atoms are omitted for better visibility. The probability level for the displacement of the ellipsoid is 50%. Colour code: carbon (grey), cobalt (dark blue), nitrogen (light blue), oxygen (red), bromine (brown), sulphur (yellow).

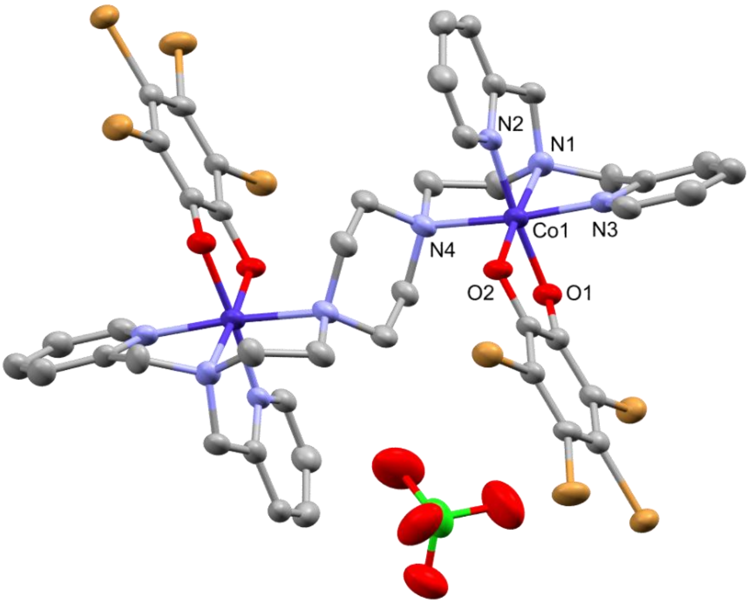

Figure S43: Complex structure of **C6** at 120 K. Lattice solvent molecules and hydrogen atoms are omitted for better visibility. The probability level for the displacement of the ellipsoid is 50%. Colour code: carbon (grey), cobalt (dark blue), nitrogen (light blue), oxygen (red), bromine (brown), chlorine (green).

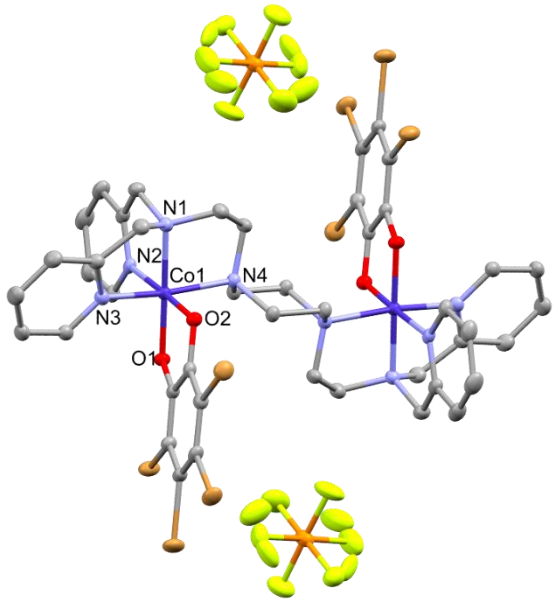

Figure S44: Complex structure of **C7** at 120 K. Lattice solvent molecules and hydrogen atoms are omitted for better visibility. The probability level for the displacement of the ellipsoid is 50%. Colour code: carbon (grey), cobalt (dark blue), nitrogen (light blue), oxygen (red), bromine (brown), phosphorous (orange), fluorine (light green).

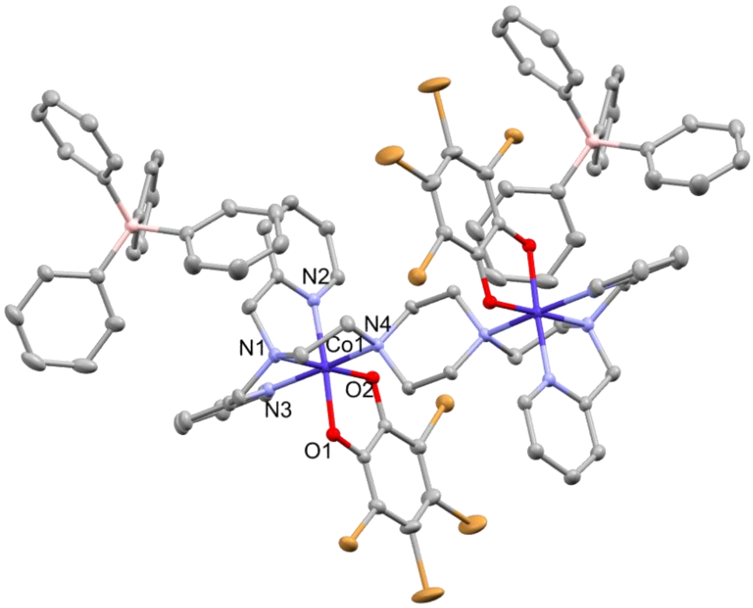

Figure S45: Complex structure of **C8** at 120 K. Lattice solvent molecules and hydrogen atoms are omitted for better visibility. The probability level for the displacement of the ellipsoid is 50%. Colour code: carbon (grey), cobalt (dark blue), nitrogen (light blue), oxygen (red), bromine (brown), boron (pink).

15.2 Asymmetric unit cells

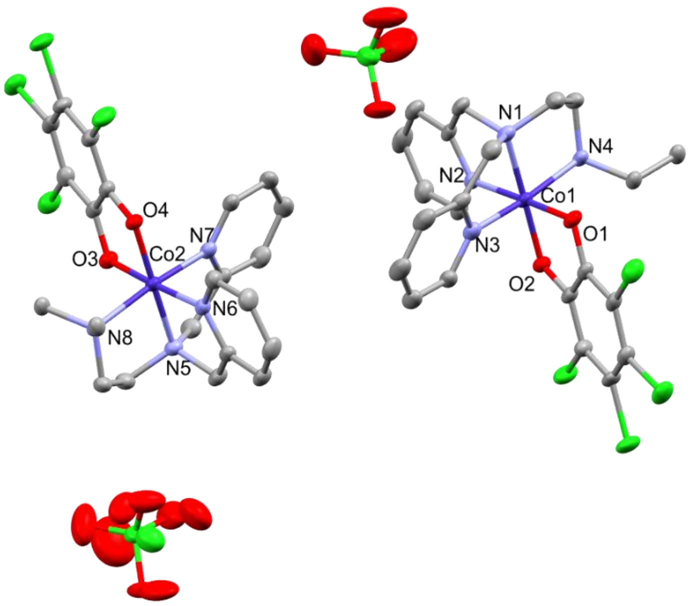

Figure S46: Asymmetric unit cell of **C2** at 120 K. Lattice solvent molecules and hydrogen atoms are omitted for better visibility. The probability level for the displacement of the ellipsoid is 50%. Colour code: carbon (grey), cobalt (dark blue), nitrogen (light blue), oxygen (red), chloride (green).

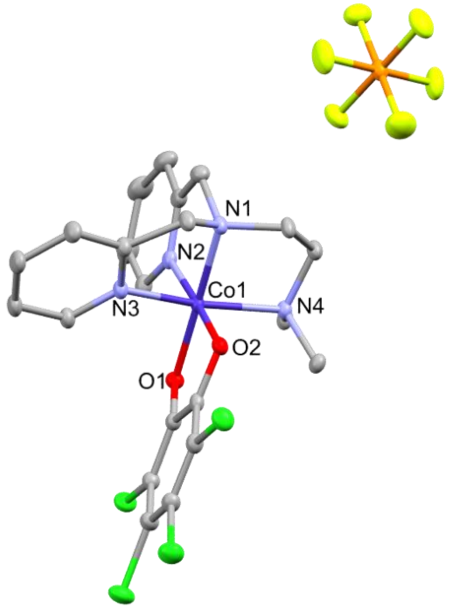

Figure S47: Asymmetric unit cell of **C3** at 120 K. Lattice solvent molecules and hydrogen atoms are omitted for better visibility. The probability level for the displacement of the ellipsoid is 50%. Colour code: carbon (grey), cobalt (dark blue), nitrogen (light blue), oxygen (red), chloride (green), phosphorous (orange), fluorine (light green).

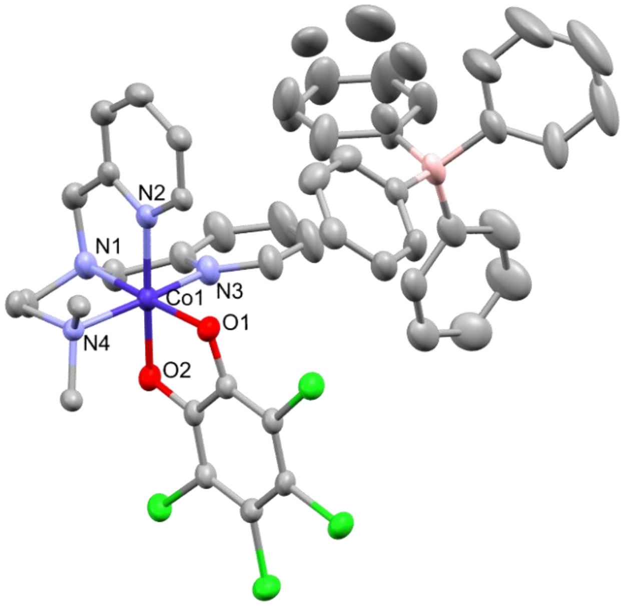

Figure S48: Asymmetric unit cell of **C4** at 120 K. Lattice solvent molecules and hydrogen atoms are omitted for better visibility. The probability level for the displacement of the ellipsoid is 50%. Colour code: carbon (grey), cobalt (dark blue), nitrogen (light blue), oxygen (red), chloride (green), boron (pink).

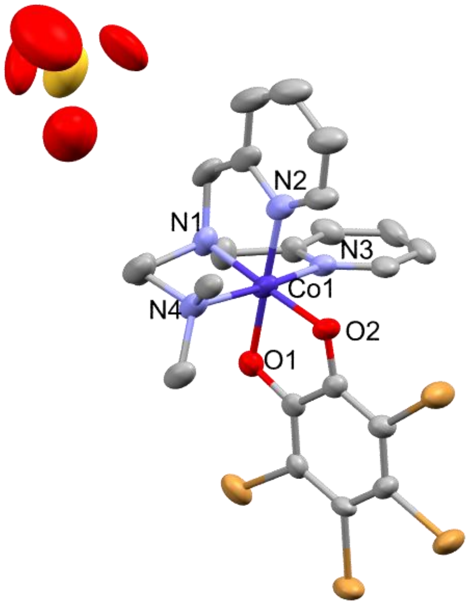

Figure S49: Asymmetric unit cell of **C5** at 120 K. Lattice solvent molecules and hydrogen atoms are omitted for better visibility. The probability level for the displacement of the ellipsoid is 50%. Colour code: carbon (grey), cobalt (dark blue), nitrogen (light blue), oxygen (red), bromine (brown), sulphur (yellow).

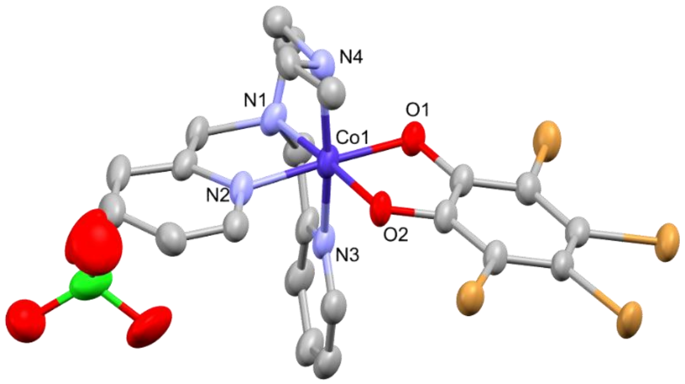

Figure S50: Asymmetric unit cell of **C6** at 120 K. Lattice solvent molecules and hydrogen atoms are omitted for better visibility. The probability level for the displacement of the ellipsoid is 50%. Colour code: carbon (grey), cobalt (dark blue), nitrogen (light blue), oxygen (red), bromine (brown), chlorine (green).

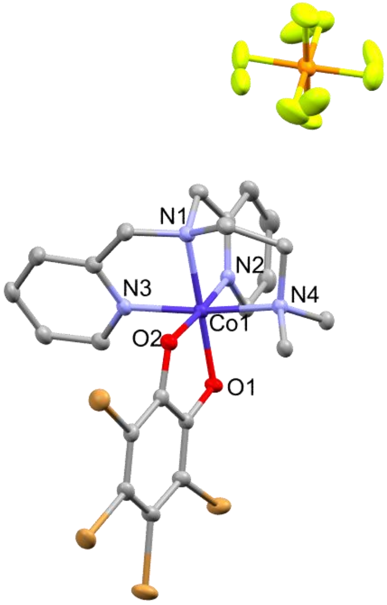

Figure S51: Asymmetric unit cell of **C7** at 120 K. Lattice solvent molecules and hydrogen atoms are omitted for better visibility. The probability level for the displacement of the ellipsoid is 50%. Colour code: carbon (grey), cobalt (dark blue), nitrogen (light blue), oxygen (red), bromine (brown), phosphorous (orange), fluorine (light green).

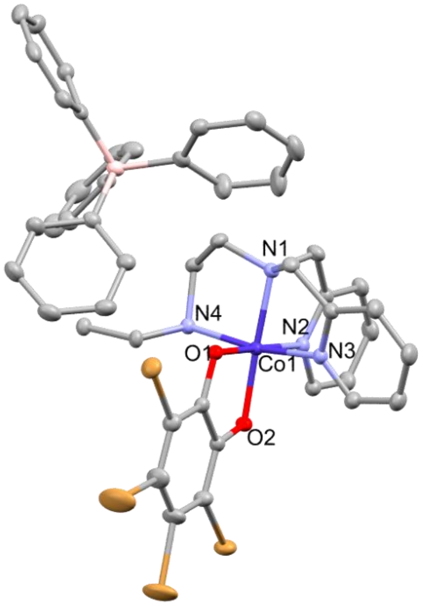

Figure S52: Asymmetric unit cell of **C8** at 120 K. Lattice solvent molecules and hydrogen atoms are omitted for better visibility. The probability level for the displacement of the ellipsoid is 50%. Colour code: carbon (grey), cobalt (dark blue), nitrogen (light blue), oxygen (red), bromine (brown), boron (rosa).

15.3 π-π interactions

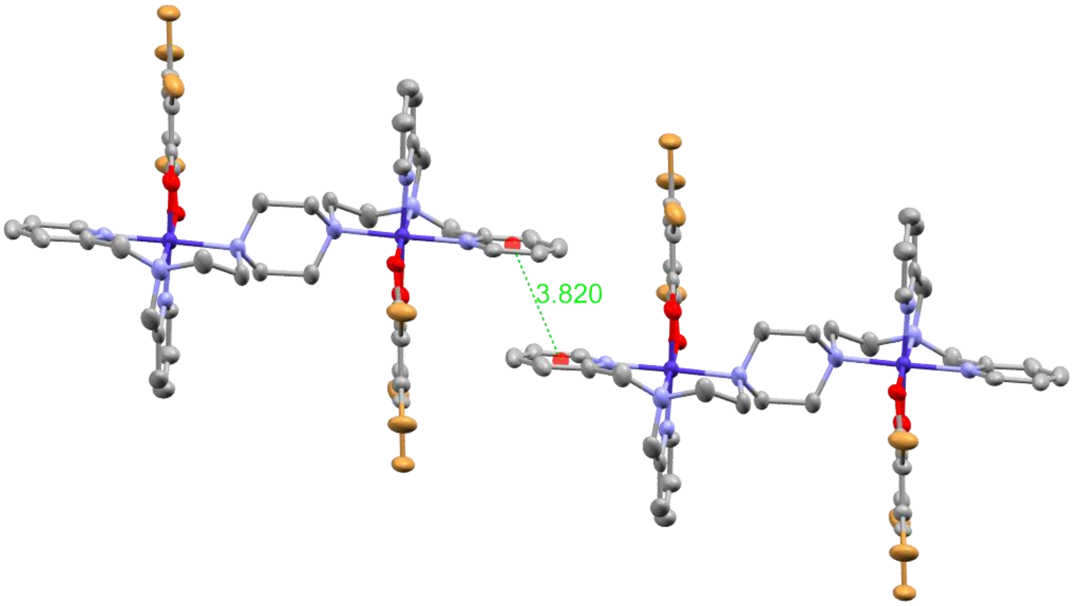

Figure S53: π-π interactions of **C5** with a centroid-to-centroid distance of 3.820 Å, a parallel shift of 1.743 Å and an angle of 0° between both pyridine planes at 120 K. Lattice solvent molecules and hydrogen atoms are omitted for better visibility. The probability level for the displacement of the ellipsoid is 50%. Colour code: carbon (grey), cobalt (dark blue), nitrogen (light blue), oxygen (red), bromine (brown), sulphur (yellow).

15.4 Hydrogen Bonding Interactions

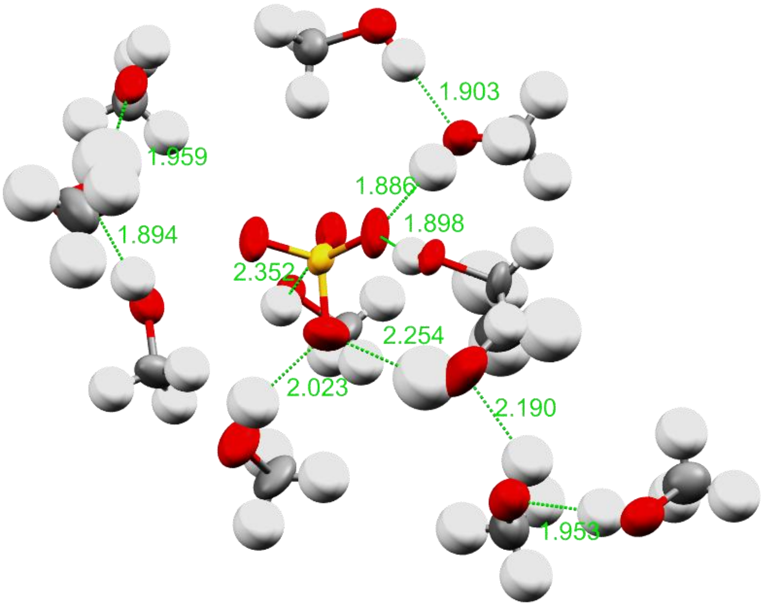

Figure S54: Hydrogen bonding interaction between methanol molecules and the sulphate anion of **C1** at 120 K. Complex fragment was omitted for better visibility. The probability level for the displacement of the ellipsoid is 50%. Colour code: carbon (grey), cobalt (dark blue), nitrogen (light blue), oxygen (red), chloride (green), sulphur (yellow).

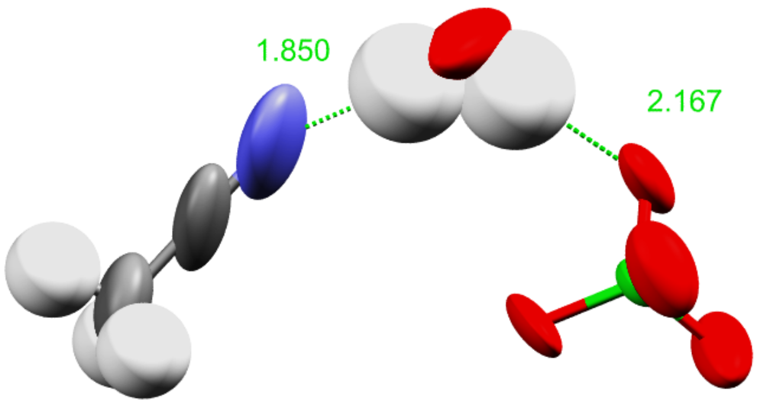

Figure S55: Hydrogen bonding interactions between solvent molecules and anions of **C2** at 120 K. Complex fragment was omitted for better visibility. The probability level for the displacement of the ellipsoid is 50%. Colour code: carbon (grey), cobalt (dark blue), nitrogen (light blue), oxygen (red), chloride (green).

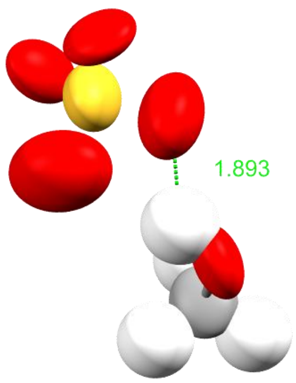

Figure S56: Hydrogen bonding interactions between methanol and sulphate of **C5** at 120 K. Lattice solvent molecules and hydrogen atoms are omitted for better visibility. The probability level for the displacement of the ellipsoid is 50%. Colour code: carbon (grey), cobalt (dark blue), nitrogen (light blue), oxygen (red), bromine (brown), sulphur (yellow).

15.5 H-F-Bonding Interactions

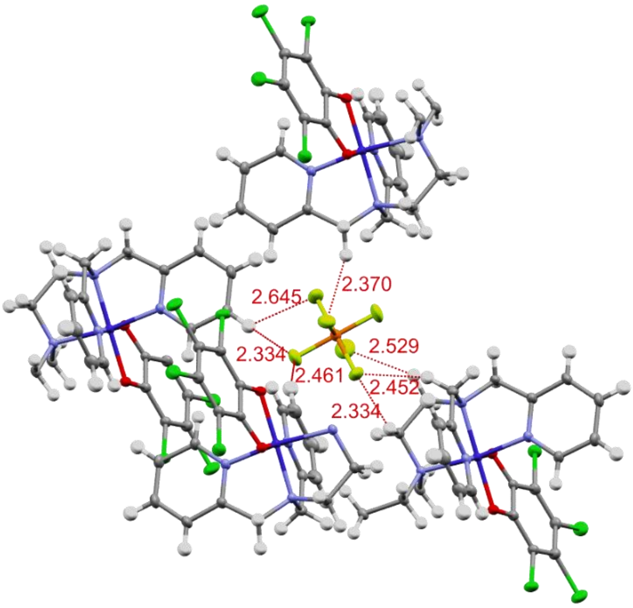

Figure S57: H-F-Bonding interactions of **C3** at 120 K. Lattice solvent molecules and hydrogen atoms are omitted for better visibility. The probability level for the displacement of the ellipsoid is 50%. Colour code: carbon (grey), cobalt (dark blue), nitrogen (light blue), oxygen (red), chloride (green), phosphorous (orange), fluorine (light green).

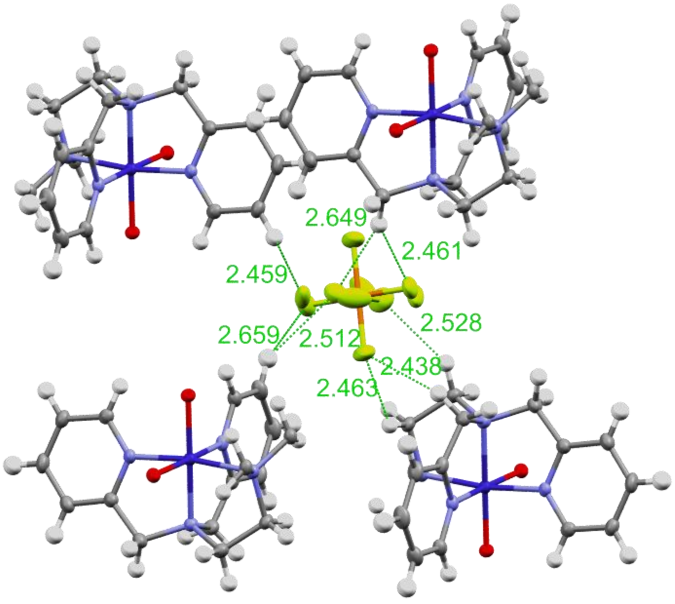

Figure S58: H-F-Bonding interactions of **C7** at 120 K. Lattice solvent are omitted for better visibility. The probability level for the displacement of the ellipsoid is 50%. Colour code: carbon (grey), cobalt (dark blue), nitrogen (light blue), oxygen (red), bromine (brown), phosphorous (orange), fluorine (light green).

15.6 Crystallographic planes

In terms of crystal packing, complexes **C3** and **C5-C7** shows a layered structure comprising a complex-anion-complex motif. In **C1** the layers can also be observed, but in the *b*-*c* plane the arrangement in *b* direction as well as in the *c* direction of the complexes are inverted. (**Figure S82**) In the *a*-*b*-plane complex **C1** shows a herringbone pattern but as the packing changes in *c* direction, the 2nd *a*-*b*-plane directly behind shows an inverted herringbone pattern, so that two complexes directly behind each other form a star shaped motif (**Figure S75**).

Complex **C4** also exhibits a herringbone pattern in the *b*-*c*-plane, but here all *b*-*c*-planes are stacked perfectly on top of each other without any inversion like in **C1**. (**Figure S82**) However, due to this herringbone pattern the *a*- *b*-planes are not stacked perfectly, 2 complexes in the *a*-*b*-planes directly behind each other are tilted by 65 degrees angle. (**Figure S77**). Complex **C8** also shows a herringbone pattern in the *a*-*b* plane. But this herringbone pattern is created due to the 2nd *a*-*b* plane behind being inverted (**Figure S81**). The crystallographic parameters, as well as the crystal structures with each plane, asymmetric unit and complete structure are shown in the ESI. (**Figures S53-S97, Tables S2-20**).

C8 also shows a herringbone pattern in the *a*-*b* plane. But this herringbone pattern is created due to the 2nd *a*-*b* plane behind being inverted as well as slightly shifted (**Figure S82**). The crystallographic parameters, as well as the crystal structures with every plane, asymmetric unit and complete structure are shown in the ESI. (**Figures S54-S98, Tables S2-20**).

15.6.1 A-B-plane

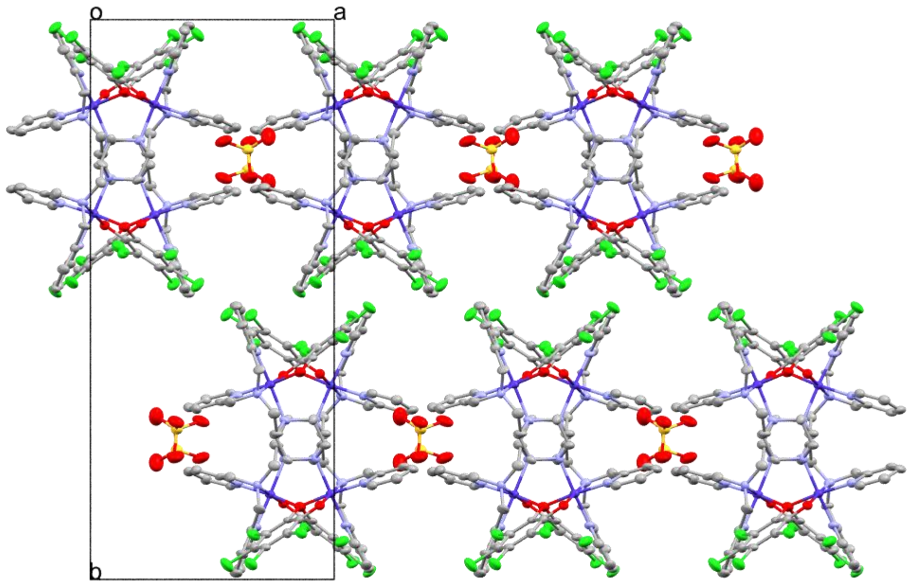

Figure S59: Crystal packing of **C1** view along c-axis. Lattice solvent molecules and hydrogen atoms are omitted for better visibility.

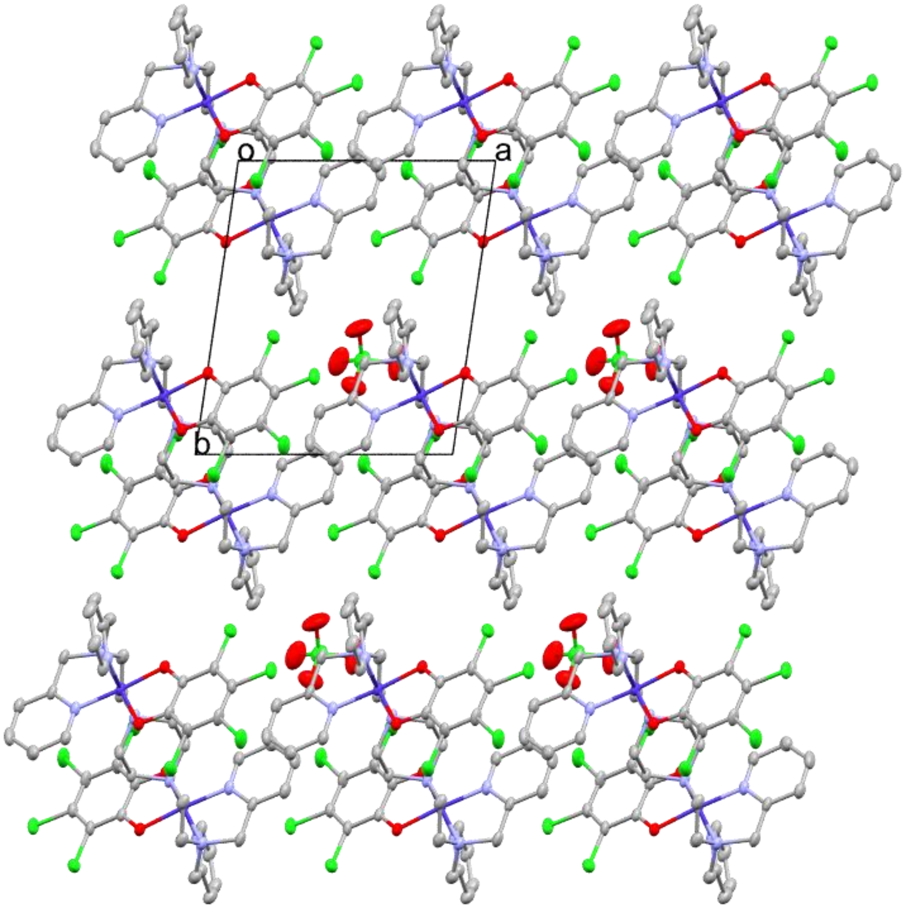

Figure S60: Crystal packing of **C2** view along c-axis. Lattice solvent molecules and hydrogen atoms are omitted for better visibility.

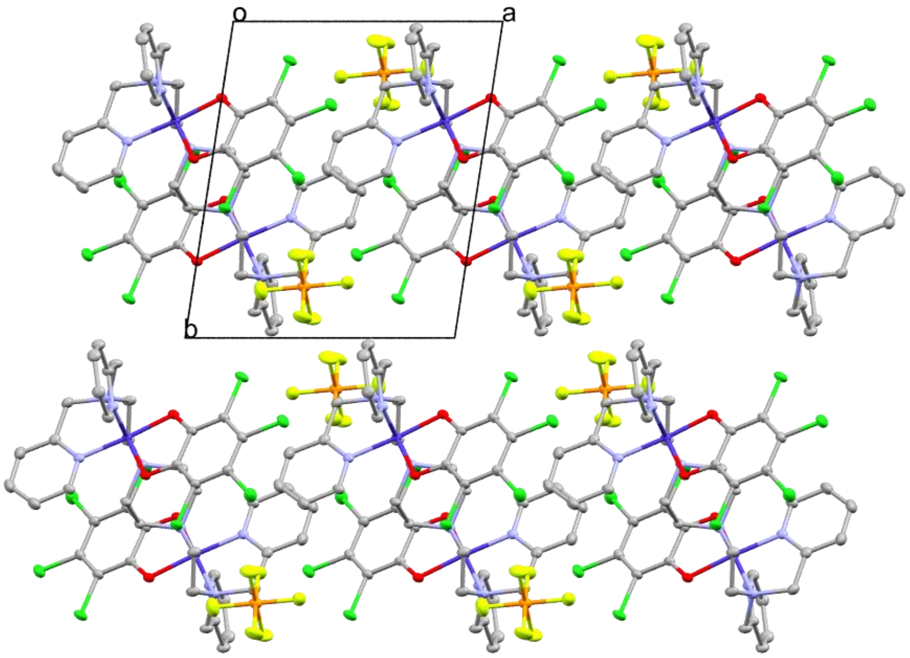

Figure S61: Crystal packing of **C3** view along c-axis. Lattice solvent molecules and hydrogen atoms are omitted for better visibility.

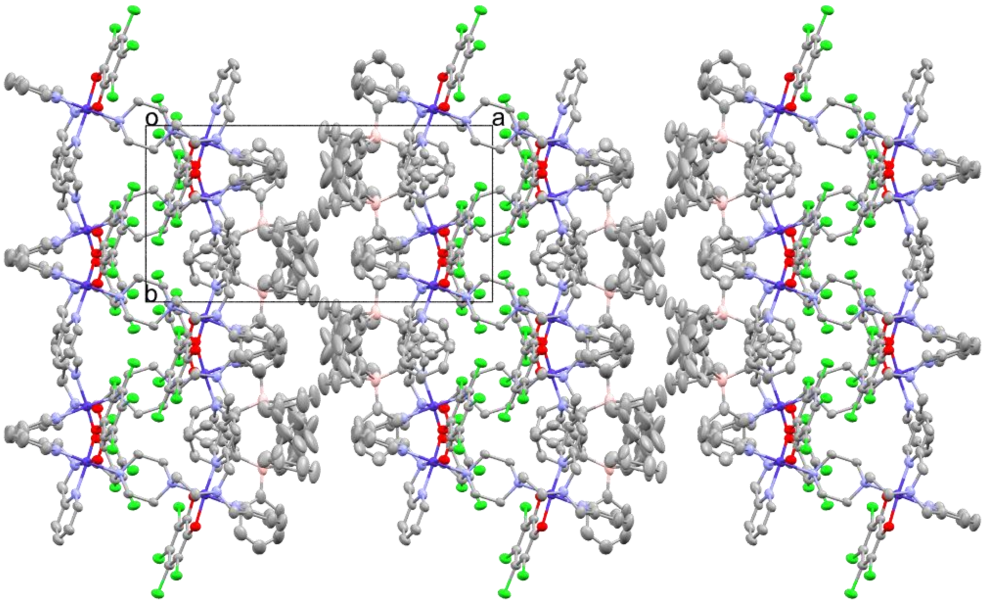

Figure S62: Crystal packing of **C4** view along c-axis. Lattice solvent molecules and hydrogen atoms are omitted for better visibility.

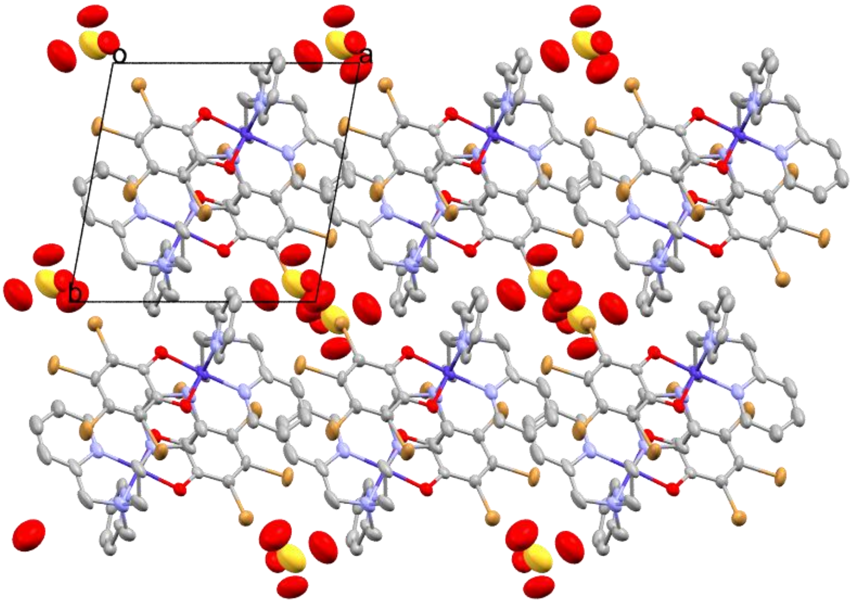

Figure S63: Crystal packing of **C5** view along c-axis. Lattice solvent molecules and hydrogen atoms are omitted for better visibility.

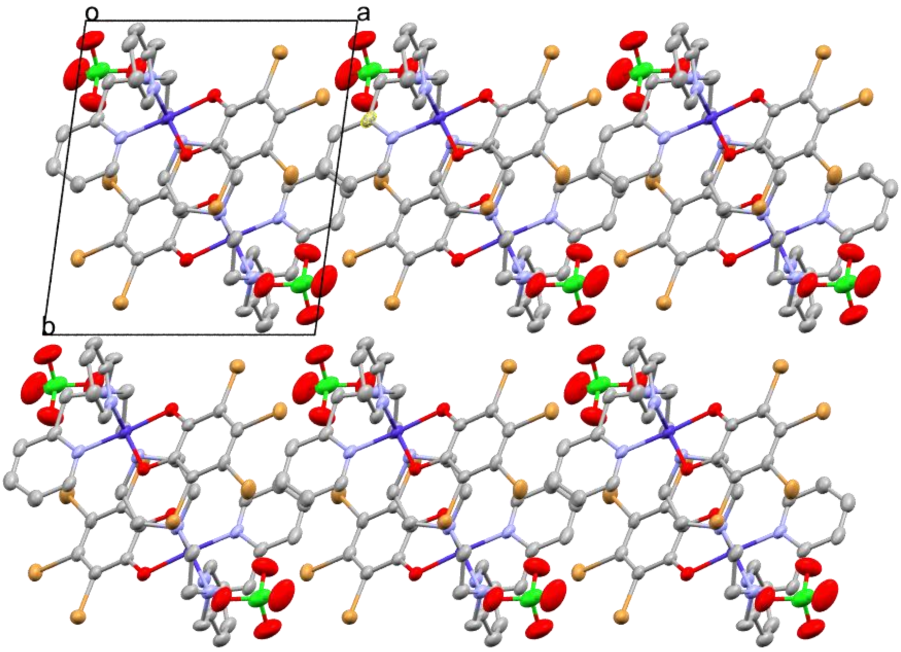

Figure S64: Crystal packing of **C6** view along c-axis. Lattice solvent molecules and hydrogen atoms are omitted for better visibility.

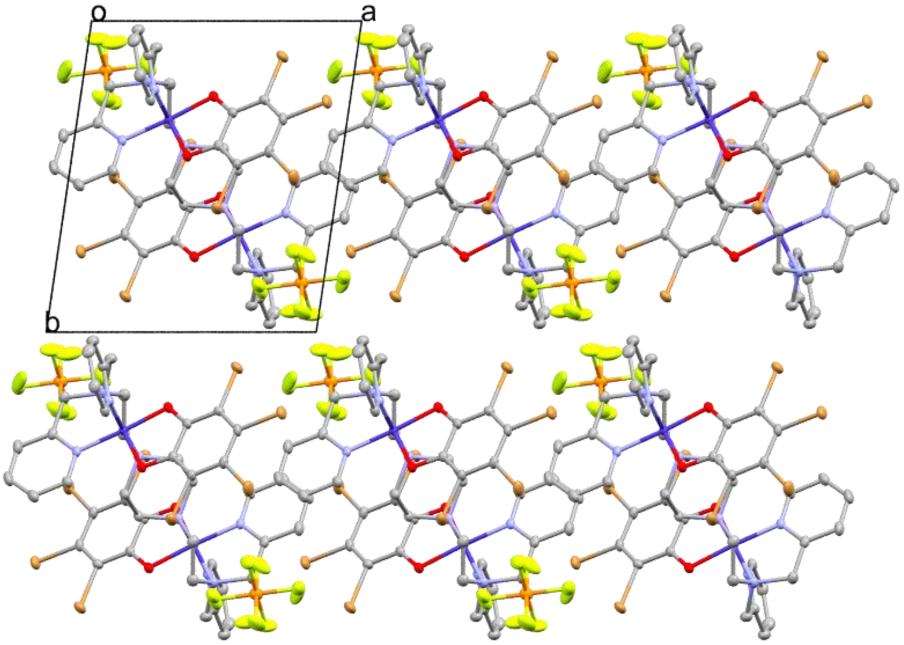

Figure S65: Crystal packing of **C7** view along c-axis. Lattice solvent molecules and hydrogen atoms are omitted for better visibility.

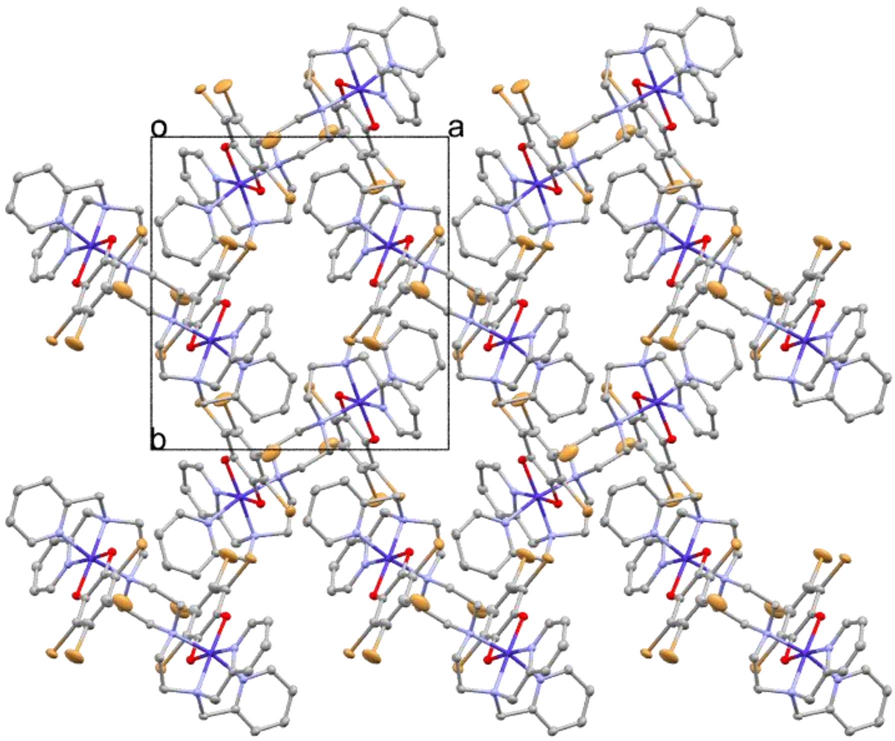

Figure S66: Crystal packing of **C8** view along c-axis. Lattice solvent molecules, anions and hydrogen atoms are omitted for better visibility.

15.6.2 B-C-Plane

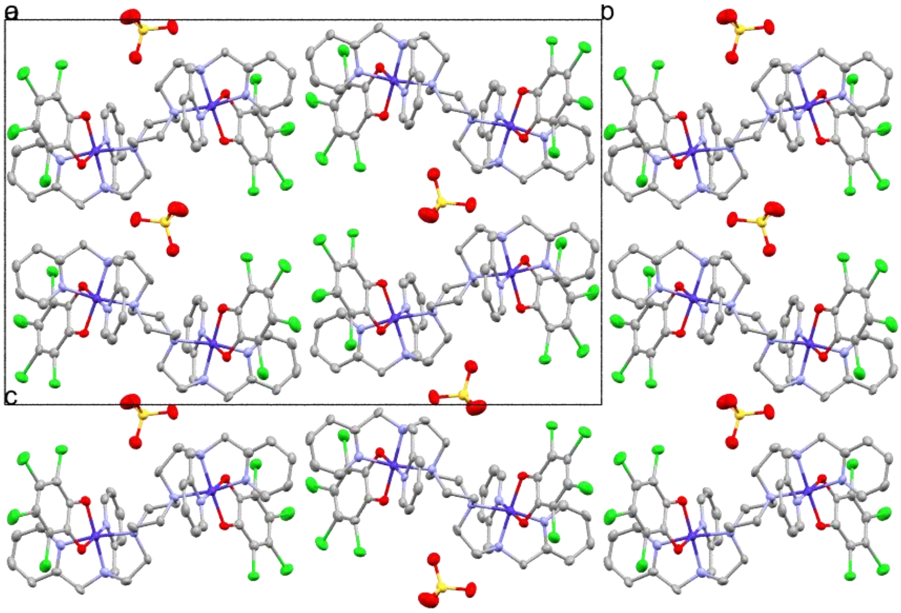

Figure S67: Crystal packing of **C1** view along a-axis. Lattice solvent molecules and hydrogen atoms are omitted for better visibility.

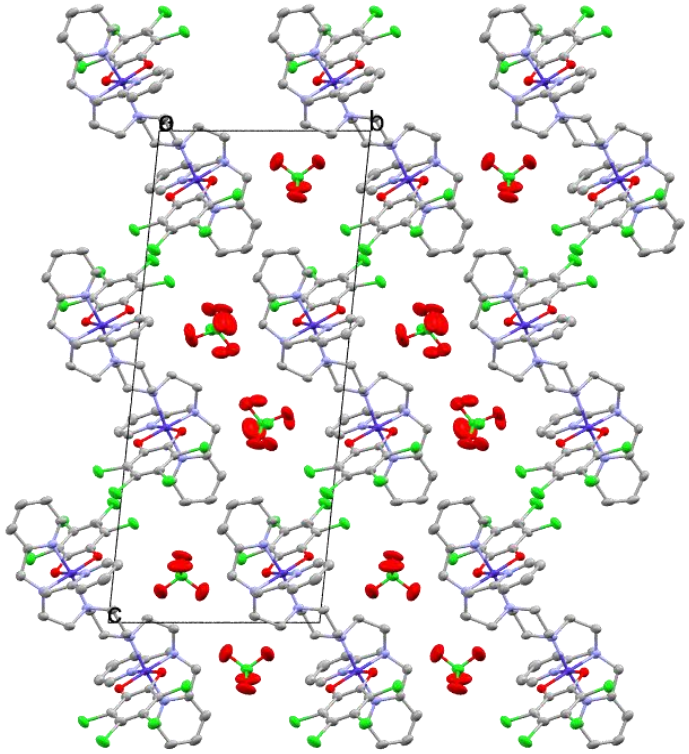

Figure S68: Crystal packing of **C2** view along a-axis. Lattice solvent molecules and hydrogen atoms are omitted for better visibility.

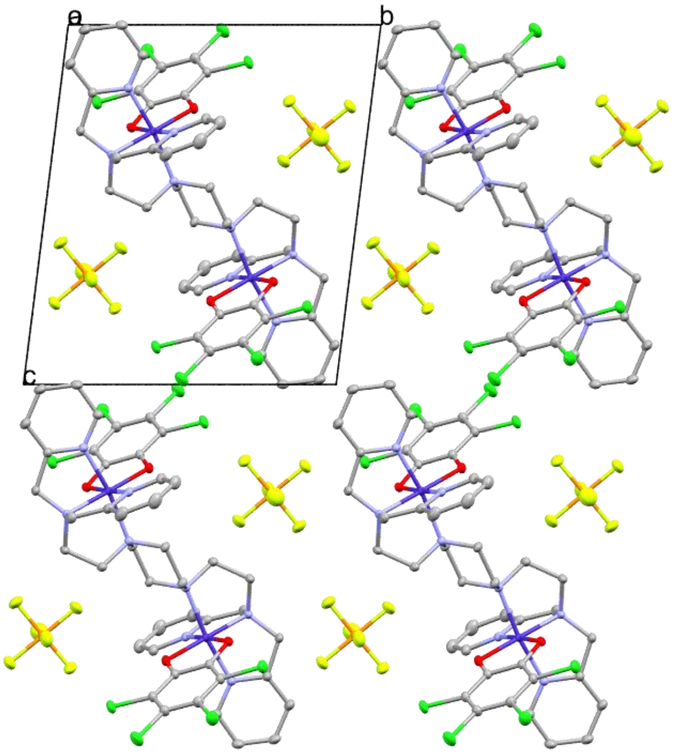

Figure S69: Crystal packing of **C3** view along a-axis. Lattice solvent molecules and hydrogen atoms are omitted for better visibility.

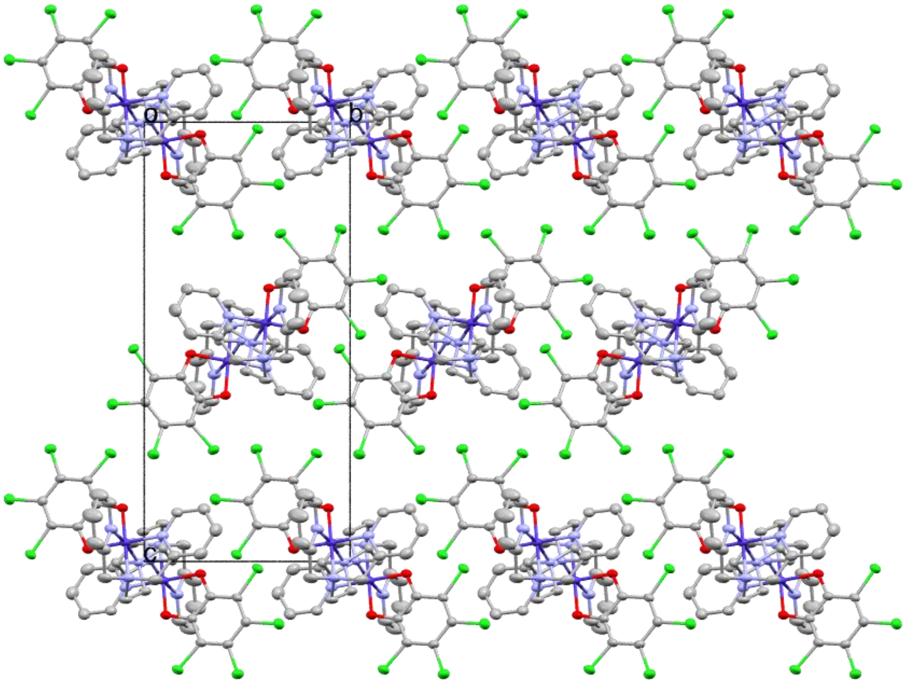

Figure S70: Crystal packing of **C4** view along a-axis. Lattice solvent molecules, anions and hydrogen atoms are omitted for better visibility.

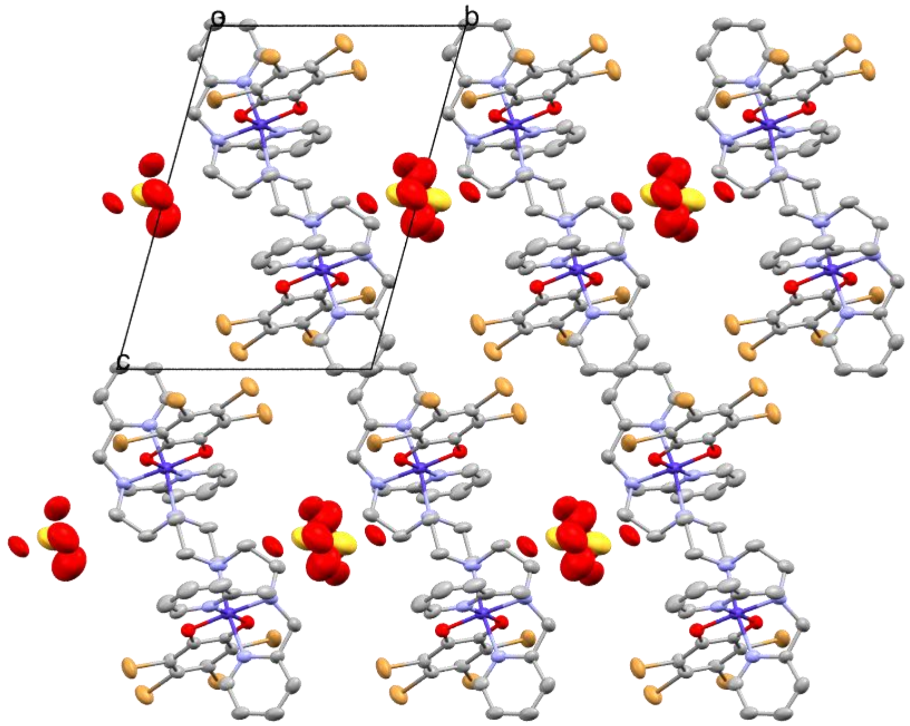

Figure S71: Crystal packing of **C5** view along a-axis. Lattice solvent molecules and hydrogen atoms are omitted for better visibility.

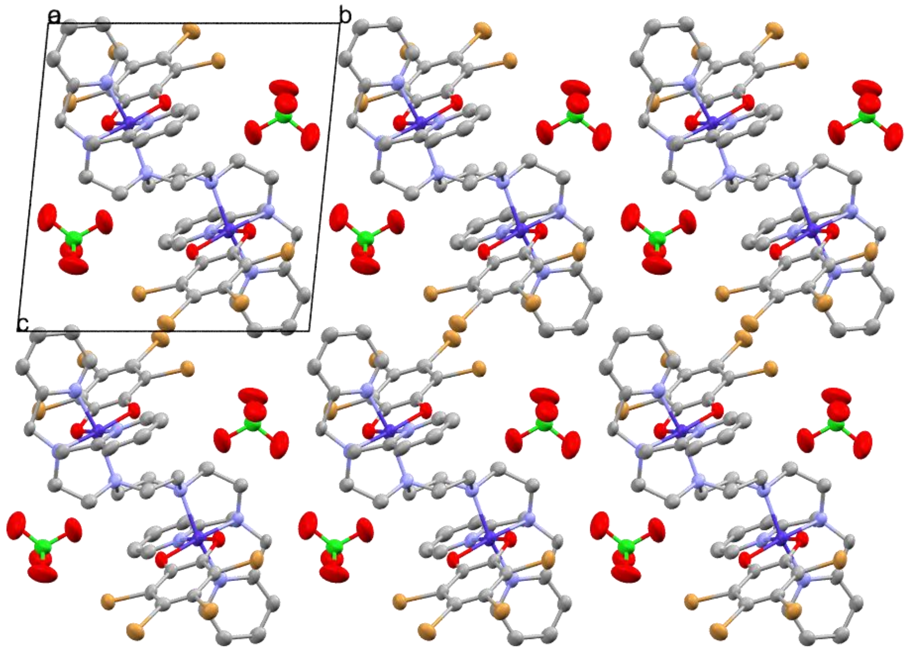

Figure S72: Crystal packing of **C6** view along a-axis. Lattice solvent molecules and hydrogen atoms are omitted for better visibility.

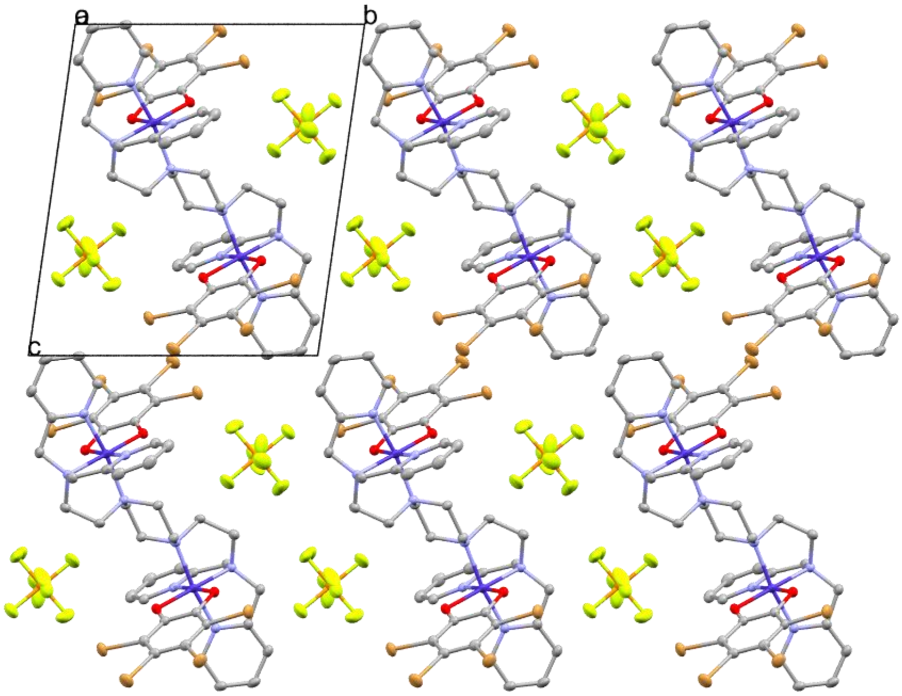

Figure S73: Crystal packing of **C7** view along a-axis. Lattice solvent molecules and hydrogen atoms are omitted for better visibility.

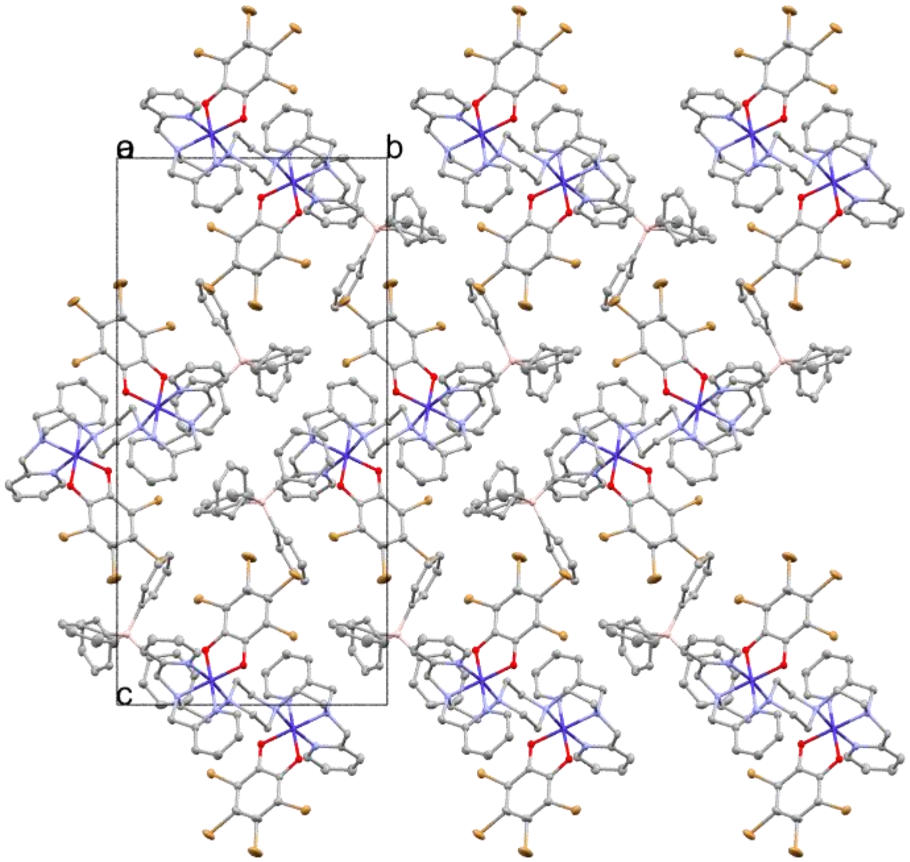

Figure S74: Crystal packing of **C8** view along a-axis. Lattice solvent molecules and hydrogen atoms are omitted for better visibility.

15.6.3 A-C-Plane

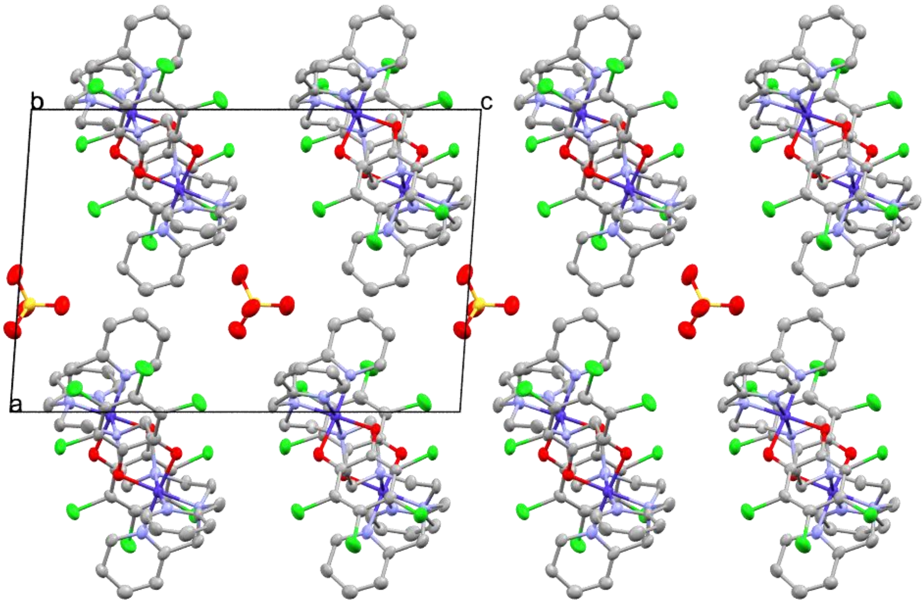

Figure S75: Crystal packing of **C1** view along b-axis. Lattice solvent molecules and hydrogen atoms are omitted for better visibility.

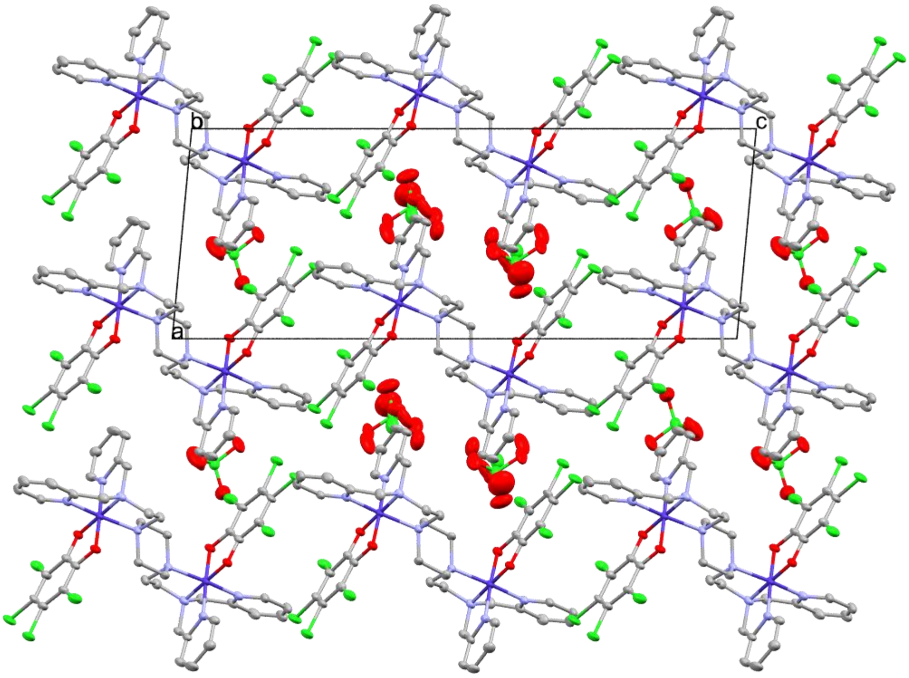

Figure S76: Crystal packing of **C2** view along b-axis. Lattice solvent molecules and hydrogen atoms are omitted for better visibility.

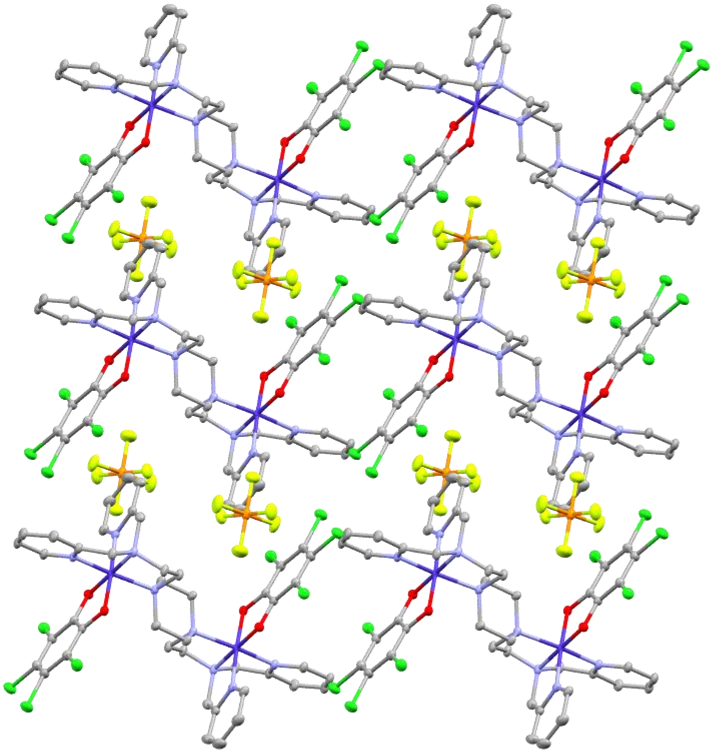

Figure S77: Crystal packing of **C3** view along b-axis. Lattice solvent molecules and hydrogen atoms are omitted for better visibility.

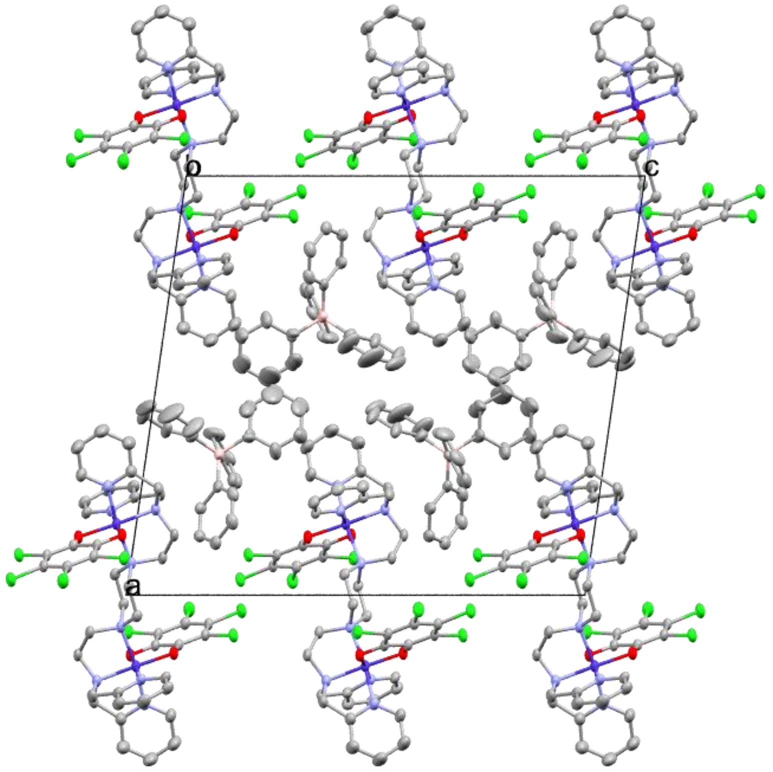

Figure S78: Crystal packing of **C4** view along b-axis. Lattice solvent molecules and hydrogen atoms are omitted for better visibility.

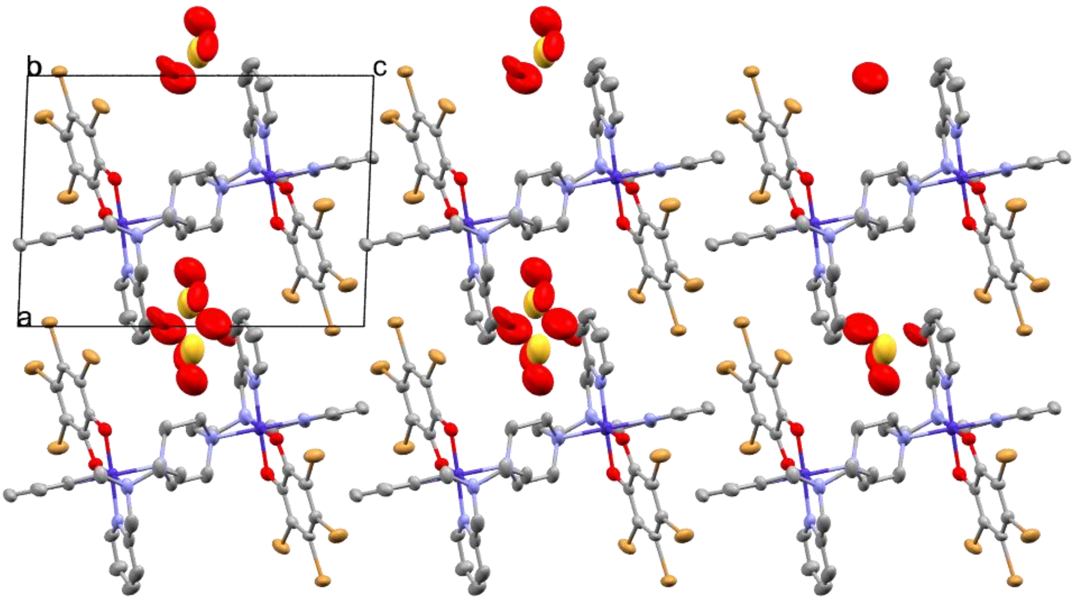

Figure S79: Crystal packing of **C5** view along b-axis. Lattice solvent molecules and hydrogen atoms are omitted for better visibility.

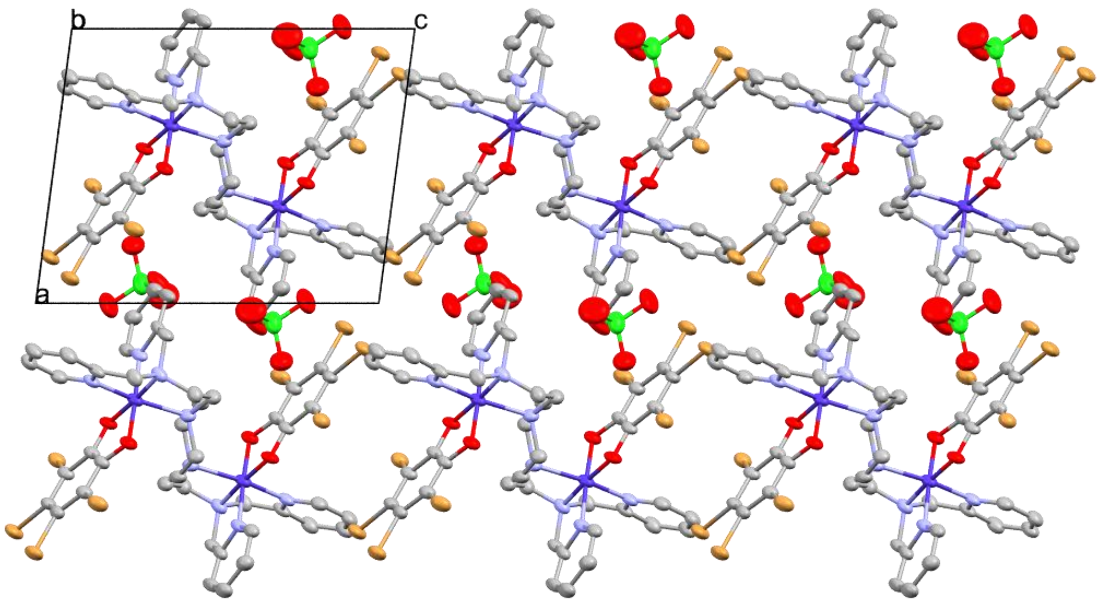

Figure S80: Crystal packing of **C6** view along b-axis. Lattice solvent molecules and hydrogen atoms are omitted for better visibility.

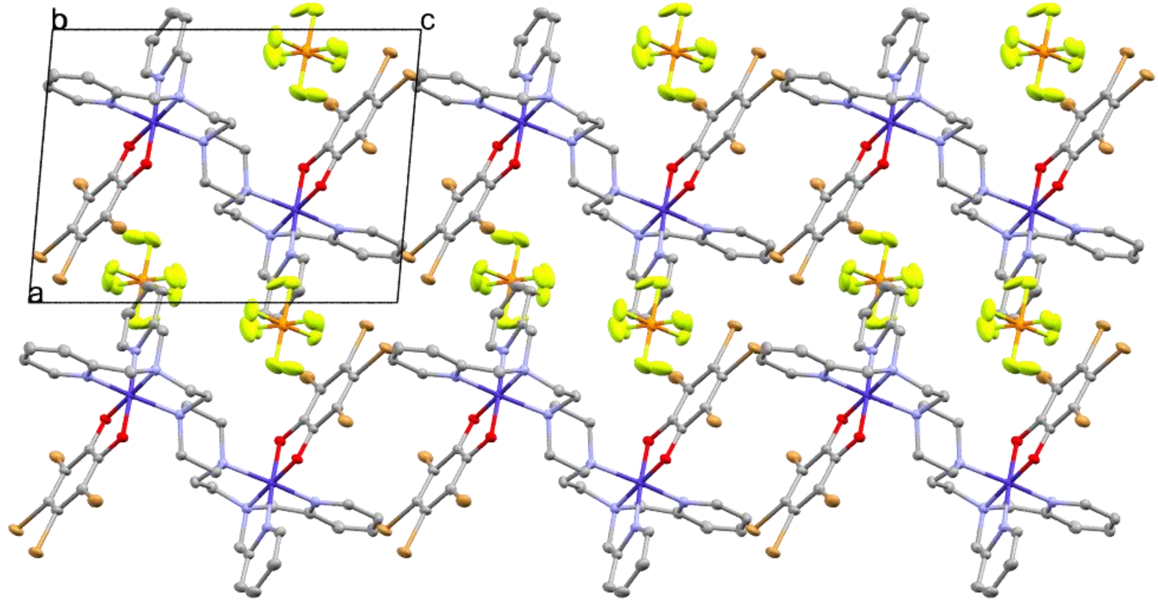

Figure S81: Crystal packing of **C7** view along b-axis. Lattice solvent molecules and hydrogen atoms are omitted for better visibility.

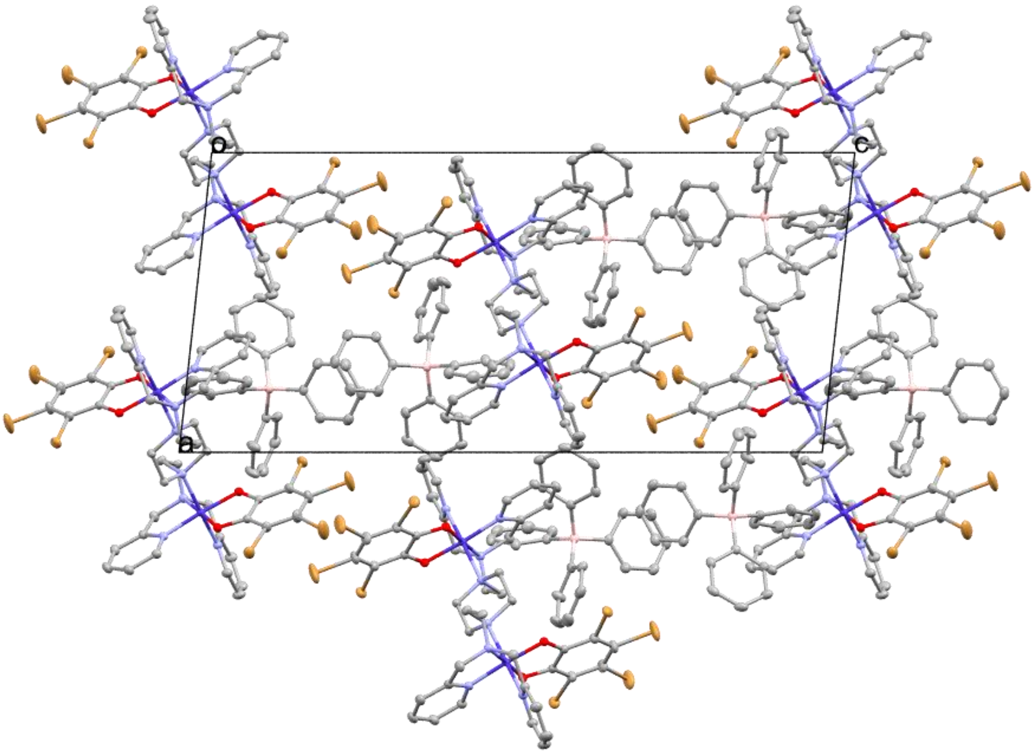

Figure S82: Crystal packing of **C8** view along b-axis. Lattice solvent molecules and hydrogen atoms are omitted for better visibility.

16. Cyclovoltammetric Scan Rate Studies

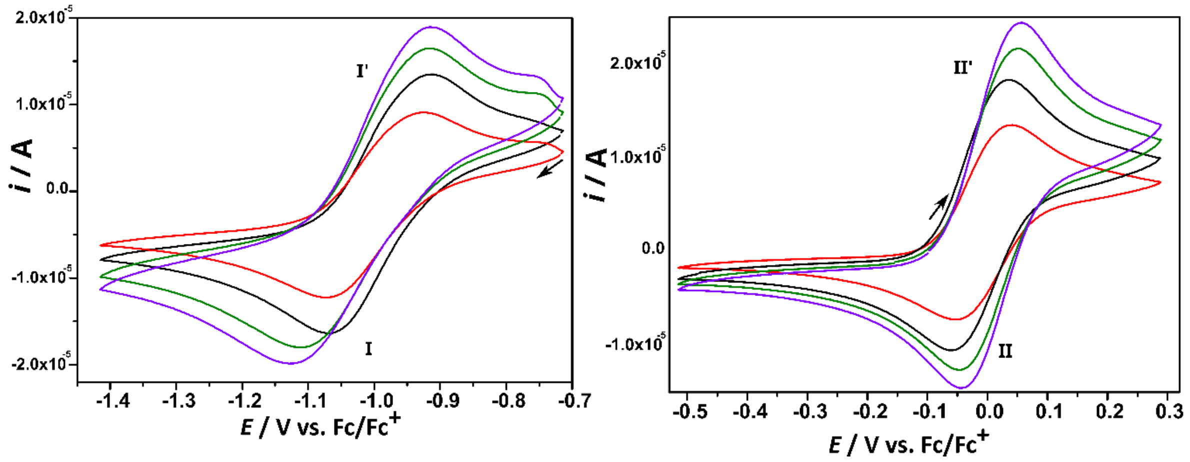

Figure S83: Cyclic voltammogram of previously reported **C9** $[Co_2(\mathbf{L})(dbucat)_2]$ $(ClO_4)_2 \cdot 1.5\ H_2O$[1] in acetonitrile ($1 \times 10^{-3}$ M, 0.1 M n-Bu_4PF_6) obtained with different scan rates (50 mV s^{-1} (red), 100 mV s^{-1} (black), 150 mV s^{-1} (green), 200 mV s^{-1} (purple)) of redox process (I/I') **left** and redox process (II/II') **right**, measured with a glassy carbon working electrode and a silver wire reference electrode, referenced against the internal redoxpair of Fc/Fc^+.

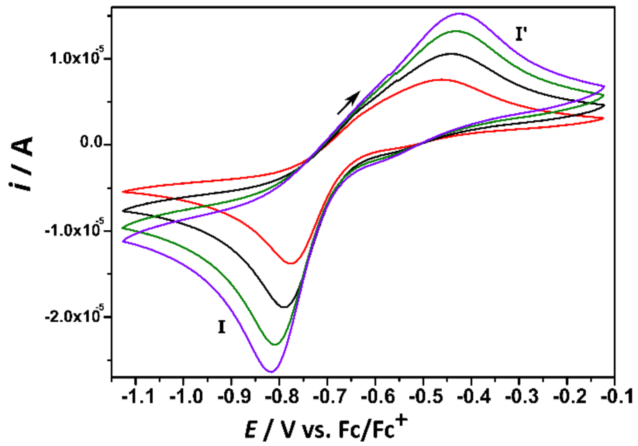

Figure S84: Cyclic voltammogram of **C2** $\cdot 3.5\ H_2O$ in acetonitrile (1×10^{-3} M, 0.1 M n-Bu_4PF_6) obtained at different scan rates (50 mV s^{-1} (red), 100 mV s^{-1} (black), 150 mV s^{-1} (green), 200 mV s^{-1} (purple)) of redox process (I/I') with a glassy carbon working electrode and a silver wire reference electrode referenced internally against the redoxpair Fc/Fc^+.

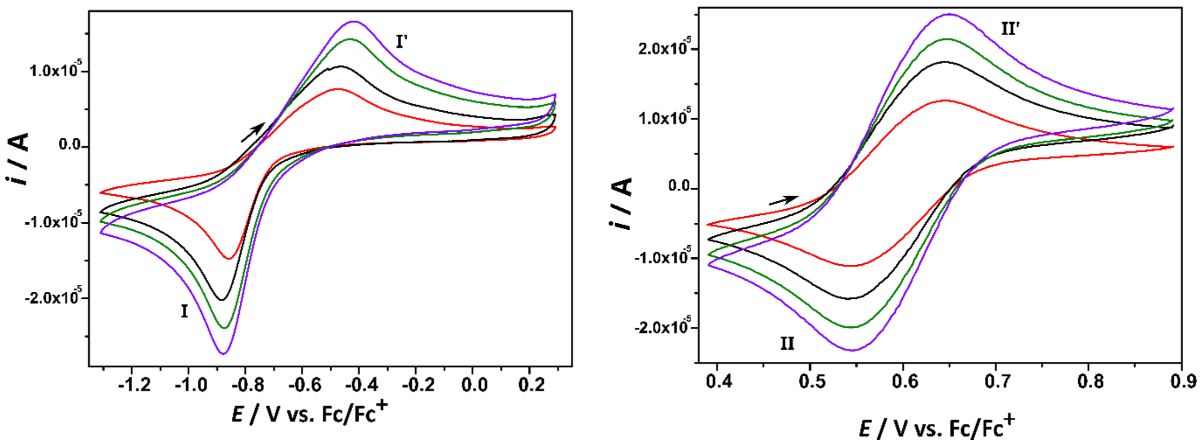

Figure S85: Cyclic voltammogram of **C6** ·1.5 H_2O in acetonitrile (1 x 10^{-3} M, 0.1 M n-Bu_4PF_6) obtained with different scan rates (50 mV s^{-1} (red), 100 mV s^{-1} (black), 150 mV s^{-1} (green), 200 mV s^{-1} (purple) of redox process (I/I') **left** and redox process (II/II') **right** with a glassy carbon working electrode and a silver wire reference electrode referenced internally against the redoxpair Fc/Fc^{+}.

17. Magnetic Susceptibility Measurements

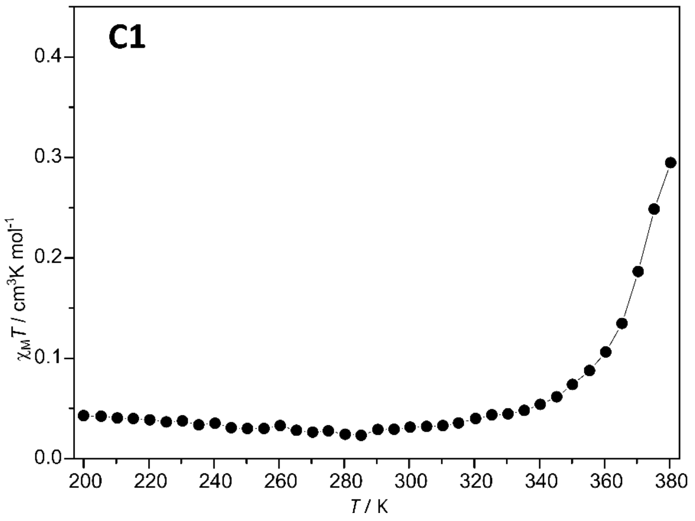

Figure S86: Temperature dependent $\chi_M T$ plot of **C1**· 8 H_2O over the temperature range of 200-380 K.

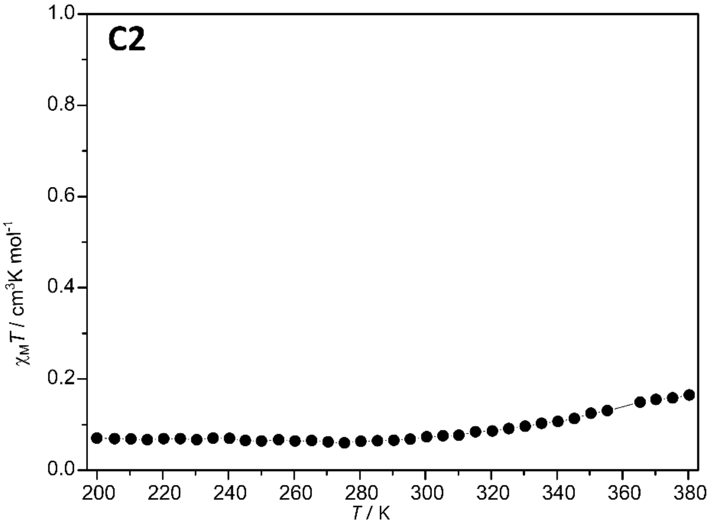

Figure S87: Temperature dependent $\chi_M T$ plot of **C2**· 3.5 H_2O over the temperature range of 200-380 K.

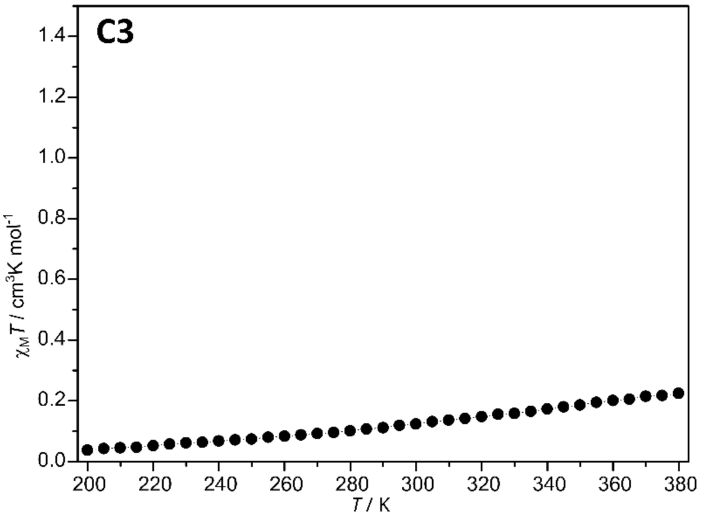

Figure S88: Temperature dependent $\chi_M T$ plot of **C3**· 6.5 H_2O over the temperature range of 200-380 K.

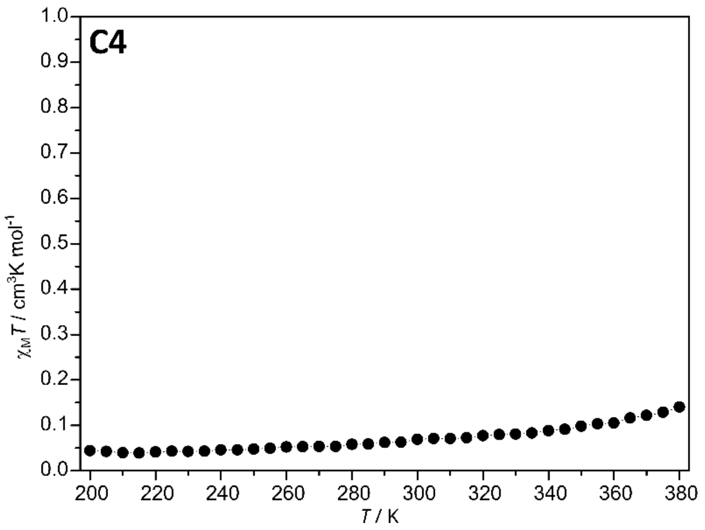

Figure S89: Temperature dependent $\chi_M T$ plot of **C4**· 2 H_2O over the temperature range of 200-380 K.

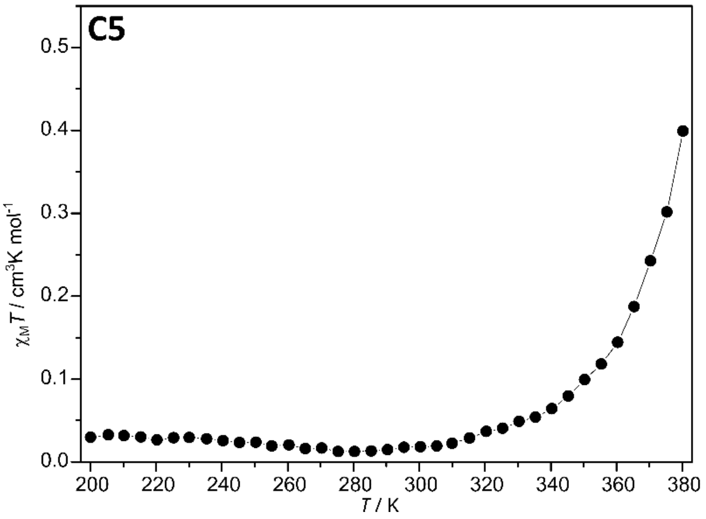

Figure S90: Temperature dependent $\chi_M T$ plot of **C5**· 5.5 H_2O· 1 $CHCl_3$ over the temperature range of 200-380 K.

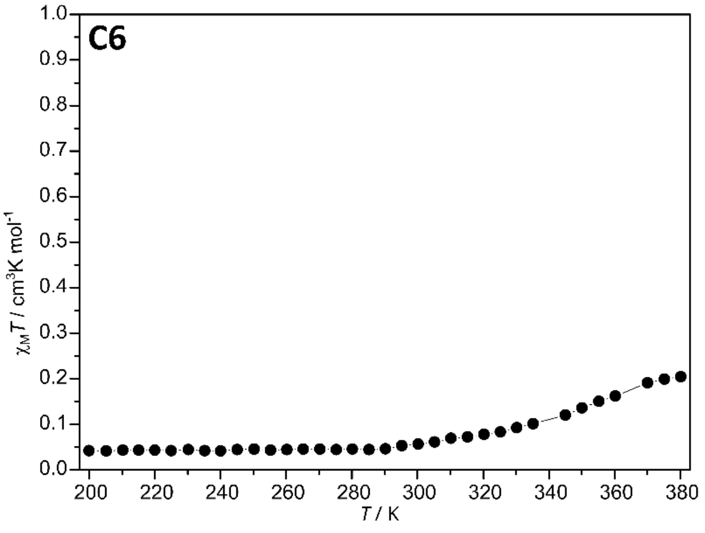

Figure S91: Temperature dependent $\chi_M T$ plot of **C6**· 1.5 H_2O over the temperature range of 200-380 K.

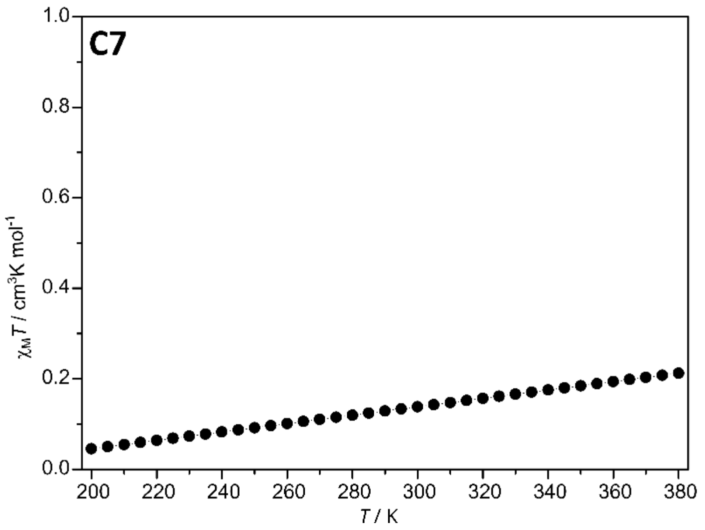

Figure S92: Temperature dependent $\chi_M T$ plot of **C7**· 1.25 H_2O over the temperature range of 200-380 K.

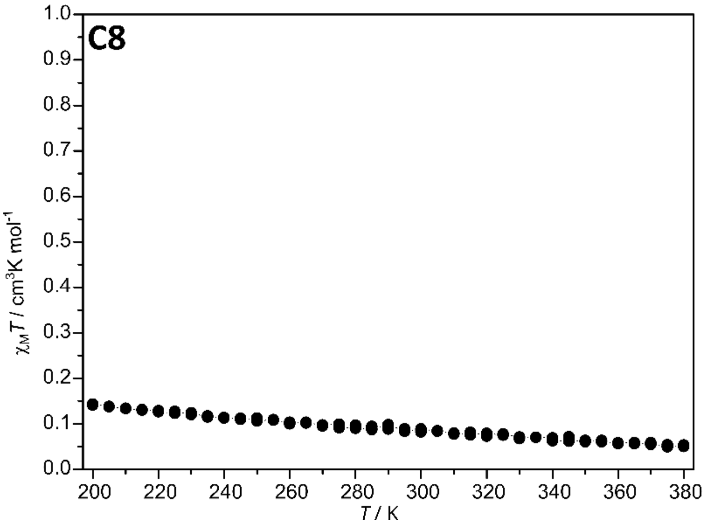

Figure S93: Temperature dependent $\chi_M T$ plot of **C8**· 1 H_2O over the temperature range of 200-380 K.

18. UV-VIS-Spectroscopy

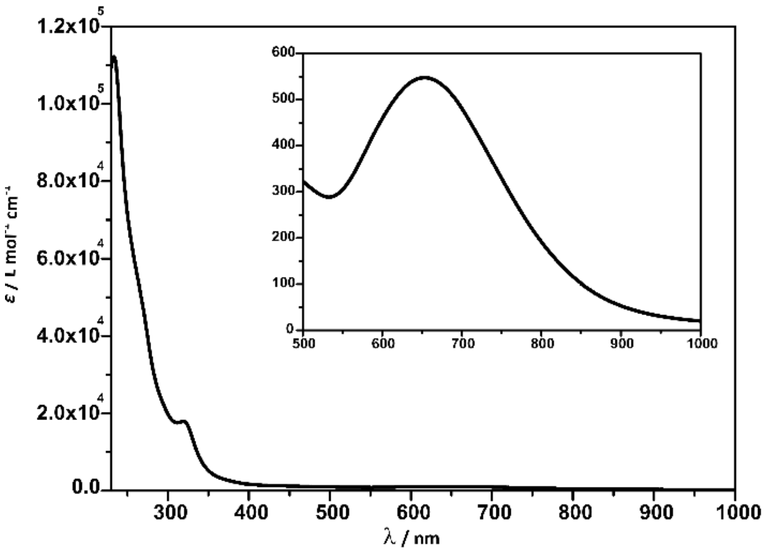

Figure S94: UV-VIS absorption spectra for **C2** 0.1 mM in MeCN with a zoomed-in section from 500 to 1000 nm at a higher concentration of 1mM.

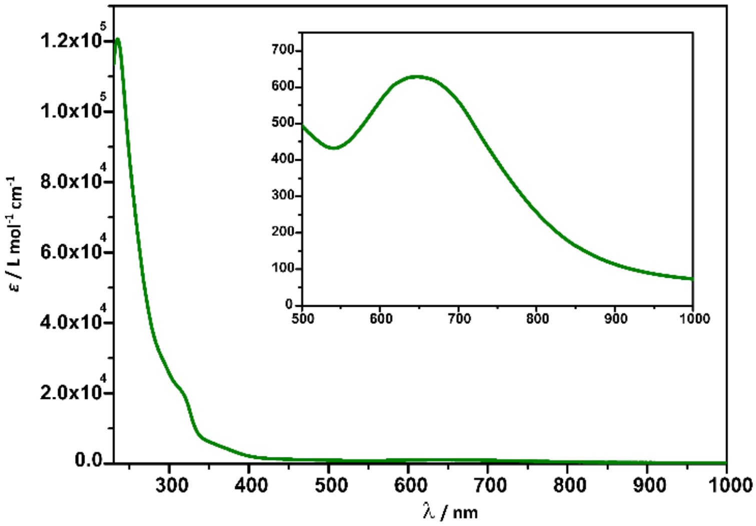

Figure S95: UV-VIS absorption spectra for **C6** 0.01 mM in MeCN with a zoomed-in section from 500 to 1000 nm at a higher concentration of 1mM.

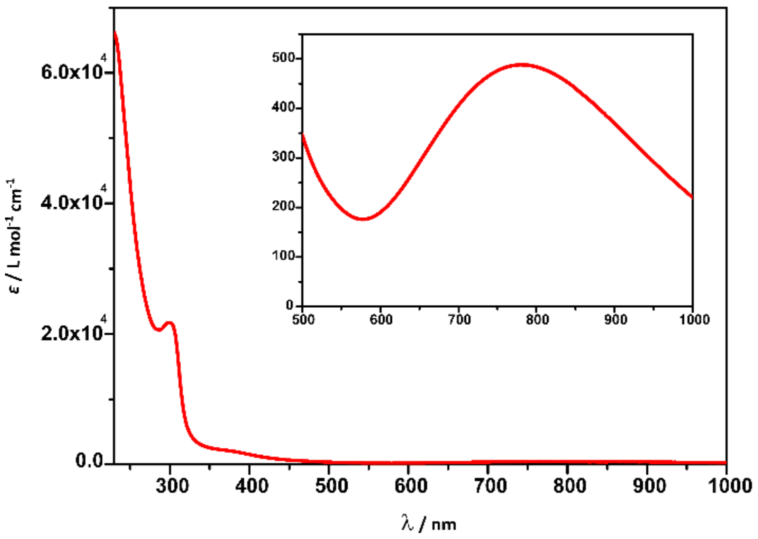

Figure S96: UV-VIS absorption spectra of $[Co_2(\mathbf{L})(dbucat)_2]$ $(ClO_4)_2 \cdot 1.5\ H_2O$ 0.01 mM in MeCN with a zoomed-in section from 500 to 1000 nm at a higher concentration of 1mM.

Table S31: Tabulated molar extinction coefficients in (L $mol^{-1}cm^{-1}$) at their respective wavelength in nm for complexes **C2** and **C6**.

C2	C6	$[Co_2(L)(dbucat)_2](ClO_4)_2 \cdot 1.5\ H_2O$
112185 (233 nm)	120632 (236 nm)	21746 (299 nm)
17921 (319 nm)	20065 (318 nm)	2058 (378 nm)
548 (654 nm)	629 (652 nm)	488 (781 nm)

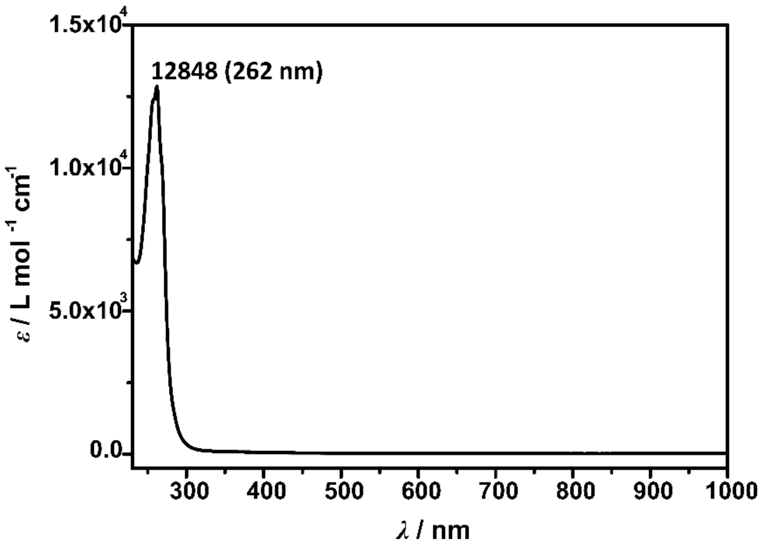

Figure S97: UV-VIS absorption spectra of **L** 0.1 mM in MeCN at RT.

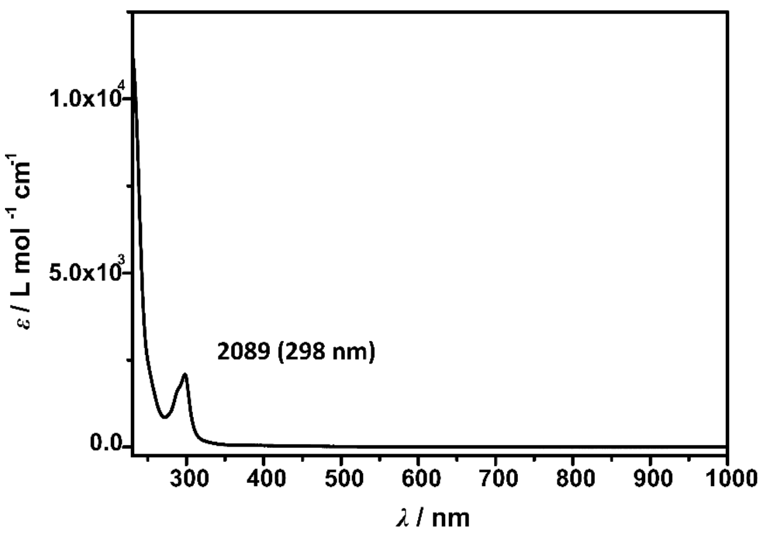

Figure S98: UV-VIS absorption spectra of **Cl4-cat** 0.1 mM in MeCN at RT.

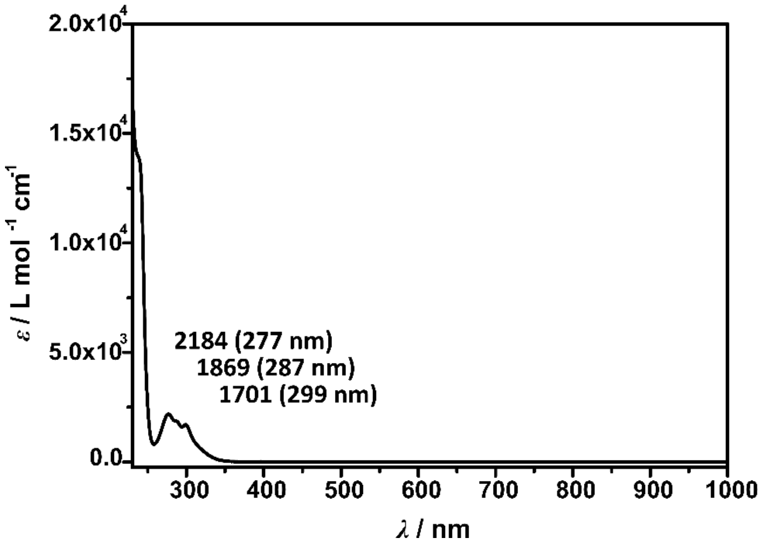

Figure S99: UV-VIS absorption spectra of **Br4-cat** 0.1 mM in MeCN at RT.

19. Evans Method NMR

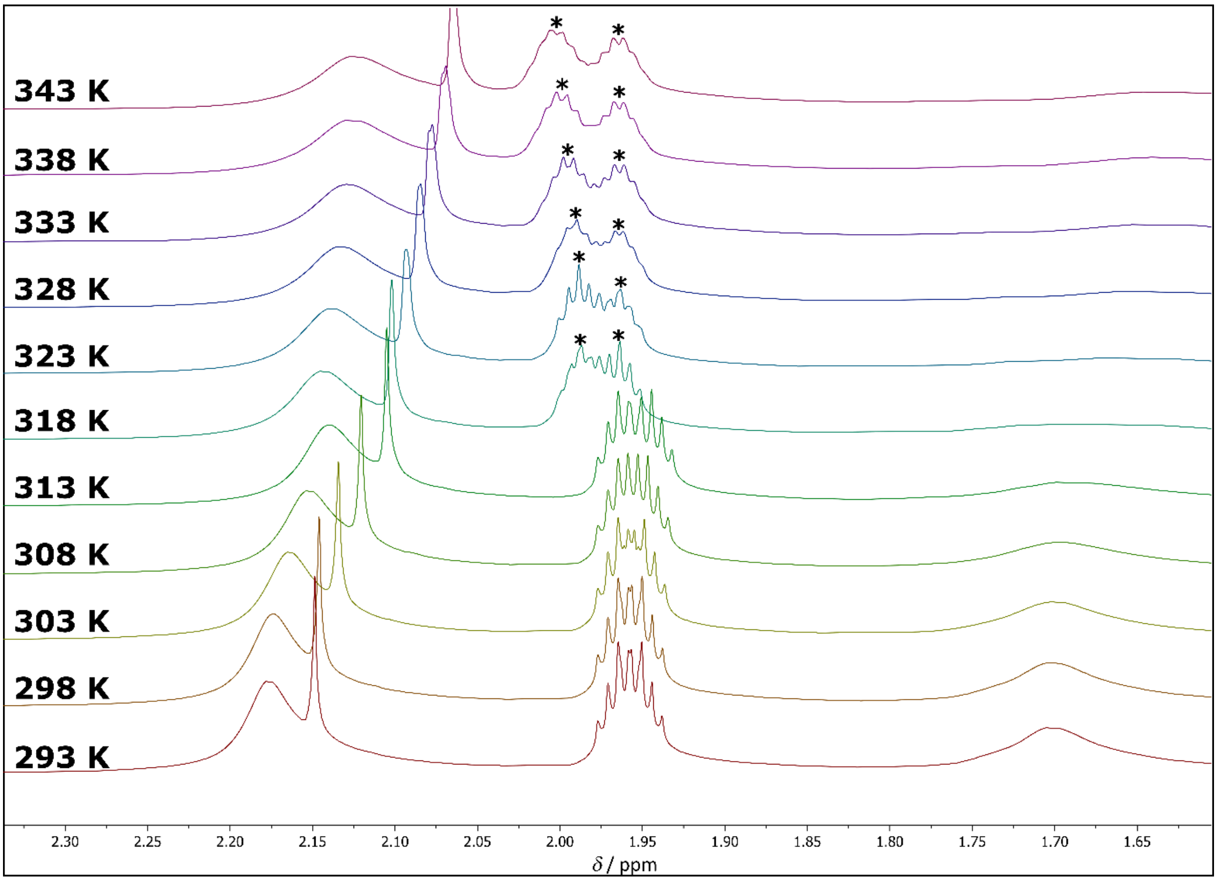

Figure S100: Evans method ^{1}H NMR (6 mM) at variable temperatures of previously reported [Co_2(**L**)(dbucat)$_2$] (ClO_4)$_2$ · 1.5 H_2O[1] over a temperature range of 293K to 343K in acetonitrile [400 MHz] in 5K steps.

Table S32: Evans method ^{1}H NMR Data for of previously reported [Co_2(**L**)(dbucat)$_2$] (ClO_4)$_2$ · 1.5 H_2O[1] over a temperature range of 293K to 343K in acetonitrile [400MHz] in 5K steps.

***T* / K**	**$\chi_M T$ / cm^{3}Kmol^{-1}**	**Δf / Hz**
293	0.194	0.00
298	0.207	0.75
303	0.221	1.50
308	0.236	2.30
313	0.254	3.25
318	0.345	9.07
323	0.366	10.1
328	0.393	11.3
333	0.416	12.4
338	0.445	13.9
343	0.478	15.2

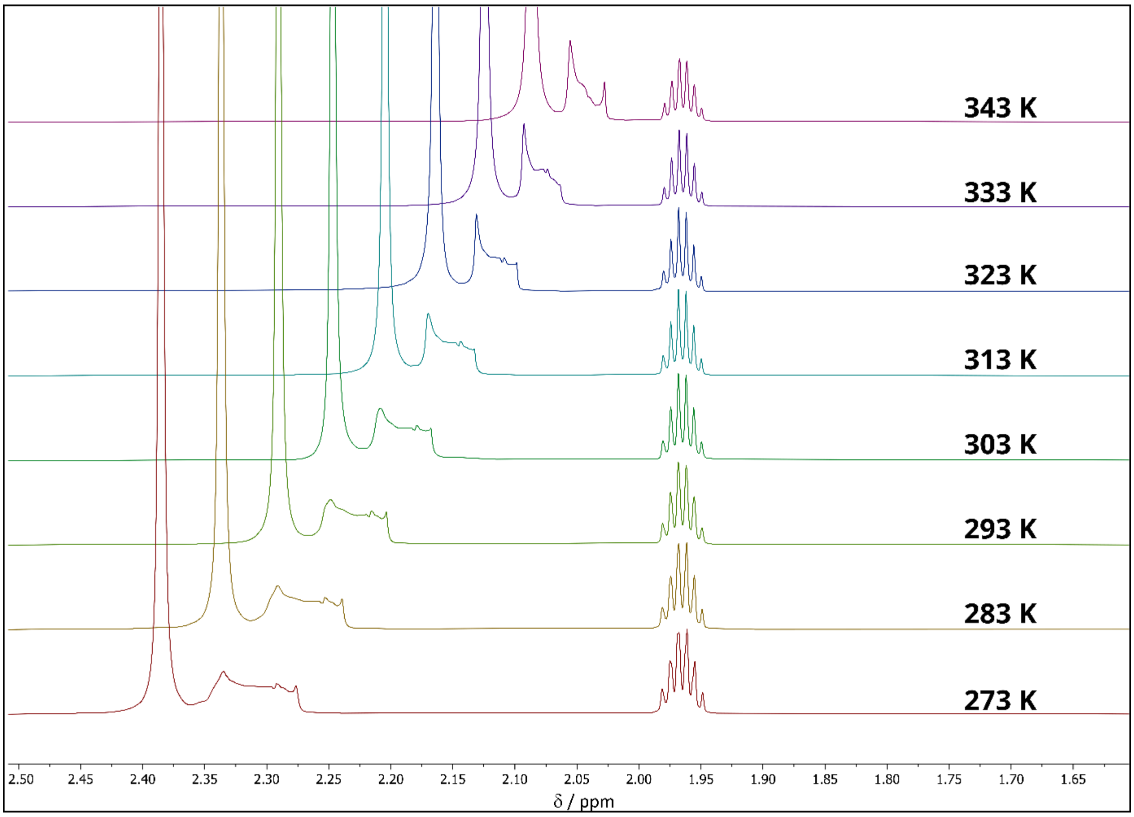

Figure S101: Evans method ^{1}H NMR (6 mM) at variable temperatures of **C3** over a temperature range of 273K to 343K in acetonitrile [400 MHz] in 10K steps staying diamagnetic, showing no VT transition over the observed temperature range.

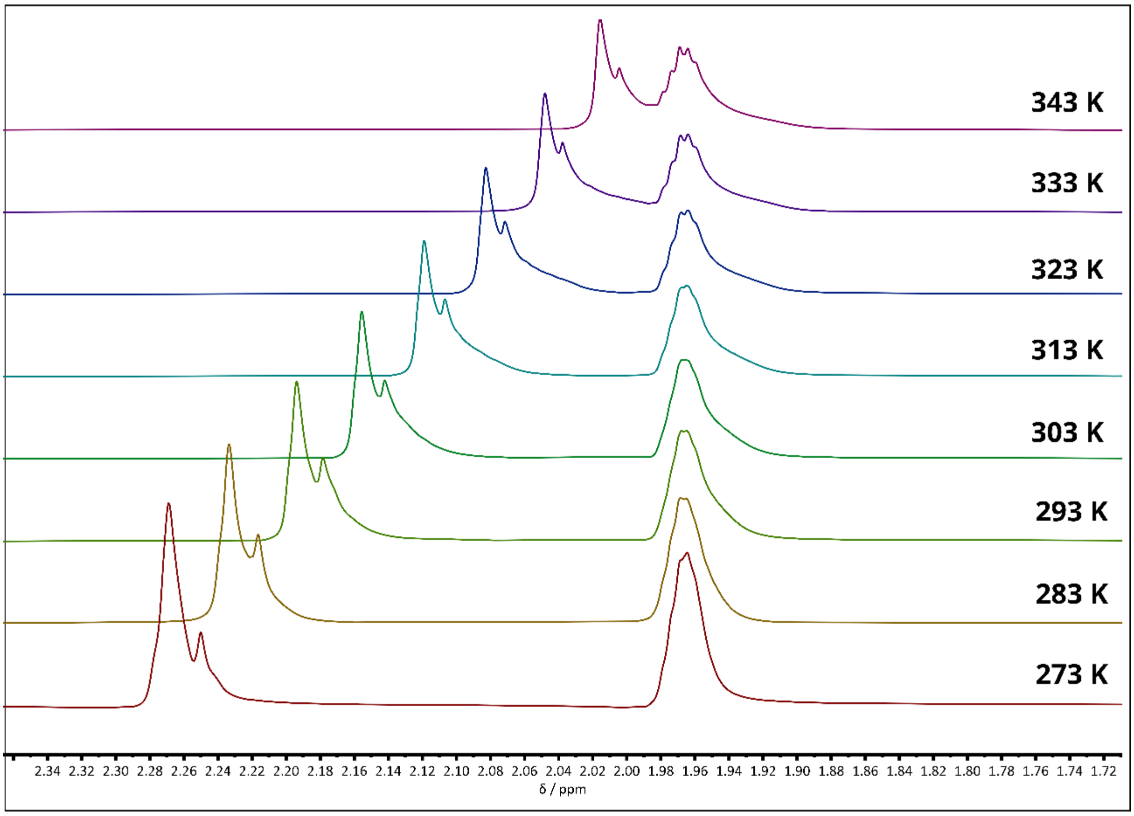

Figure S102: Evans method ^{1}H NMR (6 mM) at variable temperatures of **C7** over a temperature range of 273K to 343K in acetonitrile [400 MHz] in 10K steps staying diamagnetic, showing no VT transition over the observed temperature range.

[1] S. Sundaresan, M. Diehl, L. M. Carrella, and E. Rentschler, ‘Triggering of Valence Tautomeric Transitions in Dioxolene-Based Cobalt Complexes Influenced by Ligand Substituents, Co-ligands, and Anions’, *Magnetochemistry*, **2022**, *8*(9), pp. 1–12, DOI: 10.3390/magnetochemistry8090109.
